# Safety and immunogenicity of a new formulation of a pentavalent DTwP-HepB-Hib vaccine in healthy Indian infants–A randomized study

**DOI:** 10.1371/journal.pone.0284898

**Published:** 2023-08-15

**Authors:** Naveena Aloysia D’Cor, Prashanth Siddaiah, Satyajit Mohapatra, Sangappa Malappa Dhaded, Padmavathi I. V., Sonali Kar, Tripathi V. N., Prasad Muley, Jugesh Chhatwal, Badri Narayan Patnaik, Emmanuel Vidor, Annick Moureau, Dhaval M. Patel, Venkata Jayanth Midde, Sathish Reddy Jagga, Satyanarayana Peesapati, Fernando Noriega

**Affiliations:** 1 Sanofi, Hyderabad, India; 2 Dept. of Pediatrics, Mysore Medical College and Research Institute, Mysore, India; 3 Dept. of Pharmacology, SRM Medical College Hospital & Research Center, Chennai, India; 4 Dept. of Pediatrics, KLES Dr Prabhakar Kore Hospital & Medical Research Centre, Belagavi, India; 5 Dept. of Pediatrics, Government Victoria Hospital, Visakhapatnam, India; 6 Dept. of Community Medicine, Kalinga Institute of Medical Sciences, Bhubaneswar, India; 7 Dept. of Pediatrics, Prakhar Hospital Pvt. Ltd., Kanpur, India; 8 Dept. of Pediatrics, SBKS Medical College, Vadodara, India; 9 Dept. of Pediatrics, Christian Medical College & Hospital, Ludhiana, India; 10 Sanofi, Mumbai, India; 11 Sanofi, Lyon, France; 12 Sanofi, Swiftwater, PA, United States of America; Public Health England, UNITED KINGDOM

## Abstract

**Background:**

Pentavalent vaccines (DTP-HepB-Hib) have been introduced in many countries in their routine public immunization programmes to protect against diphtheria (D), tetanus (T), pertussis (P), hepatitis B (Hep B) and *Hemophilus influenzae* type b (Hib) diseases. This study compared the safety and immunogenicity of a new formulation of a whole-cell *Bordetella pertussis* (wP) based pentavalent vaccine (DTwP-HepB-Hib). The new formulation was developed using well-characterized hepatitis B and pertussis whole cell vaccine components.

**Methods:**

This was a phase III, observer-blind, randomized, non-inferiority, multi-center study conducted in India among 460 infants who were followed up for safety and immunogenicity for 28 days after administration of three doses of either investigational or licensed comparator formulations at 6–8, 10–12 and 14–16 weeks of age.

**Results:**

The investigational formulation of DTwP-HepB-Hib vaccine was non-inferior to the licensed formulation in terms of hepatitis B seroprotection rate (% of subjects with HepB antibodies ≥10mIU/mL were 99.1% versus 99.0%, respectively, corresponding to a difference of 0.1% (95% CI, -2.47 to 2.68)) and pertussis immune responses (adjusted geometric mean concentrations of antibodies for anti-PT were 76.7 EU/mL versus 63.3 EU/mL, with a ratio of aGMTs of 1.21 (95% CI, 0.89–1.64), and for anti-FIM were 1079 EU/mL versus 1129 EU/mL, with a ratio of aGMTs of 0.95 (95% CI, 0.73–1.24), respectively). The immune responses to other valences (D, T, and Hib) in the two formulations were also similar. The safety profile of both formulations was found to be similar and were well tolerated.

**Conclusions:**

The investigational DTwP-HepB-Hib vaccine formulation was immunogenic and well-tolerated when administered as three dose primary series in infants.

**Clinical trial registration:**

Clinical Trials Registry India number: CTRI/2018/12/016692.

## Introduction

Diphtheria, tetanus, pertussis (DTP) combination vaccines were first introduced in 1948 [1]. Since several decades, they have been the cornerstone of immunization programs to vaccinate infants and children. Subsequently other vaccines have been added to the combination to rapidly achieve better coverage against other childhood pathogens like hepatitis B (HepB) and *Haemophilus influenzae* type b (Hib). There are several advantages of combination vaccines including fewer injections, increased compliance and timeliness to vaccination, higher immunization coverage, less storage space, simplified acquisition and cold chain logistics and reduced administration costs [2].

SHAN 5^®^ is a whole cell *Bordetella pertussis* (wP) based pentavalent vaccine (DTwP-HepB-Hib) developed and manufactured by Sanofi Healthcare India Pvt. Ltd. (SHIPL) (formerly Shantha Biotechnics Pvt. Ltd.). It was first licensed in India and subsequently registered in more than 20 countries worldwide. Since its initial development, different generations of the DTwP-HepB-Hib vaccine formulations have been developed by SHIPL. Clinical trials conducted with different generations of the vaccine demonstrated non-inferior immunogenicity when compared with licensed comparator vaccines [3, 4]. The 1^st^ generation vaccine formulation was developed, and all the five valences were sourced from SHIPL [3]. It was licensed in India in 2007 and WHO pre-qualification status for the vaccine was obtained in 2008. In July 2010, the 1^st^ generation vaccine was delisted from WHO Pre-Qualification (WHO PQ) list due to field observations of whitish precipitates sticking to the vaccine vials and not re-suspendable after shaking [5]. The 2^nd^ generation vaccine formulation was developed to resolve this issue by replacing the pertussis component sourced from SHIPL with the pertussis component sourced from one Sanofi site at Marcy l’Etoile (MLE), France. The 2^nd^ generation vaccine was licensed in India in March 2014 [6] and the WHO re-accorded pre-qualification status in April 2014 [7].

SHIPL developed a new investigational formulation of the DTwP-HepB-Hib vaccine as part of the manufacturing life cycle management of the vaccine following a reshape of industrial strategy. The new investigational DTwP-HepB-Hib formulation differs from the licensed 2^nd^ generation DTwP-HepB-Hib vaccine used as comparator in the study with respect to the sources of two vaccine valences (HepB and pertussis) as described below. The source for rest of the valences D, T and Hib are the same in both these formulations of the DTwP-HepB-Hib vaccine.

*Bordetella pertussis* antigens used in the 2^nd^ generation DTwP-HepB-Hib vaccine formulation are sourced from France (Sanofi, MLE). Whereas for the investigational DTwP-HepB-Hib vaccine formulation, the *B*. *pertussis* antigens are manufactured at SHIPL from the MLE *B*. *pertussis* seed strains and fermentation and inactivation technologies transferred from MLE; therefore, the wP antigens used in both these formulations of the vaccine are derived from the historical *B*. *pertussis* seed strains used by Sanofi, France for manufacturing its historical DTwP trivalent (DTCoq^®^), DTwP-IPV quadrivalent (TETRACOQ^®^) and DTwP-IPV/Hib pentavalent (PENTACOQ^®^) vaccines that were distributed for decades in France and many European and non-European countries [8]. The HepB antigen (recombinant Hepatitis B surface Antigen [rHBsAg]) used for the 2^nd^ generation DTwP-HepB-Hib vaccine is sourced from SHIPL using the *Pichia pastoris* yeast growth platform [9] while the source of rHBsAg for the investigational DTwP-HepB-Hib vaccine is sourced from the Sanofi site at Pilar (Buenos Aires province), Argentina using the *Hansenula polymorpha* yeast growth platform [10]. This rHBsAg antigen has been extensively characterized biochemically [11–16] and is manufactured since 2004. Data from clinical trials showed this HBsAg to be highly immunogenic and safe when used in standalone vaccines [17] and in one hexavalent DTaP-IPV-HB-Hib vaccine [18].

This study was conducted in order to obtain licensure in India for the investigational formulation qualifying the switch of HepB drug substance source from SHIPL, India to Pilar, Argentina in the investigational formulation and to confirm the non-inferiority of the SHIPL pertussis component in the investigational formulation versus imported pertussis component (from Sanofi site at MLE, France) in the existing formulation so as to maintain WHO pre-qualification status for the investigational formulation of the DTwP-HepB-Hib vaccine using for the first time a new assay and specific and clinically relevant immunogenicity endpoints for evaluating the responses to the pertussis antigens. The primary objectives of the study focused only on the demonstration of the non-inferiority of immune responses following a three-dose primary series of the investigational DTwP-HepB-Hib formulation versus that of the 2^nd^ generation licensed comparator DTwP-HepB-Hib formulation in terms of HepB seroprotection rate and pertussis immune responses. The secondary objectives were to describe the immunogenicity profile at baseline and 28 days after a three-dose primary series of both the vaccine formulations in terms of seroprotection rates, vaccine response rates and geometric mean concentrations (GMCs) to all antigens (D, T, HepB, Pertussis and Hib) when administered concomitantly with other age-recommended vaccines (oral rotavirus vaccine [ORV], oral poliovirus vaccine [OPV] and inactivated poliovirus vaccine [IPV]). Safety of the two vaccine formulations was also evaluated. The study was conducted in order to obtain licensure in India and to maintain WHO pre-qualification status for the investigational formulation of the DTwP-HepB-Hib vaccine aiming at replacing the previous one.

## Methods

### Study design and participants

This was a phase 3, randomized, observer-blind study conducted in 8 centers across India among 460 infants who were followed up for safety and immunogenicity for 28 days after administration of three doses of either investigational or licensed comparator DTwP-HepB-Hib formulations at 6–8, 10–12 and 14–16 weeks of age. The study was performed from December 2018 to July 2019. The study was approved by the Indian National Regulatory Authority (The Drugs Controller General of India [DCGI]) and appropriate independent ethics committees and institutional review boards prior to the start of the study. The study was registered in the Clinical Trials Registry—India (CTRI/2018/12/016692). The conduct of this study was consistent with the standards established by the Declaration of Helsinki and compliant with the ICH guidelines for good clinical practice as well as with all local and / or national regulations and directives. Written signed informed consent was obtained from the subject’s parent/ legally acceptable representative (LAR) before any study procedures were performed. The entire informed consent process was audio-visually recorded as per Indian regulations.

The study population included infants aged 6–8 weeks, at full term of pregnancy (≥ 37 weeks) and with a birth weight ≥ 2.5 kg, or medically stable prematurely born infants (born after a gestation period of 27–36 weeks), receiving the primary series of D, T, P, HepB and Hib vaccination as per the National Immunization schedule [19]. Potential participants were excluded if they were previously vaccinated against D, T, P, HepB (except the birth dose of HepB vaccine) or Hib infections with the study vaccine or another vaccine. Other exclusion criteria included known history of D, T, P, HepB, or Hib infections, known or suspected congenital or acquired immunodeficiency or receipt of immunosuppressive therapy or long-term systemic corticosteroid therapy since birth, known systemic hypersensitivity to any of the vaccine components, or history of a life-threatening reaction to the trial vaccine or a vaccine containing the same substances, and history of definite seizure disorder and receiving anticonvulsant therapy.

Eligible subjects were randomized into two groups in 1:1 ratio to receive either the investigational DTwP-HepB-Hib vaccine formulation or the comparator (licensed) DTwP-HepB-Hib vaccine formulation. Randomization was performed using the permuted block method with stratification on centers and was managed with scratchable lists, available on site, which provided the vaccine to be administered for each enrolled participant. The study was conducted in an observer-blind manner, meaning that the subject’s parents / LAR, the study investigators and study staff, except the person in charge of the vaccination, were unaware of the group allocation throughout the study period.

Each dose (0.5 mL) of both the investigational (batch number 2PLU005A18 expiring in Jan 2020) and the licensed comparator (batch number PLU015A17 expiring in Jun 2019) DTwP-HepB-Hib formulations, presented in 10-dose vials, contained diphtheria toxoid (≥ 30 IU), tetanus toxoid (≥ 60 IU), whole cell *Bordetella pertussis* (≥ 4 IU), hepatitis B surface antigen HBsAg (rDNA) (10 μg), purified capsular polysaccharide of Hib (10 μg) conjugated to 20–40 μg of tetanus toxoid (carrier protein), adsorbed on aluminium phosphate (0.625 mg) as adjuvant, with in addition thiomersal as preservative (50 μg) along with sodium chloride (4.5 mg) and the volume was made 0.5 mL with water for injection and was administered by intramuscular route into the anterolateral aspect of the thigh by an unblinded vaccinator. The subjects received three doses of either the investigational or the comparator DTwP-HepB-Hib vaccine formulations irrespective of whether they had received HepB vaccination at birth or not. Available licensed ORV, OPV and IPV vaccines were co-administered as per the standard of care.

### Immunogenicity assessment

All subjects provided a pre-vaccination (baseline) blood sample at day 0 and a post-vaccination sample 28 days after administration of the third dose (day 84). The serological primary endpoints were assessed 28 days after the third dose of the primary series for the non-inferiority analyses. HepB seroprotection status was defined as anti-HbsAg antibody concentration ≥ 10 mIU/mL. *B*. *pertussis* immune responses were defined by the GMCs, adjusted on baseline concentrations (aGMCs), for anti-pertussis toxin (PT) and anti-fimbriae 2/3 (FIM) antibodies. For the responses against *B*. *pertussis* antigens, antibodies to PT and FIM were selected as they play important roles in protection against pertussis [20, 21].

The serological secondary endpoints assessed at baseline (day 0) and 28 days after the third dose of the primary series were antibody concentrations for each valence at each time point and above the following cut-offs: anti-D antibody concentrations ≥ 0.01 IU/mL, ≥ 0.1 IU/mL and ≥ 1.0 IU/mL; anti-T antibody concentrations ≥ 0.01 IU/ mL, ≥ 0.1 IU/mL and ≥ 1.0 IU/mL; anti-HBs antibody concentrations ≥ 10 mIU/mL and ≥ 100 mIU/mL; and anti-PRP antibody concentrations ≥ 0.15 μ/mL and ≥ 1.0 μ/mL. For the pertussis antigens, vaccine responses against PT, filamentous hemagglutinin (FHA), PRN (pertactin) and FIM antigens, were defined as subjects with post-dose 3 vaccination concentrations ≥ 4 x LLOQ if their pre-vaccination concentrations were <4 x LLOQ or with post-dose 3 vaccination concentrations ≥ the pre-vaccination concentrations if their pre-vaccination concentration were ≥ 4 x LLOQ. Vaccine seroconversion status for anti-PT, anti-FHA, anti-PRN and anti-FIM antibodies was defined as subjects with a ≥ 4-fold rise in their respective PT, FHA, PRN, FIM antibody concentrations between pre-dose 1 and post-dose 3. The ratio (post dose 3 /pre-primary) of individual Ab concentration for all Abs were also assessed.

All immunological assays were carried out at one central laboratory (Global Clinical Immunology (GCI), Sanofi, Swiftwater, Pennsylvania, USA). The assays were performed on pre-dose 1 and post-dose 3 samples obtained from all the subjects. The method used for D, T, and *B*. *pertussis* responses was a multiplexed electrochemiluminescent assay (DTP-ECL) [22]. This immunoassay also known as MesoScale Discovery electrochemiluminescent immunoassay (MSD-ECL), is a multiplexed serological assay which allows for the simultaneous quantification of human IgG against six specific antigens including *Corynebacterium diphtheriae* toxoid, *Clostridium tetani* toxoid, and four *Bordetella pertussis* antigens (FHA, PT, FIM types 2 and 3, and PRN 69kD) with very good sensitivity and ability to simultaneously detect and quantify immunoglobulin G levels to the multiple antigens with a wide dynamic working range, resulting in improved sample testing quality and throughput. For pertussis antigens, results were expressed in ELISA units calibrated against the USA/CBER lots 3 and 4 reference sera. This assay has been validated and shown to be much more reliable and clinically-relevant than several *B*. *pertussis* commercial enzymatic assays in its capacity to measure *B*. *pertussis* responses following vaccination with wP vaccines [22]. Anti-HepB antibodies were measured by the well-known commercially available VITROS ECi/ECiQ Immunodiagnostic System using chemiluminescence detection technology (VITROS, Ortho Clinical Diagnostics, Johnson & Johnson, United Kingdom), and serum levels of anti-Hib PRP antibodies were determined by an *in-house* radioimmunoassay (RIA).

### Safety assessment

Subjects were observed for 30 min after vaccination to assess the occurrence of any immediate adverse events (AEs). Participants were provided with diary cards, digital thermometers, and flexible rulers to record daily body temperature as well as solicited injection site (pain, erythema, and swelling) and systemic reactions (fever ≥ 38.0°C (≥ 100.4°F), vomiting, abnormal crying, drowsiness, loss of appetite, and irritability) up to 7 days after each and any study vaccine dose, and other unsolicited AEs up to 28 days after each and any study vaccine dose. The subject’s parents / LAR were contacted by telephone 8–10 days after each vaccination to remind them to record safety information in their diary cards. Information on unsolicited AEs was collected. Investigators assessed the causal relationship of each unsolicited systemic AE/serious AE (SAE) with vaccination. Information on SAEs was collected and assessed throughout the study, from screening on day 0 until study completion. The following adverse events of special interest (AESIs) were defined based upon the prior experience with the use of marketed pentavalent combination vaccines including anaphylaxis/hypersensitivity, convulsions, including febrile convulsion, hypotonic hyporesponsive episode (HHE), and encephalopathy. These AESIs were considered as SAEs and reported to the sponsor between day 0 and until 28 days after the last vaccination.

### Statistical analysis

For HepB, non-inferiority testing was based on the use of the two-sided 95% confidence interval (CI) of the difference of proportions of subjects with an anti-HBs Ab concentration ≥ 10 mIU/mL at day 84. The 95% CI of the difference was calculated using Wilson score method without continuity correction. Hepatitis B non-inferiority was considered demonstrated if the lower limit of the 95% CI of the difference of the two proportions (investigational formulation minus licensed formulation) was greater than minus 10%.

For pertussis, non-inferiority testing was based on the use of aGMCs and their 95% CI, on both PT and FIM results. Adjusted GMCs were computed using analysis of covariance to adjust for baseline disparities and considering the correlation between pre- and post-vaccination concentration (log10-transformed), through an ANCOVA model using the pre-vaccination (day 0) concentration as a covariate for adjustment and the vaccine group. The 95% CI of ratio between vaccine groups aGMCs was calculated using normal approximation of the mean of log10-concentration. Pertussis non-inferiority was considered if the lower limits of the 95% CI of the ratio of adjusted GMCs for PT and for FIM antibodies were above 0.5 (less than a 2-fold reduction). Overall non-inferiority was concluded if both the pertussis (both PT and FIM) and Hep B non-inferiorities were demonstrated.

Descriptive statistics were produced for each secondary endpoint. The safety and immunogenicity parameters were described with their 95% CI. Immunogenicity endpoints were summarized by vaccine groups and by time-points (pre and post primary series vaccination). For descriptive safety analyses, percentages were presented with their 95% CI (Clopper-Pearson method).

The sample size was calculated based on primary study objectives, with an alpha level of 2.5% (one-sided hypotheses), a 10% non-inferiority clinical margin for the hepatitis B responses, a 2-fold decrease clinical margin for aGMC (aGMC ratio higher than 0.5) for pertussis responses (PT and FIM) and an assumption that 85% of enrolled subjects would fulfil the Per Protocol definitions in each group. Based on simulations, testing the null hypothesis on pertussis responses with a power of 91.5% (on aGMCs at Day 84), and assuming observed standard deviations of log10 (aGMCs) of 0.75 and 0.85, respectively on PT and FIM results, required a total of 195 evaluable subjects in each group. With such a sample size and an assumption of 95% of subjects with an anti-HBs Ab concentration ≥10 mIU/mL at D84, the power to demonstrate non-inferiority on HepB was 98.4% (using the Farrington and Manning method) meaning that the overall power of the trial was at least 90%. Considering a 15% of subjects non-evaluable at D84, a total of 460 subjects was included in the trial to reach the primary objective with an overall power of at least 90%.

The primary immunogenicity analyses (non-inferiority testing) were performed on the per-protocol analysis set (PPAS) and confirmed on the full analyses set (FAS). The FAS was defined as the subset of enrolled subjects who received at least 1 dose of the study vaccines. The PPAS was defined as a subset of the FAS and comprised all subjects who had received the study vaccine and complied with all protocol-specified requirements and procedures. The secondary immunogenicity descriptive analyses were also performed on PPAS and FAS. In the FAS, subjects were analyzed by the vaccine group to which they were randomized. The safety analysis was performed on the safety analysis set (SafAS). The SafAS was defined for each stage of the trial as those subjects who have received at least 1 dose of the study vaccines. Subjects were analyzed after each dose according to the vaccine they received and after any dose according to the vaccine received at the first dose.

The statistical analysis was conducted under the responsibility of Sanofi with SAS software, Version 9.4 (SAS Institute, Cary, NC, USA).

## Results

**Fig 1:**
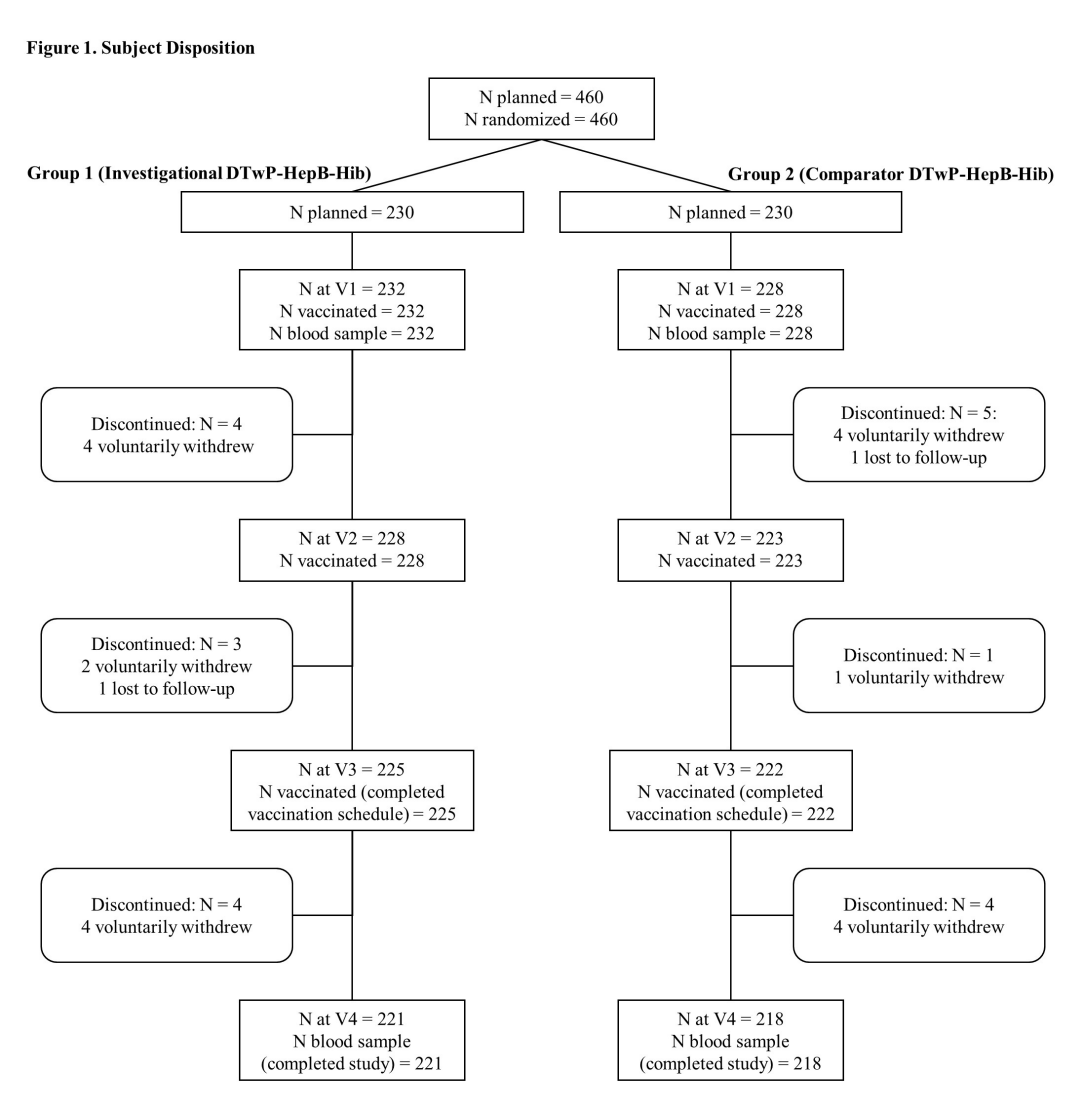
Subject disposition.

A total of 460 subjects were enrolled between December 22, 2018, and April 08, 2019, and randomized into 2 groups, group 1 (Investigational DTwP-HepB-Hib vaccine formulation, primary series, n = 232) and group 2 (Comparator (licensed) DTwP-HepB-Hib vaccine formulation, primary series, n = 228). A total of 447 subjects completed the vaccination schedule (3-dose primary series) (225 and 222 subjects in groups 1 and 2 respectively), whereas 439 patients completed the study till D84 (221 and 218 subjects in groups 1 and 2 respectively). Protocol deviations including incomplete vaccination schedule, out of window visits, and randomization errors were reported among 45 subjects (19 and 26 subjects in groups 1 and 2 respectively); (Fig 1). The FAS included all 460 subjects while PPAS included 415 subjects. The baseline demographics of the study population is showed in Table 1.

**Table 1:** Baseline demography of randomized group (FAS).

	Investigational DTwP-HepB-Hib (N = 232)	Comparator DTwP-HepB-Hib (N = 228)	All (N = 460)
**Sex, n (%)**			
** Male**	107 (46.1)	115 (50.4)	222 (48.3)
** Female**	125 (53.9)	113 (49.6)	238 (51.7)
**Male : Female ratio**	0.856	1.02	0.933
**Age at visit 01 (weeks)**			
** Mean (SD)**	6.36 (0.516)	6.34 (0.527)	6.35 (0.521)
**Weight at Birth (kg)**			
** Mean (SD)**	2.98 (0.344)	2.99 (0.356)	2.98 (0.350)

SD, standard deviation

### Immunogenicity

#### Hepatitis B seroprotection rate (primary objective)

The investigational DTwP-HepB-Hib vaccine formulation (group 1) was non-inferior to the comparator DTwP-HepB-Hib vaccine formulation (group 2) for anti HBsAg in terms of post-dose 3 seroprotection rates in the PPAS population. Seroprotection against HepB at post-dose 3 was observed in 99.1% (95% CI, 96.6–99.9) and 99.0% (95% CI, 96.5–99.9) of subjects in the groups 1 and 2 respectively. The difference in seroprotection rates between the two groups was 0.1% (95% CI, -2.47 to 2.68). The lower limit of the 2-sided 95% CI of the difference between the 2 vaccine groups was -2.4% which was well above the lower limit of the 2-sided 95% CI of the difference defined for demonstration of non-inferiority, i.e. above -10% (minus delta).

#### Pertussis immune responses (primary objective)

The investigational DTwP-HepB-Hib vaccine formulation (group 1) was non-inferior to the comparator DTwP-HepB-Hib vaccine formulation (group 2) for anti-pertussis responses in terms of post-dose 3 aGMCs in the PPAS population. The aGMC for anti-PT at day 84 were 76.7 EU/mL (95% CI, 62.1–94.7) and 63.3 EU/mL (95% CI, 50.9–78.6) for groups 1 and 2 respectively. The ratio of the aGMC for anti-PT antibodies at day 84 between the 2 study groups was 1.21 (95% CI, 0.89–1.64). The lower limit of the 95% CI of the ratio of aGMC for anti-PT (0.89) was above the lower limit defined for demonstration of non-inferiority for anti-PT, i.e. above 0.5.

The aGMC for anti-FIM at day 84 were 1079 EU/mL (95% CI, 898–1296) and 1129 EU/mL (95% CI, 935–1362) for Groups 1 and 2 respectively. The ratio of the aGMC for anti-FIM antibodies at day 84 between the 2 study groups was 0.95 (95% CI, 0.73–1.24). The lower limit of the 95% CI of the ratio of aGMC for anti-FIM (0.73) was above the lower limit defined for demonstration of non-inferiority for anti-FIM, i.e. above 0.5.

The immunogenicity of the investigational DTwP-HepB-Hib vaccine formulation was demonstrated to be non-inferior to the comparator DTwP-HepB-Hib vaccine formulation for HepB and pertussis antigens.

The results were similar in the FAS population for both hepatitis B seroprotection rate and pertussis immune responses.

#### Secondary objectives

The immune responses to the antigens of the five valences (D, T, P, HepB and Hib) in the investigational and comparator vaccine groups were also similar (Tables 2 and 3). Higher than 80% seroprotection / vaccine response rates were observed for all the five valences in the PPAS (Table 2) and in the FAS. Few subjects in the study did not receive HepB vaccine at birth. Anti-HBs immune responses tended to be similar irrespective of whether the subjects received HepB vaccination at birth or not (Tables 2 and 3).

**Table 2:** Summary of seroprotection and seronconversion rates—Pre-dose 1 and post-dose 3 (PPAS).

Antigen	Timepoint	Criteria	Investigational DTwP-HepB-Hib (N = 213)			Comparator DTwP-HepB-Hib (N = 202)		
			n/M	%	(95% CI)	n/M	%	(95% CI)
**Anti-D (DTP-ECL—IU/mL)**	**Pre-dose 1**	**≥ 0.01 IU/mL**	114/213	53.5	(46.6–60.4)	99/202	49.0	(41.9–56.1)
		**≥ 0.1 IU/mL**	13/213	6.1	(3.3–10.2)	19/202	9.4	(5.8–14.3)
		**≥ 1.0 IU/mL**	1/213	0.5	(0.0–2.6)	1/202	0.5	(0.0–2.7)
	**Post-dose 3**	**≥ 0.01 IU/mL**	213/213	100.0	(98.3–100.0)	202/202	100.0	(98.2–100.0)
		**≥ 0.1 IU/mL**	213/213	100.0	(98.3–100.0)	202/202	100.0	(98.2–100.0)
		**≥ 1.0 IU/mL**	191/213	89.7	(84.8–93.4)	184/202	91.1	(86.3–94.6)
**Anti-T (DTP-ECL—IU/mL)**	**Pre-dose 1**	**≥ 0.01 IU/mL**	213/213	100.0	(98.3–100.0)	202/202	100.0	(98.2–100.0)
		**≥ 0.1 IU/mL**	210/213	98.6	(95.9–99.7)	202/202	100.0	(98.2–100.0)
		**≥ 1.0 IU/mL**	176/213	82.6	(76.9–87.5)	167/202	82.7	(76.7–87.6)
	**Post-dose 3**	**≥ 0.01 IU/mL**	213/213	100.0	(98.3–100.0)	202/202	100.0	(98.2–100.0)
		**≥ 0.1 IU/mL**	213/213	100.0	(98.3–100.0)	202/202	100.0	(98.2–100.0)
		**≥ 1.0 IU/mL**	198/213	93.0	(88.7–96.0)	184/202	91.1	(86.3–94.6)
**Anti-PT (DTP-ECL–EU/mL)**	**Pre-dose 1**	**≥ 2 EU/mL**	129/213	60.6	(53.7–67.2)	124/202	61.4	(54.3–68.1)
	**Post-dose 3**	**≥ 2 EU/mL**	203/213	95.3	(91.5–97.7)	193/202	95.5	(91.7–97.9)
	**Post-dose 3 / pre**	**≥ 4-fold rise**	159/213	74.6	(68.3–80.3)	147/202	72.8	(66.1–78.8)
		Vaccine response*	179/213	84.0	(78.4–88.7)	169/202	83.7	(77.8–88.5)
**Anti-FIM (DTP-ECL–EU/mL)**	**Pre-dose 1**	**≥ 2 EU/mL**	166/213	77.9	(71.8–83.3)	138/202	68.3	(61.4–74.7)
	**Post-dose 3**	**≥ 2 EU/mL**	210/213	98.6	(95.9–99.7)	202/202	100.0	(98.2–100.0)
	**Post-dose 3 / pre**	**≥ 4-fold rise**	200/213	93.9	(89.8–96.7)	193/202	95.5	(91.7–97.9)
		Vaccine response*	204/213	95.8	(92.1–98.0)	199/202	98.5	(95.7–99.7)
**Anti-PRN (DTP-ECL–EU/mL)**	**Pre-dose 1**	**≥ 2 EU/mL**	57/213	26.8	(20.9–33.2)	55/202	27.2	(21.2–33.9)
	**Post-dose 3**	**≥ 2 EU/mL**	211/213	99.1	(96.6–99.9)	202/202	100.0	(98.2–100.0)
	**Post-dose 3 / pre**	**≥ 4- fold rise**	193/213	90.6	(85.9–94.2)	185/202	91.6	(86.9–95.0)
		Vaccine response*	206/213	96.7	(93.3–98.7)	190/202	94.1	(89.9–96.9)
**Anti-FHA (DTP-ECL–EU/mL)**	**Pre-dose 1**	**≥ 2 EU/mL**	196/213	92.0	(87.5–95.3)	186/202	92.1	(87.5–95.4)
	**Post-dose 3**	**≥ 2 EU/mL**	213/213	100.0	(98.3–100.0)	202/202	100.0	(98.2–100.0)
	**Post-dose 3 / pre**	**≥ 4-fold rise**	119/213	55.9	(48.9–62.6)	107/202	53.0	(45.8–60.0)
		Vaccine response*	175/213	82.2	(76.3–87.1)	165/202	81.7	(75.6–86.8)
**Anti-PRP (RIA—μg/mL)**	**Pre-dose 1**	**≥ 0.15 μg/mL**	89/213	41.8	(35.1–48.7)	96/202	47.5	(40.5–54.7)
		**≥ 1 μg/mL**	22/213	10.3	(6.6–15.2)	15/202	7.4	(4.2–12.0)
	**Post-dose 3**	**≥ 0.15 μg/mL**	213/213	100.0	(98.3–100.0)	201/202	99.5	(97.3–100.0)
		**≥ 1 μg/mL**	208/213	97.7	(94.6–99.2)	197/202	97.5	(94.3–99.2)
**Anti-HBs (ELISA—mIU/mL) All subjects**	**Pre-dose 1**	**≥ 10 mIU/mL**	33/212	15.6	(11.0–21.2)	32/202	15.8	(11.1–21.6)
		**≥ 100 mIU/mL**	12/212	5.7	(3.0–9.7)	14/202	6.9	(3.8–11.4)
	**Post-dose 3**	**≥ 10 mIU/mL**	211/213	99.1	(96.6–99.9)	200/202	99.0	(96.5–99.9)
		**≥ 100 mIU/mL**	200/213	93.9	(89.8–96.7)	193/202	95.5	(91.7–97.9)
** Anti-HBs (ELISA—mIU/mL) in subjects with HepB birth dose (FAS)**	**Pre-dose 1**	**≥ 10 mIU/mL**	34/217	15.7	(11.1–21.2)	35/209	16.7	(12.0; 22.5)
		**≥ 100 mIU/mL**	14/217	6.5	(3.6–10.6)	16/209	7.7	(4.4; 12.1)
	**Post-dose 3**	**≥ 10 mIU/mL**	204/206	99.0	(96.5; 99.9)	196/199	98.5	(95.7; 99.7)
		**≥ 100 mIU/mL**	194/206	94.2	(90.0; 97.0)	190/199	95.5	(91.6; 97.9)
** Anti-HBs (ELISA—mIU/mL) in subjects without HepB birth dose (FAS)**	**Pre-dose 1**	**≥ 10 mIU/mL**	4/14	28.6	(8.4; 58.1)	0/19	0.0	(0.0; 17.6)
		**≥ 100 mIU/mL**	2/14	14.3	(1.8; 42.8)	0/19	0.0	(0.0; 17.6)
	**Post-dose 3**	**≥ 10 mIU/mL**	14/14	100.0	(76.8; 100.0)	19/19	100.0	(82.4; 100.0)
		**≥ 100 mIU/mL**	13/14	92.9	(66.1; 99.8)	18/19	94.7	(74.0; 99.9)

n refers to the number of subjects experiencing the endpoint listed in the first three columns; M refers to the number of subjects with available data for the relevant endpoint; *Vaccine response is defined as: If the pre-primary vaccination concentration is < 4 x LLOQ, then the post-primary vaccination concentration is ≥ 4 x LLOQ; If the pre-primary vaccination concentration is ≥ 4 x LLOQ, then the post-primary vaccination concentration is ≥ the pre-primary concentration; LLOQ is 2 EU/mL for pertussis antigens for DTP-ECL assay.

**Table 3:** Summary of geometric means—Pre-dose 1 and post-dose 3 (PPAS).

Antigen	Timepoint	Investigational DTwP-HepB-Hib (N = 213)			Comparator DTwP-HepB-Hib (N = 202)		
		M	GM	(95% CI)	M	GM	(95% CI)
**Anti-D (DTP-ECL—IU/mL)**	**Pre-dose 1 (V01)**	213	0.012	(0.010–0.014)	202	0.012	(0.010–0.015)
	**Post-dose 3 (V04)**	213	3.51	(3.09–3.99)	202	3.37	(3.00–3.78)
	**Ratio V04/V01**	213	303	(235–389)	202	271	(209–352)
**Anti-T (DTP-ECL—IU/mL)**	**Pre-dose 1 (V01)**	213	2.16	(1.89–2.47)	202	2.28	(2.02–2.57)
	**Post-dose 3 (V04)**	213	3.44	(3.05–3.87)	202	3.69	(3.23–4.20)
	**Ratio V04/V01**	213	1.59	(1.31–1.92)	202	1.62	(1.36–1.94)
**Anti-PT (DTP-ECL–EU/mL)**	**Pre-dose 1 (V01)**	213	3.36	(2.86–3.95)	202	3.38	(2.86–3.99)
	**Post-dose 3 (V04)**	213	76.8	(61.0–96.7)	202	63.2	(50.1–79.6)
	**Ratio V04/V01**	213	22.9	(16.5–31.7)	202	18.7	(13.4–26.1)
**Anti-FIM (DTP-ECL–EU/mL)**	**Pre-dose 1 (V01)**	213	7.41	(6.10–9.00)	202	5.54	(4.52–6.80)
	**Post-dose 3 (V04)**	213	1041	(841–1288)	202	1172	(993–1383)
	**Ratio V04/V01**	213	140	(102–193)	202	211	(157–286)
**Anti-PRN (DTP-ECL–EU/mL)**	**Pre-dose 1 (V01)**	213	1.71	(1.49–1.97)	202	1.81	(1.53–2.13)
	**Post-dose 3 (V04)**	213	64.1	(54.9–74.8)	202	64.2	(54.9–75.1)
	**Ratio V04/V01**	213	37.4	(30.4–45.9)	202	35.5	(28.3–44.6)
**Anti-FHA (DTP-ECL–EU/mL)**	**Pre-dose 1 (V01)**	213	10.4	(8.86–12.2)	202	10.9	(9.12–13.0)
	**Post-dose 3 (V04)**	213	54.5	(48.5–61.3)	202	46.1	(41.0–51.9)
	**Ratio V04/V01**	213	5.24	(4.22–6.50)	202	4.23	(3.41–5.26)
**Anti-PRP (RIA—μg/mL)**	**Pre-dose 1 (V01)**	213	0.129	(0.104–0.160)	202	0.128	(0.104–0.157)
	**Post-dose 3 (V04)**	213	15.0	(12.9–17.4)	202	12.6	(10.7–14.8)
	**Ratio V04/V01**	213	116	(88.9–151)	202	98.4	(76.5–127)
**Anti-HBs (ELISA—mIU/mL) All subjects**	**Pre-dose 1 (V01)**	212	4.43	(3.71–5.29)	202	4.68	(3.80–5.75)
	**Post-dose 3 (V04)**	213	1035	(856–1250)	202	1019	(844–1231)
	**Ratio V04/V01**	212	232	(176–306)	202	218	(165–288)
**Anti-HBs (ELISA—mIU/mL) in subjects with HepB birth dose (FAS)**	**Pre-dose 1 (V01)**	217	4.62	(3.82; 5.59)	209	4.8	(3.90; 5.90)
	**Post-dose 3 (V04)**	206	1053	(872; 1272)	199	1036	(847; 1267)
	**Ratio V04/V01**	218	238	(181; 313)	209	211	(158; 283)
**Anti-HBs (ELISA—mIU/mL) in subjects without HepB birth dose (FAS)**	**Pre-dose 1 (V01)**	14	6.59	(2.59; 16.8)	19	2.78	(2.38; 3.24)
	**Post-dose 3 (V04)**	14	959	(359; 2562)	19	802	(504; 1276)
	**Ratio V04/V01**	14	146	(28.3; 750)	19	288	(186; 448)

M refers to the number of subjects with available data for the relevant endpoint.

### Safety

**Fig 2:**
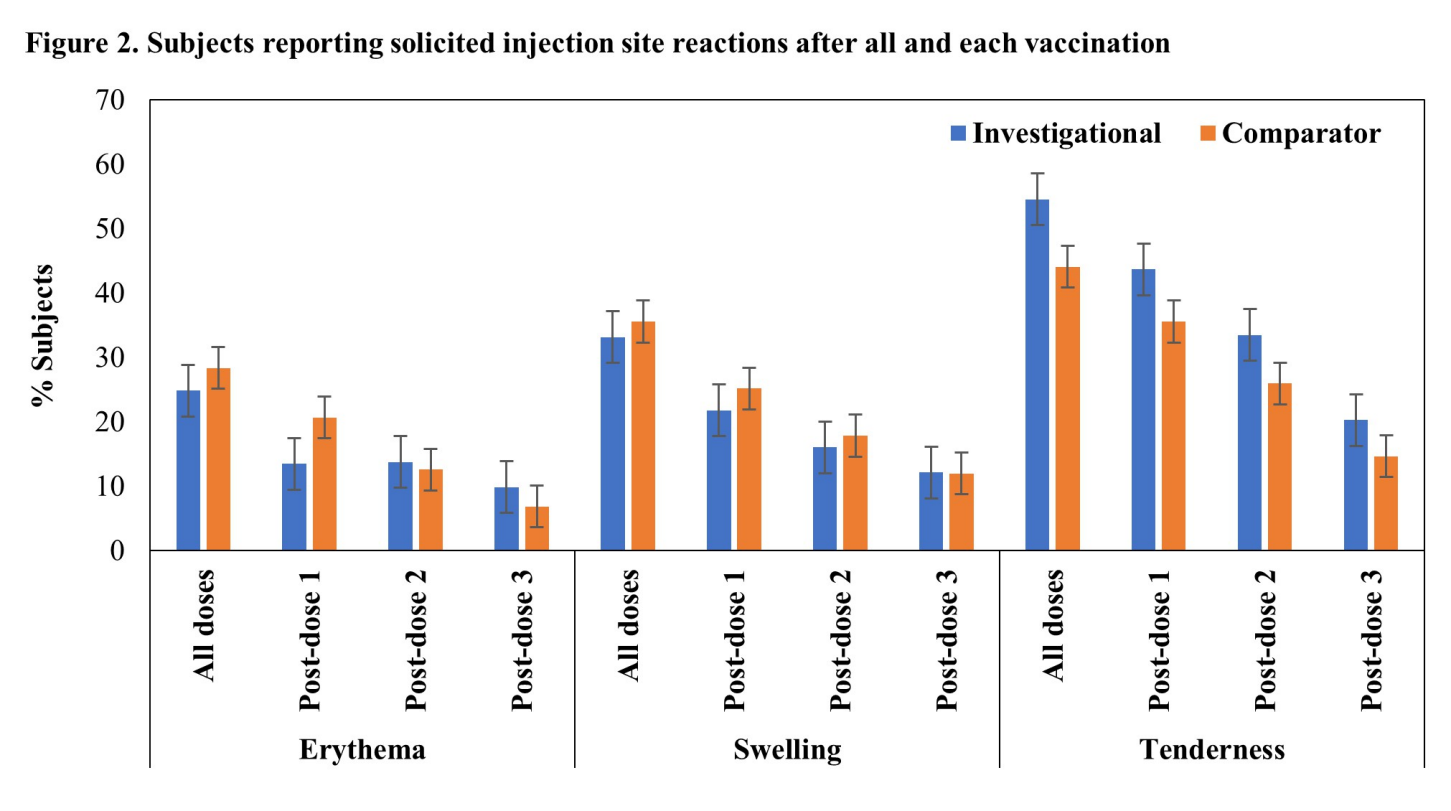
Subjects reporting solicited injection site reactions after all and each vaccination.

**Fig 3:**
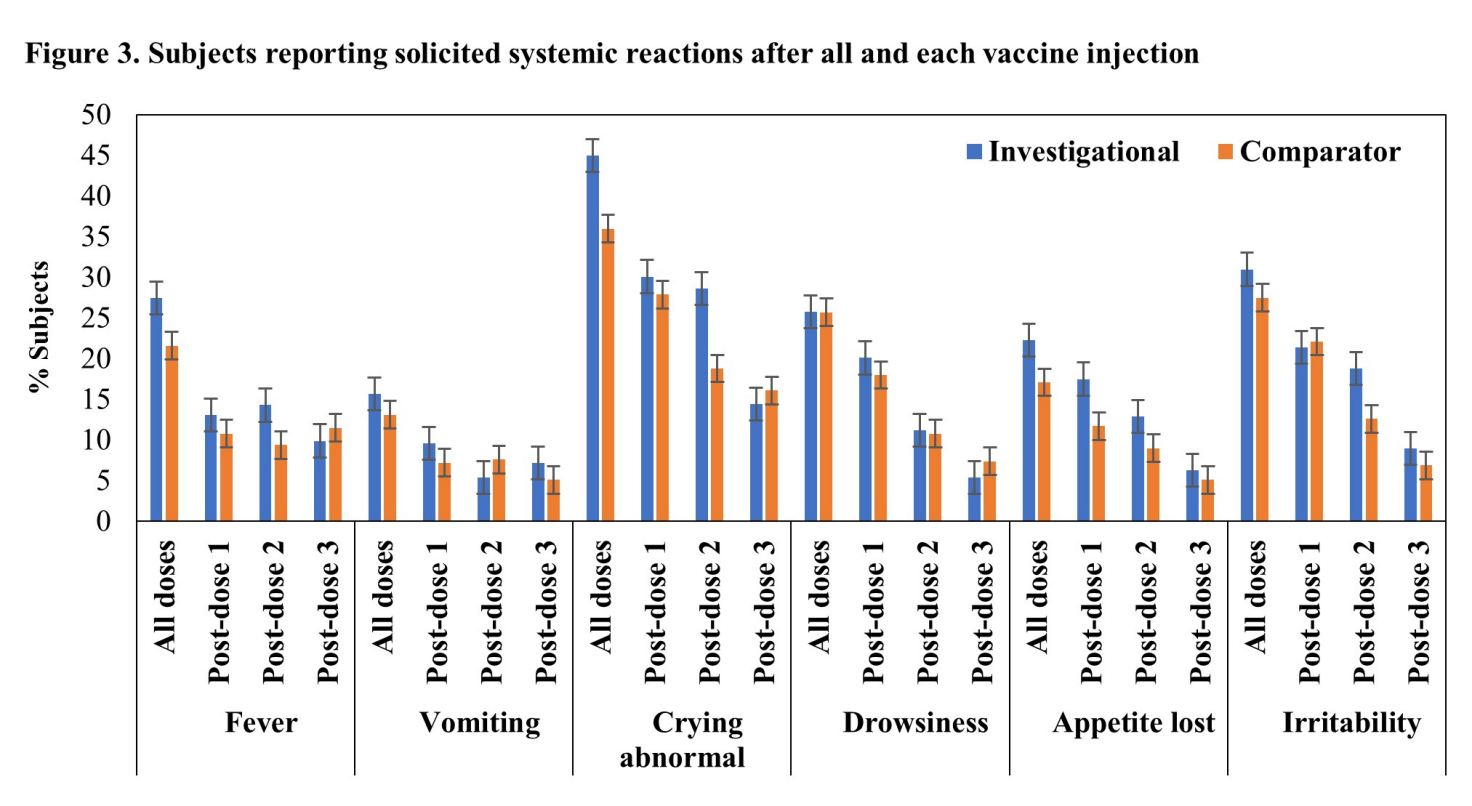
Subjects reporting solicited systemic reactions after all and each vaccine injection.

There were no AEs leading to study discontinuation, no SAEs and no AESI reported in the study. No immediate AEs were reported after any vaccination in both investigational and comparator vaccine groups. Solicited reactions (injection site reactions and systemic reactions) within 7 days after any vaccination were reported in 71.6% (164/229) and 66.7% (148/222) of subjects of investigational and comparator vaccine groups respectively (Figs 2 and 3). Most of the reactions were of grade 1 or grade 2 intensity. Few subjects reported reactions of grade 3 intensity (3.5% and 3.2% subjects in investigational and comparator vaccine groups respectively).

Unsolicited AEs within 28 days after any vaccine injection were reported in 4.7% (11/233) and 4.0% (9/227) of subjects of investigational and comparator vaccine groups respectively (Table 4). None of the unsolicited AEs were considered related to the vaccine administered. None of the subjects reported any unsolicited AEs of grade 3 intensity in both investigational and comparator vaccine groups.

**Table 4:** Unsolicited AEs within 28 days after any vaccine injections, by system organ class and preferred term (safety analysis set).

Subjects experiencing at least one	Investigational DTwP-HepB-Hib				Comparator DTwP-HepB-Hib			
	(N = 233)				(N = 227)			
	n	%	(95% CI)	AEs, n	n	%	(95% CI)	AEs, n	
**Unsolicited AE**	11	4.7	(2.4–8.3)	13	9	4.0	(1.8–7.4)	13	
**Ear and labyrinth disorders**	1	0.4	(0.0–2.4)	1	0	0.0	(0.0–1.6)	0	
** Ear pain**	1	0.4	(0.0–2.4)	1	0	0.0	(0.0–1.6)	0	
**Gastrointestinal disorders**	1	0.4	(0.0–2.4)	1	4	1.8	(0.5–4.5)	7	
** Anal fissure**	0	0.0	(0.0–1.6)	0	1	0.4	(0.0–2.4)	1	
** Diarrhoea**	1	0.4	(0.0–2.4)	1	4	1.8	(0.5–4.5)	4	
** Vomiting**	0	0.0	(0.0–1.6)	0	2	0.9	(0.1–3.1)	2	
**General disorders and administration site conditions**	4	1.7	(0.5–4.3)	4	1	0.4	(0.0–2.4)	1	
** Pyrexia**	4	1.7	(0.5–4.3)	4	1	0.4	(0.0–2.4)	1	
**Infections and infestations**	7	3.0	(1.2–6.1)	7	4	1.8	(0.5–4.5)	4	
** Abscess**	0	0.0	(0.0–1.6)	0	1	0.4	(0.0–2.4)	1	
** Acarodermatitis**	1	0.4	(0.0–2.4)	1	0	0.0	(0.0–1.6)	0	
** Conjunctivitis**	1	0.4	(0.0–2.4)	1	1	0.4	(0.0–2.4)	1	
** Nasopharyngitis**	4	1.7	(0.5–4.3)	4	2	0.9	(0.1–3.1)	2	
** Respiratory tract infection**	1	0.4	(0.0–2.4)	1	0	0.0	(0.0–1.6)	0	
**Respiratory, thoracic and mediastinal disorders**	0	0.0	(0.0–1.6)	0	1	0.4	(0.0–2.4)	1	
** Cough**	0	0.0	(0.0–1.6)	0	1	0.4	(0.0–2.4)	1	

n refers number of subjects experiencing the endpoint listed in the first column.

## Discussion

This phase 3 study conducted in India evaluated the safety and immunogenicity in 460 infants followed up for 28 days after administration of three doses of either investigational or existing vaccine formulations of the SHIPL DTwP-HepB-Hib pentavalent vaccine at 6–8 weeks, 10–12 weeks and 14–16 weeks of age when administered concomitantly with other age-recommended vaccines.

Both primary objectives were met in the study. The overall non-inferiority was demonstrated because the statistical tests concluded to the non-inferiority of the investigational DTwP-HepB-Hib formulation versus the comparator DTwP-HepB-Hib formulation at day 28 post-dose 3 for immune responses to both HepB and pertussis (PT and FIM) antigens contained in the vaccines. The immune responses to antigens of the five valences (D, T, HepB, Pertussis and Hib) at day 28 post-dose 3 were also observed to be similar in the investigational and comparator DTwP-HepB-Hib groups.

Pre-immunization (day 0) anti-T seroprotection rates (i.e. ≥ 0.01 IU/mL) were observed to be 100% of subjects in both the groups. This was due to the presence of maternal antibodies and points to the highly successful maternal immunization program in India by which elimination of maternal and neonatal tetanus was achieved in 2015 [23]. At day 84, seroprotection for anti-T (≥ 0.01 IU/mL) and anti-T antibody concentration ≥ 0.1 IU/mL which is established immunological correlate for long-term protection [24] were observed in 100% of subjects in both the groups.

The anti-HBs seroprotective rates tended to be similar irrespective of whether the study subjects received HepB vaccination at birth or not. The results are similar to those observed in other HepB containing combination vaccine studies [25–27].

The demonstration of the non-inferiority of the investigational DTwP-HepB-Hib vaccine formulation versus the licensed comparator DTwP-HepB-Hib vaccine formulation for their immune responses against *Bordetella* pertussis PT and FIM antigens and the high vaccine responses against pertussis antigens observed in the current study confirms the good immunogenicity of the investigational DTwP-HepB-Hib vaccine formulation against pertussis. The assessment of pertussis immune responses is challenging as there is no established immunological (serological) correlate(s) of protection for pertussis vaccines. It is well described that PT is the main driver of the pathogenicity of an active *B*. *pertussis* infection and that anti-PT antibodies are the main driver of the capacity of vaccinees to limit clinical symptoms, when colonized [28, 29]. Anti-PT responses are recommended to be documented as per WHO technical recommendations for wP vaccines [30]. In addition, *B*. *pertussis* agglutination assay has been the gold standard assay to assess wP vaccines responses in the past, and it has been described that most of the *B*. *pertussis* agglutinating activities measured in sera of wP-vaccinated subjects are mediated by anti-FIM antibodies [31–34]. Based on this, WHO recommends that wP vaccines must contain FIM2 and FIM3 antigens [30]. Therefore we considered that measurement of both PT and FIM antibodies would be much more relevant to assess the immunological performance of this wP-containing vaccines than relying on commercial assays not designed for this purpose and shown to have poor performances. The DTP-ECL assay used in the study is a quantitative assay. It was standardized and fully validated to accurately measure antibodies to those specific *Bordetella pertussis* antigens [22]. The DTP-ECL assay is thought to be relevant compared to commercial single-point diagnosis immunoassays that are not very well calibrated and qualified for pertussis vaccine-induced antibodies determinations [35–37] and that are frequently used in studies assessing wP vaccines. As the pertussis antigens used to formulate the DTwP-HepB-Hib vaccine formulations used in the trial is virtually identical to that used for decades by Sanofi in its wP vaccines family that have been used in France and many countries, it is therefore appropriate to rely on the performance of the Sanofi DTwP-containing vaccines measured during a clinical endpoint pertussis efficacy trial [38] and also observed in real world conditions [8, 39] to predict the pertussis protection that could be expected with this vaccine.

Vaccination with either investigational or licensed comparator DTwP-HepB-Hib vaccine formulation was found to be well tolerated. The safety profile was found to be similar in both the investigational and comparator DTwP-HepB-Hib vaccine groups. There were no safety concerns observed during this study. As seen from previous studies, safety profile of investigational vaccine in the current study was not different from the safety profile of other pentavalent vaccines when evaluated in Indian infants [40–43].

Limitation of this study were that it was conducted only in India, where Hep B vaccine is recommended at birth, so very few datapoints could be generated without a Hep B birth dose. The non-inferiority was assessed in the absence of demonstrated correlates of protection for some antigens, and therefore based on the data distribution.

Our study highlights the importance of international collaboration for the development of multivalent vaccines. For our investigational pentavalent vaccine, 3 components (diphtheria, tetanus and HiB) are manufactured in India using standard technology; the hepB component was sourced from the manufacturing facility in Argentina, while the wP component was manufactured in India after successful technology transfer including the seed strains. This suggests that for the purpose of investigational vaccine development countries need not be limited by their geographical boundaries.

Our results also add to the knowledge of evaluating and comparing immune responses to pertussis components in clinical trial settings, especially for wP containing combination vaccines. In the absence of well-defined correlates of serological protection and standardized assays or methods, the results from this study will provide guidance to researchers.

## Conclusions

The study demonstrated that the investigational DTwP-HepB-Hib vaccine formulation was non-inferior to the licensed comparator DTwP-HepB-Hib vaccine formulation both in terms of HepB seroprotection rate and in terms of pertussis immune responses. The immune responses to all the antigens (D, T, P, HepB and Hib) in both the DTwP-HepB-Hib vaccine formulations were also similar.

Overall, vaccination with either the investigational or the licensed comparator DTwP-HepB-Hib vaccine formulation was found to be well tolerated among the infants administered the 3-dose vaccine primary series (at 6–8 weeks, 10–12 weeks and 14–16 weeks of age) along with other age-recommended vaccines.

## Supporting information

S1 Checklist CONSORT 2010 checklist of information to include when reporting a randomised trial*. (DOC)

S1 File (PDF)

## Abbreviations

Ab: antibody; AE, adverse event
AESI: AEs of special interest
aGMCs: adjusted geometric mean concentrations
CI: confidence interval
D: diphtheria
DCGI: Drugs Controller General of India
DTP-ECL: DTP immunoassay by multiplexed Electro Chemiluminescent method
FAS: full analyses set; FHA, filamentous hemagglutinin
FIM: fimbriae
GMCs: geometric mean concentrations
GMCR: geometric mean concentration ratio
HepB: hepatitis B
HHE: hypotonic hyporesponsive episode
Hib: *Hemophilus influenza type B*
IPV: inactivated poliovirus vaccine
LAR: legally acceptable representative
LLOQ: lower limit of quantification
MLE: Marcy l’Etoile
MSD-ECL: MesoScale Discovery ElectroChemiLuminescence immunoassay
OPV: oral poliovirus vaccine
ORV: oral rotavirus vaccine
PPAS: per-protocol analysis set
PRN: pertactin
PT: pertussis toxin
RIA: radioimmunoassay
rHBsAg: recombinant Hepatitis B surface Antigen
SAE: serious AE; SafAS, safety analyses set
T: tetanus
WHO: World Health Organization
WHO PQ: WHO Pre-Qualification
wP: whole-cell pertussis

## References

[1] Decker MD, Edwards KM, Howe BJ. Combination Vaccines. In: Plotkin SA, Orenstein W, Offit PA, Edwards KM, editors. Vaccines. 7th ed. Elsevier Saunders; 2018:198–227.e13.

[2] Skibinski DA, Baudner BC, Singh M, O’Hagan DT. Combination vaccines. J Glob Infect Dis. 2011;3(1):63–72. doi: 10.4103/0974-777X.77298 21572611 PMC3068581

[3] Rao R, Dhingra MS, Bavdekar S, Behera N, Daga SR, Dutta AK, et al. A comparison of immunogenicity and safety of indigenously developed liquid (DTwPHB-Hib) pentavalent combination vaccine (Shan 5) with Easyfive (Liq) and Tritanrix + Hiberix (Lyo) in Indian infants administered according to the EPI schedule. Hum Vaccin. 2009;5:425–429. 19333002 10.4161/hv.5.6.7816

[4] Gandhi DJ, Dhaded SM, Ravi MD, Dubey AP, Kundu R, Lalwani SK, et al. Safety, immune lot-to-lot consistency and non-inferiority of a fully liquid pentavalent DTwP-HepB-Hib vaccine in healthy Indian toddlers and infants. Hum Vaccin Immunother. 2016;12:946–954. doi: 10.1080/21645515.2015.1100779 26580093 PMC4962968

[5] World Health Organization. SHAN5 vaccine delisted from WHO prequalification. [cited 2012 April 11]. http://www.who.int/immunization_standards/vaccine_quality/shan5_who_statement_27july10.pdf.

[6] Shantha will provide up to 37 million doses of Shan5™—Shan5™ pentavalent pediatric vaccine will protect children against 5 pediatric diseases. Sanofi Pasteur PRESS RELEASE. 2015 March 17 2015 [cited 2020 Sep 11]. https://www.sec.gov/Archives/edgar/data/1121404/000110465915020642/a15-7112_1ex99d1.htm

[7] World Health Organization. Immunization standards. Diphtheria-Tetanus-Pertussis (whole cell)-Hepatitis B-Haemophilus influenzae type b (10 dose vial). [cited 2020 Sep 9]. https://www.who.int/immunization_standards/vaccine_quality/PQ_164_Shan5_10dose/en/.

[8] Njamkepo E, Rimlinger F, Thiberge S, Guiso N. Thirty-five years’ experience with the whole-cell pertussis vaccine in France: vaccine strains analysis and immunogenicity. Vaccine. 2002;20 (9–10): 1290–4. doi: 10.1016/s0264-410x(01)00479-0 11818147

[9] Cereghino JL, Cregg JM, Heterologous protein expression in the methylotrophic yeast *Pichia pastoris*. FEMS Microbiology Reviews. 2000;24(1): 45–66. ISSN 0168-6445. 10.1016/S0168-6445(99)00029-7. 10640598

[10] Gellissen G, Janowicz ZA, Weydemann U, Melber K, Strasser AW, Hollenberg CP. High-level expression of foreign genes in *Hansenula polymorpha*. Biotechnol Adv. 1992;10(2): 179–189. 14544533 10.1016/0734-9750(92)90002-q

[11] Diminsky D, Schirmbeck R, Reimann J, Barenholz Y. Comparison between hepatitis B surface antigen (HBsAg) particles derived from mammalian cells (CHO) and yeast cells (*Hansenula polymorpha*): composition, structure and immunogenicity. Vaccine. 1997;15: 637–647. 9178464 10.1016/s0264-410x(96)00239-3

[12] Greiner VJ, Egelé C, Oncul S, Ronzon F, Manin C, Klymchenko A, et al. Characterization of the lipid and protein organization in HBsAg viral particles by steady-state and time-resolved fluorescence spectroscopy. Biochimie. 2010;92: 994–1002. doi: 10.1016/j.biochi.2010.04.014 20420879

[13] Greiner VJ, Manin C, Larquet E, Ikhelef N, Gréco F, Naville S, et al. Characterization of the structural modifications accompanying the loss of HBsAg particle immunogenicity. Vaccine. 2014;32: 1049–1054. doi: 10.1016/j.vaccine.2014.01.012 24440114

[14] Greiner VJ, Ronzon F, Larquet E, Desbat B, Estèves C, Bonvin J, et al. The structure of HBsAg particles is not modified upon their adsorption on aluminium hydroxide gel. Vaccine. 2012;30:5240–5245. doi: 10.1016/j.vaccine.2012.05.082 22705175

[15] Grélard A, Guichard P, Bonnafous P, Marco S, Lambert O, Manin C, et al. Hepatitis B subvirus particles display both a fluid bilayer membrane and a strong resistance to freeze drying: a study by solid‐state NMR, light scattering, and cryo‐electron microscopy/tomography. The FASEB Journal. 2013;27: 4316–4326. doi: 10.1096/fj.13-232843 23839934

[16] Milhiet PE, Dosset P, Godefroy C, Le Grimellec C, Guigner JM, Larquet E, et al. Nanoscale topography of hepatitis B antigen particles by atomic force microscopy. Biochimie. 2011;93: 254–259. doi: 10.1016/j.biochi.2010.09.018 20887766

[17] Tregnaghi MW, Voelker R, Santos-Lima E, Zambrano B. Immunogenicity and safety of a novel yeast *Hansenula polymorpha*-derived recombinant Hepatitis B candidate vaccine in healthy adolescents and adults aged 10–45 years. Vaccine. 2010;28: 3595–3601. 20189492 10.1016/j.vaccine.2010.02.049

[18] Syed YY. DTaP-IPV-HepB-Hib Vaccine (Hexyon®): An Updated Review of its Use in Primary and Booster Vaccination. Pediatr Drugs. 2019;21: 397–408. 10.1007/s40272-019-00353-7 PMC6794236 31444785

[19] Universal Immunisation Programme. National Health Portal (NHP) India. [cited 2020 Sep 23]. https://www.nhp.gov.in/universal-immunisation-programme_pg.

[20] Cherry JD, Gornbein J, Heininger U, Stehr K. A search for serologic correlates of immunity to *Bordetella pertussis* cough illnesses. Vaccine. 1998; 16: 1901–1906. 9796041 10.1016/s0264-410x(98)00226-6

[21] Storsaeter J, Hallander HO, Gustafsson L, Olin P. Levels of anti-pertussis antibodies related to protection after household exposure to *Bordetella pertussis*. Vaccine. 1998; 16: 1907–1916. 9796042 10.1016/s0264-410x(98)00227-8

[22] Varghese K., Bartlett W, Zheng L, Bookhout S, Vincent D, Huleatt J, et al. A New Electrochemiluminescence-Based Multiplex Assay for the Assessment of Human Antibody Responses to *Bordetella pertussis* Vaccines. Infect Dis Ther. 2021;10:2539–2561. 10.1007/s40121-021-00530-7 34476771 PMC8412870

[23] Annadurai K, Danasekaran R, Mani G. Elimination of Maternal and Neonatal Tetanus in India: A Triumph Tale. Int J Prev Med. 2017;8:15. doi: 10.4103/ijpvm.IJPVM_392_15 28348725 PMC5353766

[24] The immunological basis for immunisation series, Module 3: Tetanus. Update 2018. https://apps.who.int/iris/bitstream/handle/10665/275340/9789241513616-eng.pdf?ua=1

[25] Gatchalian S, Reyes M, Bernal N, Lefevre I, David MP, Han HH, et al. A new DTPw-HBV/Hib vaccine is immunogenic and safe when administered according to the EPI (Expanded Programme for Immunization) schedule and following hepatitis B vaccination at birth. Hum Vaccin. 2005;5: 198–203. 10.4161/hv.1.5.2163 17012860

[26] Greenberg DP, Wong VK, Partridge S, Howe BJ, Ward JI. Safety and immunogenicity of a combination diphtheria-tetanus toxoids-acellular pertussis-hepatitis B vaccine administered at two, four and six months of age compared with monovalent hepatitis B vaccine administered at birth, one month and six months of age. Pediatr Infect Dis J. 2002;21: 769–777. doi: 10.1097/00006454-200208000-00014 12192167

[27] Pichichero ME, Blatter MM, Reisinger KS, Harrison CJ, Johnson CE, Steinhoff MC, et al. Impact of a birth dose of hepatitis B vaccine on the reactogenicity and immunogenicity of diphtheria-tetanus-acellular pertussis-hepatitis B-inactivated poliovirus-*Haemophilus influenzae* type b combination vaccination. Pediatr Infect Dis J. 2002;21: 854–859. 12352809 10.1097/00006454-200209000-00014

[28] Carbonetti NH. Contribution of pertussis toxin to the pathogenesis of pertussis disease. Pathog Dis. 2015;73 (8): ftv073. doi: 10.1093/femspd/ftv073 26394801 PMC4626579

[29] Kapil P, Papin JF, Wolf RF, Zimmerman LI, Wagner L. Maternal Vaccination With a Monocomponent Pertussis Toxoid Vaccine Is Sufficient to Protect Infants in a Baboon Model of Whooping Cough. J Infect Dis. 2018;217(8): 1231–1236. doi: 10.1093/infdis/jiy022 29346585 PMC6018939

[30] World Health Organization. Recommendations for whole cell pertussis vaccines. WHO Technical Report Series No 941, 2007; Annex 6; 301–333. https://www.who.int/biologicals/publications/trs/areas/vaccines/whole_cell_pertussis/Annex%206%20whole%20cell%20pertussis.pdf

[31] Fredriksen H, Namork, Frøholm LO. Immuno-electronmicroscopy of fimbriae-like structures on *Bordetella pertussis* serotype 1.3. J Med Microbiol. 1988;25: 285–288. doi: 10.1099/00222615-25-4-285 2895814

[32] Miller J, Silverberg J, Saito TM, Humber B. An agglutinative reaction for *Hemophilus pertussis*, II: its relation to clinical immunity. J Pediatr. 1943;22: 644–651.

[33] Sako W. Studies on pertussis immunization. J Pediatr. 1947;30: 29–40. doi: 10.1016/s0022-3476(47)80280-x 20280344

[34] Langue J, Ethevenaux C, Chamosaur A, Fritzell B. Safety and Immunogenicity of *Haemophilus influenzae* type b-tetanus toxoid conjugate, presented in a dual chamber syringe with diphtheria-tetanus-pertussis and inactivated poliomyelitis combination vaccine. Eur J Pediatr. 1999;158: 7717–7722. 10.1007/s004310051186 10485302

[35] Riffelmann M, Thiel K, Schmetz J, Wirsing von Koenig CH. Performance of commercial enzyme-linked immunosorbent assays for detection of antibodies to *Bordetella pertussis*. J Clin Microbiol. 2010;48(12): 4459–4463. doi: 10.1128/JCM.01371-10 20943873 PMC3008456

[36] Pawloski LC, Plikaytis BD, Martin MD, Martin SW, Prince HE, Lapé-Nixon M, et al. Evaluation of Commercial Assays for Single-Point Diagnosis of Pertussis in the US. J Pediatric Infect Dis Soc. 2017; 6(3): e15–e21. doi: 10.1093/jpids/piw035 27451419 PMC8574169

[37] Guiso N, Berbers G, Fry NK, He Q, Riffelmann M, Wirsing von König CH, et al. What to do and what not to do in serological diagnosis of pertussis: recommendations from EU reference laboratories. Eur J Clin Microbiol Infect Dis. 2011;30: 307–312. doi: 10.1007/s10096-010-1104-y 21069406 PMC3034915

[38] Simondon F, Preziosi MP, Yam A, Kane CT, Chabirand L, Iteman I, et al. A randomized double-blind trial comparing a two-component acellular to a whole-cell pertussis vaccine in Senegal. Vaccine. 1997;15(15): 1606–1612 doi: 10.1016/s0264-410x(97)00100-x 9364690

[39] Fletcher MA, Saliou P, Ethevenaux C, Plotkin SA. The efficacy of whole cell pertussis immunisation: collected data on a vaccine produced in France. Public Health. 2001;115(2): 119–129. doi: 10.1038/sj/ph/1900745 11406777

[40] Ali SS, Chandrashekar SR, Singh M, Bansal RK, Sharma DR, Arora D. A Multicenter, Prospective, Open-Label, Non-Comparative Study to Evaluate the Immunogenicity and Tolerance of a New, Fully Liquid Pentavalent Vaccine (DTwP-HepB-Hib Vaccine). Hum Vaccin. 2007;3:116–120. doi: 10.4161/hv.3.4.4061 17617743

[41] Sharma H, Yadav S, Lalwani S, Gupta V, Kapre S, Jadhav S, et al. A phase III randomized, controlled study to assess the immunogenicity and tolerability of DTPw-HBV-Hib, a liquid pentavalent vaccine in Indian infants. Vaccine. 2011;29(13): 2359–2364. doi: 10.1016/j.vaccine.2011.01.054 21288803

[42] Sharma HJ, Yadav S, Lalwani SK, Kapre SV, Jadhav SS, Chakravarty A, et al. Immunogenicity and safety of an indigenously manufactured reconstituted pentavalent (DTwP-HBV+Hib) vaccine in comparison with a foreign competitor following primary and booster immunization in Indian children. Hum Vaccin. 2011;7(4): 451–457. doi: 10.4161/hv.7.4.14208 21403463

[43] Sharma HJ, Patil VD, Lalwani SK, Manglani MV, Ravichandran L, Kapre SV, et al. Assessment of safety and immunogenicity of two different lots of diphtheria, tetanus, pertussis, hepatitis B and *Haemophilus influenzae* type b vaccine manufactured using small and large scale manufacturing process. Vaccine. 2012;30(3): 510–516. doi: 10.1016/j.vaccine.2011.11.067 22119927

