# Supplementary material for: Safety and immunogenicity of a new formulation of a pentavalent DTwP-HepB-Hib vaccine in healthy Indian infants–A randomized study
Source: PLoS One. 2023 Aug 15;18(8):e0284898. doi: 10.1371/journal.pone.0284898 (PMC10426953; doi:10.1371/journal.pone.0284898)
Supplement: S1 File — (PDF) [file pone.0284898.s002.pdf]

Phase III Safety and Immunogenicity of an Investigational *versus* the Licensed Formulation of the Pentavalent Vaccine (DTwP-HepB-Hib) SHAN 5® when administered as Three Dose Primary Series at 6-8, 10-12 and 14-16 Weeks of Age in Healthy Indian Infants and Safety and Immunogenicity of the Investigational SHAN 5® Formulation when administered as a Single Booster Dose at 12-24 Months of Age

Multi-center, randomized, active controlled, two arm, observer blind study in 460 infants followed up for safety and immunogenicity for 28 days after administration of three doses of either investigational or existing vaccine formulation at 6-8, 10-12 and 14-16 weeks of age. Followed by single arm, open label study in the subjects upon attaining 12-24 months of age followed up for safety and immunogenicity for 28 days after administration of single booster dose of the investigational vaccine formulation.

Clinical Study Protocol

**Health Authority File Number(s):**	CTRI #: To be available later
**WHO Universal Trial Number (UTN):**	
**Study Code:**	SH504
**Development Phase:**	Phase III
**Sponsor:**	Shantha Biotechnics Pvt. Ltd. (a Sanofi company) 3rd and 4th Floor, Vasantha Chambers, Fateh Maidan Road, Basheer Bagh, Hyderabad, Telangana, India-500004
**Investigational Product(s):**	SHAN 5® (with Shantha pertussis and imported HBsAg)
**Form / Route:**	Liquid / Intramuscular
**Indication For This Study:**	SHAN 5® as three dose primary series in infants at 6-8, 10-12 and 14-16 weeks of age and as a single booster dose at 12-24 months of age
**Manufacturer:**	Shantha Biotechnics Pvt. Ltd. (a Sanofi company), Athvelli, Medchal, Ranga Reddy, Telangana, India-501401.
**Investigators**	This is a multi-center study with multiple investigators. Investigators and study sites are listed in the "List of Investigators and Centers Involved in the Trial" document.

**Sponsor's Responsible Medical Officer:**	[redacted]<br>Clinical Team Leader, Clinical R &D,<br>Shantha Biotechnics Private Limited<br>Tel: [redacted]<br>Email: [redacted]
**Clinical Team Leader**	[redacted]<br>Clinical Team Leader, Clinical R &D,<br>Shantha Biotechnics Private Limited<br>Tel: [redacted]<br>Email: [redacted]
**Clinical Program Manager**	[redacted]<br>Clinical Program Manager<br>Shantha Biotechnics Private Limited<br>Tel: [redacted]<br>Email: [redacted]
**Pharmacovigilance Global Safety Expert:**	[redacted]<br>Pharmacovigilance<br>Global Safety Expert<br>Shantha Biotechnics Private Limited<br>Tel: [redacted]<br>Email: [redacted]
**Clinical Trial Manager:**	[redacted]<br>Manager - Clinical Operations,<br>Shantha Biotechnics Private Limited<br>Tel [redacted]<br>Email: [redacted]
**Version and Date of the Protocol:**	Version 2.0 dated 10 Oct 2018

History of Protocol Versions

Version*	Date	Comments
1.0	21 June 2018	Version not approved by the IEC/IRB

Table of Contents

History of Protocol Versions 3

List of Tables 9

Synopsis 10

Table of Study Procedures – Stage 1 22

Table of Study Procedures – Stage 2 23

List of Abbreviations 24

1 Introduction 27

1.1 Background 27

1.2 Background of the Investigational Product 30

1.3 Potential Benefits and Risks 32
1.3.1 Potential Benefits to Subjects 32
1.3.2 Potential Risks to Subjects 32

1.4 Rationale for the Study 33

2 Study Objectives 33

2.1 Primary Objective(s) 33

2.2 Secondary Objective(s) 33

3 Investigators and Study Organization 34

4 Independent Ethics Committee / Institutional Review Board 35

5 Investigational Plan 36

5.1 Description of the Overall Study Design and Plan 36
5.1.1 Study Design 36
5.1.2 Justification of the Study Design 37
5.1.3 Study Plan 37
5.1.4 Visit Procedures 39
5.1.4.1 Stage 1 (Infants) 39
5.1.4.2 Stage 2 (Toddlers) 42
5.1.5 Planned Study Calendar 45
5.1.6 Periodic Safety Data Review 45

5.2 Enrollment and Retention of Study Population 45

5.2.1 Recruitment Procedures 45
5.2.2 Informed Consent Procedures 46
5.2.3 Screening Criteria 46
5.2.4 Inclusion Criteria 47
5.2.5 Exclusion Criteria 47
5.2.6 Medical History 48
5.2.7 Contraindications for Subsequent Vaccinations (Stage 1 only) 49
5.2.7.1 Temporary Contraindications 49
5.2.7.2 Definitive Contraindications 49
5.2.8 Conditions for Withdrawal 50
5.2.9 Lost to Follow-up Procedures 50
5.2.10 Classification of Subjects Who Discontinue the Study 50
5.2.11 Follow-up of Discontinuations 51
5.3 Modification of the Study and Protocol 52
5.4 Interruption of the Study 52
6 Vaccines Administered 52
6.1 Identity of the Investigational Product(s) 52
6.1.1 Identity of Study Product(s) 52
6.1.1.1 Composition 53
6.1.1.2 Preparation and Administration 53
6.1.1.3 Dose Selection and Timing 53
6.1.2 Identity of Control Product(s) 54
6.1.2.1 Composition 54
6.1.2.2 Preparation and Administration 54
6.1.2.3 Dose Selection and Timing 54
6.2 Identity of Other Product(s) 55
6.2.1 Oral poliovirus vaccine (OPV): 55
6.2.1.1 Composition 55
6.2.1.2 Preparation and Administration 55
6.2.1.3 Dose Selection and Timing 55
6.2.2 Inactivated poliovirus vaccine (IPV): 55
6.2.2.1 Composition 55
6.2.2.2 Preparation and Administration 56
6.2.2.3 Dose Selection and Timing 56
6.2.3 Oral rotavirus vaccine (ORV): 56
6.2.3.1 Composition 56
6.2.3.2 Preparation and Administration 56
6.2.3.3 Dose Selection and Timing 56
6.2.4 Measles Mumps Rubella (MMR) vaccine: 56
6.2.4.1 Composition 57

6.2.4.2 Preparation and Administration ... 57
6.2.4.3 Dose Selection and Timing ... 57
6.3 Product Logistics ... 57
6.3.1 Labeling and Packaging ... 57
6.3.2 Product Shipment, Storage, and Accountability ... 58
6.3.2.1 Product Shipment ... 58
6.3.2.2 Product Storage ... 58
6.3.2.3 Product Accountability ... 58
6.3.3 Replacement Doses ... 59
6.3.4 Disposal of Unused Products ... 59
6.3.5 Recall of Products ... 59
6.4 Blinding and Code-breaking Procedures ... 59
6.5 Randomization and Allocation Procedures ... 60
6.6 Treatment Compliance ... 61
6.7 Concomitant Medications and Other Therapies ... 61
7 Management of Samples ... 62
7.1 Sample Collection ... 62
7.2 Sample Preparation ... 62
7.3 Sample Storage and Shipment ... 63
7.4 Future Use of Stored Serum Samples for Research ... 63
8 Clinical Supplies ... 63
9 Endpoints and Assessment Methods ... 64
9.1 Primary Endpoints and Assessment Methods ... 64
9.1.1 Safety ... 64
9.1.2 Immunogenicity ... 64
9.1.2.1 Immunogenicity Endpoints ... 64
9.1.2.2 Immunogenicity Assessment Methods ... 64
9.1.3 Efficacy ... 64
9.2 Secondary Endpoints and Assessment Methods ... 65
9.2.1 Safety ... 65
9.2.1.1 Safety Definitions ... 65
9.2.1.2 Safety Endpoints ... 67
9.2.1.3 Safety Assessment Methods ... 68
9.2.1.3.1 Immediate Post-vaccination Observation Period ... 68
9.2.1.3.2 Reactogenicity (Solicited Reactions From Day 0 to Day 7 After Each Vaccination) ... 68

9.2.1.3.3 Unsolicited Adverse Events 72
9.2.1.3.4 Serious Adverse Events 73
9.2.1.3.5 Adverse Events of Special Interest 73
9.2.1.3.6 Assessment of Causality 74
9.2.2 Immunogenicity 74
9.2.2.1 Immunogenicity Endpoints 74
9.2.2.2 Immunogenicity Assessment Methods 75
9.2.3 Efficacy 76
9.3 Observational Endpoints and Assessment Methods 76
10 Reporting of Serious Adverse Events 76
10.1 Initial Reporting by the Investigator 76
10.2 Follow-up Reporting by the Investigator 77
10.3 Reporting of SAEs Occurring After a Subject Has Completed the Study 78
10.4 Assessment of Causality 78
10.5 Reporting SAEs to Health Authorities and IECs / IRBs 78
11 Data Collection and Management 78
11.1 Data Collection and CRB Completion 78
11.2 Data Management 79
11.3 Data Review 80
12 Statistical Methods and Determination of Sample Size 80
12.1 Statistical Methods 80
12.1.1 Hypotheses and Statistical Methods for Primary Objective(s) 80
12.1.1.1 Hypotheses 80
12.1.1.2 Statistical Methods 81
12.1.2 Hypotheses and Statistical Methods for Secondary Objective(s) 81
12.1.2.1 Hypotheses 81
12.1.2.2 Statistical Methods 81
12.2 Analysis Sets 82
12.2.1 Full Analysis Set 82
12.2.2 Safety Analysis Set 82
12.2.3 Per-Protocol Analysis Set 83
12.2.4 Populations Used in Analyses 83
12.3 Handling of Missing Data and Outliers 84
12.3.1 Safety 84
12.3.2 Immunogenicity 84
12.3.3 Efficacy 84

12.4 Interim / Preliminary Analysis ... 84
12.5 Determination of Sample Size and Power Calculation ... 84

13 Ethical and Legal Issues and Investigator / Sponsor Responsibilities ... 85
13.1 Ethical Conduct of the Study / Good Clinical Practice ... 85
13.2 Source Data and Source Documents ... 85
13.3 Confidentiality of Data and Access to Subject Records ... 86
13.4 Monitoring, Auditing, and Archiving ... 86
13.4.1 Monitoring ... 86
13.4.2 Audits and Inspections ... 87
13.4.3 Archiving ... 87
13.5 Financial Contract and Insurance Coverage ... 87
13.6 Stipends for Participation ... 88
13.7 Publication Policy ... 88

14 Reference List ... 89

15 Signature Page ... 92

List of Tables

Table 1: Trial Organization 34
Table 9.1: Solicited injection site reactions: terminology, definitions, and intensity scales.......... 70
Table 9.2: Solicited systemic reactions: terminology, definitions, and intensity scales 71

Synopsis

**Company:**	Shantha Biotechnics Private Limited
**Investigational Product:**	SHAN 5® (with Shantha pertussis and imported HBsAg)
**Active Substance(s):**	Diphtheria Toxoid (DT), Tetanus Toxoid (TT), whole cell Pertussis (wP), recombinant Hepatitis B surface antigen (HepB) and *Haemophilus influenzae type b* (Hib) vaccine [polyribosyl ribitol phosphate (PRP) conjugated to tetanus toxoid]

**Title of the Study:**	Safety and Immunogenicity of an Investigational *versus* the Licensed Formulation of the Pentavalent Vaccine (DTwP-HepB-Hib) SHAN 5® when administered as Three Dose Primary Series at 6-8, 10-12 and 14-16 Weeks of Age in Healthy Indian Infants and Safety and Immunogenicity of the Investigational SHAN 5® Formulation when administered as a Single Booster Dose at 12-24 Months of Age
**Development Phase:**	Phase III
**Investigators:**	This will be a multi-center study with multiple investigators.
**Study Sites:**	This will be a multi-center trial conducted at approximately 8 sites in India.<br>Investigators and sites are listed in the "List of Investigators and Centers Involved in the Trial" document.
**Planned Study Period:**	Q3 2018 to Q2 2020
**Study Design, Schedule of Study Procedures, and Methodology:**	Multi-center, randomized, active controlled, two arm, observer blind (subject's parents/legally acceptable representative (LAR), Investigator and study staff except the person in charge of the vaccination are blinded to the study vaccination) study in 460 infants followed up for safety and immunogenicity for 28 days after administration of three doses of either investigational or licensed vaccine formulation at 6-8, 10-12 and 14-16 weeks of age. Followed by single arm, open label study in the subjects upon attaining 12-24 months of age followed up for safety and immunogenicity for 28 days after administration of single booster dose of the investigational vaccine formulation.<br><br>The study will have 2 stages:<br><br><u>Stage 1:</u> Infants aged 6-8 weeks of age will be randomly allocated at 1:1 ratio, to receive 3 doses of either the investigational or the licensed SHAN 5® vaccine formulation at 6-8, 10-12 and 14-16 weeks of age, irrespective of whether they had received Hep B vaccination at birth or not. Available licensed oral rotavirus vaccine (ORV) and oral

	poliovirus vaccine / inactivated poliovirus vaccine (OPV / IPV) will be co-administered at 6-8, 10-12 and 14-16 weeks of age as per the standard of care.<br><br><u>Stage 2:</u> Toddlers aged 12-24 months who had received either the investigational or the licensed SHAN 5® vaccine formulation at 6-8, 10-12 and 14-16 weeks of age in stage 1, will receive a single booster dose of the investigational SHAN 5® vaccine formulation.<br>Subjects in stage 2 will concomitantly receive a booster dose of OPV / IPV and a 2nd dose* of Measles Mumps Rubella (MMR) vaccine at the recommended age as per the standard of care.<br>* Post completion of Stage 1 and prior to Stage 2, subjects will be provided 1st dose of MMR at the recommended age.<br><br><u>Blood sampling</u><br><u>Stage 1:</u> All subjects will provide a pre-vaccination (baseline) blood sample at Day 0 and a post-vaccination sample at Day 84 (+7 days). Approximately 5 mL of blood will be collected.<br><u>Stage 2:</u> All subjects will provide a pre-booster dose vaccination (baseline) blood sample at Day 0 and a post-vaccination sample at Day 28 (+7 days). Approximately 5 mL of blood will be collected.<br><br><u>Collection of safety data:</u><br><u>Stage 1:</u> All subjects will be observed for 30 minutes after each vaccine dose, and clinical site personnel will record any unsolicited systemic AEs occurring during that time as immediate unsolicited systemic AEs.<br>The parents/LAR of subjects will record in the Diary cards information about solicited injection site and systemic reactions for 7 days after each vaccine dose and any unsolicited AEs that may occur for 28 days after each vaccine dose.<br><br>Information on SAEs (including AESIs) will be collected throughout the trial period of Stage 1 (from Day 0 to Day 84 (+7 days)).<br><br><u>Stage 2:</u> All subjects will be observed for 30 minutes after the vaccine dose, and clinical site personnel will record any unsolicited systemic AEs occurring during that time as immediate unsolicited systemic AEs.<br>The parents/LAR of subjects will record in the Diary cards information about solicited injection site and systemic reactions from D0 to D7 post-vaccination and unsolicited AEs from D0 to D28 post-vaccination.<br><br>Information on SAEs (including AESIs) will be collected throughout the trial period of Stage 2 (from Day 0 to Day 28 (+7 days)).

**Interruption of the Study**	The study may be discontinued at any time if new data about the investigational product resulting from this study or any other studies become available to the study sponsor; or for administrative reasons; or on advice of the Sponsor, the Investigators, the ECs, or the governing regulatory authorities in India where the study is taking place.<br>If the study is prematurely terminated or suspended, the Sponsor shall promptly inform the Investigators, the ECs, the regulatory authorities, and any contract research organization(s) used in the study of the reason for termination or suspension, as specified by the applicable regulatory requirements. The Investigator shall promptly inform the subjects' parents/legally acceptable representative (LAR) and should assure appropriate subject therapy and/or follow-up.
**Primary Objective(s):**	1) To demonstrate the non-inferiority of the investigational SHAN 5® formulation *versus* the licensed SHAN 5® formulation in terms of Hep B seroprotection rate, 28 days after a three-dose primary series<br>2) To demonstrate the non-inferiority of the investigational SHAN 5® formulation *versus* the licensed SHAN 5® formulation in terms of pertussis immune responses 28 days after a three-dose primary series.
**Primary Endpoint(s):**	The following serological endpoints will be assessed 28 days after the third dose of the primary series for the non-inferiority analyses:<br>- Hep B seroprotection defined as anti-HBs Ab concentration ≥10 mIU/mL<br>- Pertussis immune responses defined by geometric mean concentrations adjusted on baseline concentrations (aGMCs) for anti-PT and anti-FIM antibodies

**Secondary Objective(s):**	Safety:<br>1) Stage 1: To describe the safety profile of investigational SHAN 5® formulation and licensed SHAN 5® formulation up to 28 days after each dose of the primary series when administered concomitantly with other age-recommended vaccines.<br>2) Stage 2: To describe the safety profile of investigational SHAN 5® formulation up to 28 days after the booster dose when administered concomitantly with other age-recommended vaccines.<br>Immunogenicity:<br>3) Stage 1: To describe the immunogenicity profile, at baseline and 28 days after a three-dose primary series of investigational SHAN 5® formulation and licensed SHAN 5® formulation in terms of seroprotection rates, seroresponse rates and GMCs to all antigens (D, T, Hep B, Pertussis and Hib) when administered concomitantly with other age-recommended vaccines.<br>4) Stage 2: To describe the persistence of antibodies against investigational SHAN 5® formulation and licensed SHAN 5® formulation at 12-24 months of age following a 3-dose primary series at 6-8, 10-12, and 14-16 weeks of age, in terms of seroprotection / seroresponse rates and GMCs to all antigens D, T, Hep B, Pertussis and Hib.<br>5) Stage 2: To describe the immunogenicity profile, 28 days after the single booster dose of investigational SHAN 5® formulation in subjects 12-24 months of age who have been primed with either investigational SHAN 5® formulation or licensed SHAN 5® formulation, in terms of seroprotection rates, seroresponse rates and GMCs to all antigens (D, T, Hep B, Pertussis and Hib) when administered concomitantly with other age-recommended vaccines
**Secondary Endpoint(s):**	Safety (for all stages):<br>1) Occurrence of any unsolicited systemic AEs reported in the 30 minutes after each and after any study vaccine dose, as applicable.<br>2) Occurrence of solicited (i.e., pre-listed in the subject's diary card [DC] and in the (electronic) case report book (CRB)), injection site reactions and systemic reactions occurring up to 7 days after each and any study vaccine dose, as applicable.<br>3) Occurrence of unsolicited (spontaneously reported) AEs up to 28 days after each and any study vaccine dose, as applicable.<br>4) Occurrence of SAEs (including AESIs), throughout the trial period of Stage 1 and Stage 2.

	Other endpoints recorded or derived as described in the statistical analysis plan. Depending on the item, these could include: nature (Medical Dictionary for Regulatory Activity [MedDRA] preferred term), time of onset, duration, number of days of occurrence, Grade of intensity, relationship to vaccine, action taken, whether the AE led to early termination from the study, seriousness, or outcome.<br><br><u>Immunogenicity (all stages):</u><br><br>The following serological endpoints will be assessed at baseline D0 and 28 days after the third dose of the primary series in stage 1 and at baseline D0 and 28 days after the booster dose in stage 2:<br><br>5) Antibody concentration above the following cut-off for each valence<br>• Anti-D antibody concentration ≥ 0.01 IU/mL, ≥ 0.1 IU/mL and ≥ 1.0 IU/mL<br>• Anti-T antibody concentration ≥ 0.01 IU/≥ 0.1 IU/mL and ≥ 1.0 IU/mL<br>• Anti-HBs antibody concentration ≥ 10 mIU/mL and ≥ 100 mIU/mL<br>• Anti-PRP antibody concentration ≥ 0.15 mcg/mL and ≥ 1.0 mcg/mL<br>6) Antibody concentration for each valence at each time point<br>7) Pertussis antigens vaccine response status for anti-PT, anti-FHA, anti-PRN and anti-FIM antibodies defined as follows: post-dose 3 vaccination concentration ≥ 4xLLOQ[¥] if the pre-vaccination concentration is <4xLLOQ or with post-Dose 3 vaccination concentration ≥ the pre-vaccination concentration if the pre-vaccination concentration is ≥4xLLOQ<br><br>[¥]according to MesoScale Discovery Immunoassay (MSD) Multiplexed Electro Chemiluminescent Method (MSD-ECL)<br><br>8) Pertussis antigens vaccine seroconversion status for anti-PT, anti-FHA, anti-PRN and anti-FIM antibodies defined as follows: a ≥ 4-fold rise in the respective PT, FHA, PRN, FIM antibody concentration between pre-Dose 1 and post-Dose 3<br>9) Ratio (post dose 3 /pre-primary; post booster /pre-booster) of individual Ab concentration for all Abs
**Planned Sample Size:**	A total of 460 subjects will be enrolled in Stage 1 and out of these all available subjects will be enrolled in Stage 2.<br><br>Subjects enrolled in Stage 1 will be randomized into 2 groups:

	Group 1 (iSHAN 5: Investigational SHAN 5® formulation, primary series): n = 230<br>Group 2 (cSHAN 5: Comparator (licensed) SHAN 5® formulation, primary series): n = 230<br><br>Stage 2: All available subjects of Group 1 and 2 who have received a full primary series during stage 1 will be enrolled in Stage 2
**Duration of Participation in the Study:**	Stage 1: The duration of each subject's active participation in the study will be approximately 84 days<br>Stage 2: The duration of each subject's active participation in the study will be approximately 28 days
**Investigational Product:**	**1. SHAN 5®** (with Shantha pertussis and imported HBsAg)
Form:	Liquid, presented in 10-dose vial
Composition:	Each 0.5 mL dose of vaccine contains:<br><u>Active ingredients:</u><br>Diphtheria Toxoid ≥ 30 IU<br>Tetanus Toxoid ≥ 60 IU<br>*B. pertussis* (Whole cell) ≥ 4 IU<br>HBsAg (rDNA) 10 μg<br>Purified capsular polysaccharide of Hib conjugated to 20-40 μg of Tetanus Toxoid (carrier protein) 10 μg<br><u>Other ingredients:</u><br>Preservative Thiomersal 0.050 mg<br>Sodium Chloride I.P 4.5 mg<br>Water for injection I.P. q.s. to 0.5 mL<br><br><u>Adjuvant:</u><br>Aluminum Phosphate Gel equivalent to Al+++ 0.625
Route:	Intramuscular injection into the anterolateral aspect of the thigh
Batch Number:	To be defined (TBD)
**Control Product:**	**2. SHAN 5®** (with imported pertussis and Shantha HepB)
Form:	Liquid, presented in 10-dose vial
Composition:	Each 0.5 mL dose of vaccine contains:<br><u>Active ingredients:</u><br>Diphtheria Toxoid ≥ 30 IU<br>Tetanus Toxoid ≥ 60 IU<br>*B. Pertussis*(Whole cell) ≥ 4 IU<br>HBsAg(rDNA) 10 μg

	Purified capsular polysaccharide of Hib conjugated to 20-40 μg of Tetanus Toxoid (carrier protein) 10 μg<br>Other ingredients:<br>Preservative Thiomersal 0.050 mg<br>Sodium Chloride I.P 4.5 mg<br>Water for injection I.P. q.s. to 0.5 mL<br><br>Adjuvant:<br>Aluminum Phosphate Gel equivalent to Al+++ 0.625
Route:<br>***Batch Number:***	Intramuscular injection into the anterolateral aspect of the thigh<br>PLU015A17 expiring in June 2019
**Other Product(s):**	Oral rotavirus vaccine (ORV): The Bharat biotech vaccine (Rotavac®) or any locally available licensed ORV will be used.
	Oral poliovirus vaccine (OPV): The Bharat biotech vaccine (Biopolio® B1/3) will be used.
	Inactivated poliovirus vaccine (IPV): The Shantha Biotechnics vaccine (SHANIPV™) will be used.
	Measles Mumps Rubella (MMR) vaccine: TRESIVAC™ (Serum Institute of India) or any locally available licensed MMR vaccine will be used.
**Inclusion Criteria:**	An individual must fulfill *all* of the following criteria to be eligible for study enrollment:<br>For Stage 1:<br>1. Infants between 6-8 weeks of age (42 to 56 days, both days inclusive) on the day of enrollment.<br>2. Healthy infants, born at full term of pregnancy (≥ 37 weeks) with a birth weight ≥ 2.5 kg or medically stable* prematurely born infants (born after a gestation period of 27-36 weeks).<br><br>* Medically stable refers to premature infants who do not require significant medical support or ongoing management for debilitating disease and who have demonstrated sustained growth curve by the time they receive the first dose of study vaccine.<br>3. Informed consent form signed by one or both parents or by the legally acceptable representative (LAR) as per local requirements.<br>4. Subjects and Parents/LAR are able to attend all scheduled visits and to comply with all study procedures.<br><br>For Stage 2:

	1. Toddlers aged between 12-24 months of age on the day of enrollment and had received either the investigational or the licensed SHAN 5® vaccine formulation at 6-8, 10-12 and 14-16 weeks of age during stage 1 of the trial.<br>2. Informed consent form signed by one or both parents or by the legally acceptable representative (LAR) as per local requirements.<br>3. Subjects and Parents/LAR are able to attend all scheduled visits and to comply with all study procedures.
**Exclusion Criteria:**	An individual fulfilling *any* of the following criteria is to be excluded from study enrollment:<br>1. Participation in another clinical trial in the 4 weeks preceding the trial inclusion or planned participation during the present trial period in another clinical trial investigating a vaccine, drug, medical device, or medical procedure.<br>2. Receipt of any vaccine in the 4 weeks preceding the first trial vaccination (except BCG, birth dose OPV and birth dose of Hep B vaccine).<br>3. Planned receipt of any other vaccine within the period from 8 days before to 8 days after each trial vaccination except OPV if not given at birth and during National Immunization Day (NID).<br>4. For Stage 1 only: Previous vaccination against the diphtheria, tetanus, pertussis, hepatitis B (except the birth dose of Hep B vaccine) or *Haemophilus influenza* type b infection with the trial vaccine or another vaccine.<br>5. For Stage 2 only: Previous booster dose vaccination against diphtheria, tetanus, pertussis, hepatitis B, or *Haemophilus influenza* type b infection with the trial vaccine or another vaccine.<br>6. Past or current receipt of immunoglobulins, blood or blood-derived products or planned administration during the trial.<br>7. Known or suspected congenital or acquired immunodeficiency; or receipt of immunosuppressive therapy, such as anti-cancer chemotherapy or radiation therapy since birth; or long-term systemic corticosteroid therapy (prednisone or equivalent for more than 2 consecutive weeks since birth).<br>8. History of diphtheria, tetanus, pertussis, hepatitis B, or *Haemophilus influenza* type b infections (confirmed either clinically, serologically or microbiologically).<br>9. Known personal or maternal history of HIV or hepatitis B seropositivity.

	10. Known systemic hypersensitivity to any of the vaccine components, or history of a life threatening reaction to the trial vaccine or a vaccine containing the same substances.<br>11. Known thrombocytopenia, as reported by the parent/ legally acceptable representative.<br>12. Bleeding disorder or receipt of anticoagulants in the 3 weeks preceding inclusion, contradicting intramuscular vaccination.<br>13. Chronic illness that, in the opinion of the investigator, is at a stage where it might interfere with trial conduct or completion. (Chronic illness may include, but is not limited to, cardiac, renal, autoimmune, hepatic, hematological, genetic disorders, atopic conditions, congenital defects, diabetes, convulsions or encephalopathy etc.).<br>14. Moderate or severe acute illness/infection (according to investigator judgment) on the day of vaccination or febrile illness (axillary temperature ≥ 100.4 °F or ≥38 °C) on the day of inclusion (a prospective subject should not be included in the study until the condition has resolved or the febrile event has subsided).<br>15. Identified as a natural or adopted child of the Investigator, relatives or employee with direct involvement in the proposed study.<br>16. Subject with definite seizure disorder and getting anticonvulsant therapy.
**Statistical Methods:**	Primary objective<br>The immunogenicity of investigational SHAN 5® will be compared to that of licensed SHAN 5® at D84, i.e. 28 days after the third vaccine injection, using non-inferiority testing on Hep B and Pertussis responses.<br><br>*Hep B non-inferiority testing*<br>For Hep B testing, the primary parameter will be the difference of the proportion of subjects with an anti-HBs Ab concentration ≥10 mIU/mL at D84 between the two study vaccine groups. The hypotheses tested will be the following:<br>• H0: $P_{iSHAN\ 5} - P_{cSHAN\ 5} \leq -10\%$<br>• H1: $P_{iSHAN\ 5} - P_{cSHAN\ 5} > -10\%$<br>With P = proportion of subjects with an anti-HBs Ab concentration ≥10 mIU/mL at D84 (%).<br>For HepB, investigational SHAN 5® will be considered as non-inferior to licensed SHAN 5® if the hypothesis H0 is rejected. The statistical methodology will be based on the use of the two-sided 95% confidence interval (CI) of the difference of proportions of subjects with an anti-HBs Ab concentration ≥10 mIU/mL at D84. The 95% CI

	for differences will be calculated using Wilson score method without continuity correction.<br><br>Non-inferiority will be demonstrated if the lower limit of the 95% CI of the difference of the two proportions $P_{iSHAN\ 5}$- $P_{cSHAN\ 5}$ is > -10%.<br><br>*Pertussis non-inferiority testing*<br><br>For Pertussis testing, the primary parameter will be the ratio of the adjusted GMC (aGMC) for anti-PT and for anti-FIM antibodies at Day 84 between the 2 study groups. The hypotheses tested will be the following:<br>• H0: aGMC $_{iSHAN\ 5}$ / aGMC $_{cSHAN\ 5}$ ≤ 1/2<br>• H1: aGMC $_{iSHAN\ 5}$ / aGMC $_{cSHAN\ 5}$ > 1/2<br><br>Non-inferiority will be demonstrated if the lower limits of the 95% CI of the ratio of adjusted GMCs for PT and for FIM antigens are > 0.5.<br><br>Adjusted GMCs will be computed using analysis of covariance to adjust for baseline disparities and consider the correlation between pre- and post- concentration, through an ANCOVA model using the pre-vaccination (D0) concentration as a covariate for adjustment in order to account for the associated variability.<br><br>Pertussis non-inferiority will be demonstrated if each of the 2 lower bounds (for PT and FIM) of two-sided 95% CIs for the ratio are above the 0.5 margin.<br>The 95% CI of ratio between aGMC will be calculated using normal approximation of the log10 of the concentration.<br><br>Overall non-inferiority will be demonstrated if the Pertussis and Hepatitis B non-inferiority are demonstrated. Non-inferiority testing will be based on the results observed using the per-protocol analysis set (PPAS) as the main analysis and also on the full analysis set in order to confirm the results observed using the PPAS.<br><br><u>Secondary objectives</u><br><br>Descriptive statistics will be produced for each secondary endpoint.<br><br>The main safety and immunogenicity parameters will be described with 95% CI.<br><br>Immunogenicity endpoints will be summarized by vaccine group and by timepoint (pre and post primary series vaccination and pre and post booster vaccination). The following parameters will be used for anti-D, anti-T, anti-Hep B, anti- Pertussis and anti-Hib antibody concentration:<br>• Percentage of subjects with concentration above predefined cut-off (as per secondary endpoints)

- Percentage of subjects with ≥4 fold rise and vaccine response in anti-pertussis antibody concentration
- Geometric mean ratio (post dose 3 /pre-primary; post booster /pre-booster) of individual Ab concentration for all Abs (GMCR)
- GMC for each valence, adjusted GMCs for PT, FIM, PRN and FHA

For descriptive immunogenicity analyses, geometric mean concentration (GMCs), adjusted GMCs (for Pertussis endpoints) and geometric mean concentration ratio (GMCR) will be calculated with their 95% CI (normal approximation method).

Immunogenicity criteria will be described for available blood samples before the first dose and one month after the third dose of the combined vaccines, and before and one month after the booster dose. Reverse Cumulative Distribution Curves (RCDCs) of individual concentration will be presented for the two groups.

For descriptive safety analyses, percentages will be presented with their 95% CI (Clopper-Pearson method). Safety data at each time point will be summarized by vaccine group.

<u>Sample size calculation</u>

A total of 460 subjects will be enrolled in the study. Subjects will be randomly allocated to one of the two groups (iSHAN 5 or cSHAN 5) using the 1:1 ratio.

The sample size is calculated based on primary study objectives, with an alpha level of 2.5% (one-sided hypotheses), a 10% non-inferiority clinical margin for the hepatitis B responses, a 2-fold ratio for aGMC ratio for pertussis responses (PT and FIM) and an assumption of 85% of enrolled subjects fulfilling the Per Protocol definitions in each group.

The sample size calculations are based on the following assumptions:

**Endpoints**	**Ref* std(log10) or seroprotection rate**	**ratio or δ for NI**	**With unilateral alpha 0.025**<br>**Power for NI**<br>**N=390**
**Pertussis : anti-PT**	log sd aGMC=0,75	2	97,7%
**Pertussis : anti-FIM**	log sd aGMC=0,85	2	93,7%

<table>
<tr>
<td></td>
<td>
<table>
<tr><td>Anti-HBs ≥ 10 mIU/ml</td><td>95%</td><td>10%</td><td>98,4%</td></tr>
<tr><td colspan="2">Overall power</td><td></td><td>90,1%</td></tr>
</table>
* reference level were observed on previous study samples results.
Based on simulations, testing the null hypothesis on Pertussis responses with a power of 91.5% (on aGMCs at Day 84) will require a total of 195 evaluable subjects in each group. With such a sample size, power to demonstrate non-inferiority on HepB, with an assumption of 95% of subjects with an anti-HBs Ab concentration ≥10 mIU/mL at D84 will be 98.4%, using the Farrington and Manning method, meaning that the overall power of the trial is at least 90%.
Considering a 15% of subjects non-evaluable at D84, a total of 460 subjects should be included in the trial to reach the primary objective with an overall power of at least 90%.
For the descriptive assessment of the booster immune response, a subset of 300 subjects will ensure from +/-5.2% to 3.4% of variability around expected seroprotection / seroconversion rates from 70% to 90%.
</td>
</tr>
</table>

Table of Study Procedures – Stage 1

Phase III Study, 4 Visits, 3 Phone calls, 3 Vaccinations, 2 Blood Samples, 84 Days' Duration per Subject

**Visit Number (V)**	**V01**	**PC1***	**V02**	**PC2***	**V03**	**PC3***	**V04**
**Trial Timelines Days**	**D0**	**D8**	**D28**	**D36**	**D56**	**D64**	**D84**
**Visit Intervals**		**V01 + 8-10D**	**V01 + 28-35 D**	**V02 + 8-10D**	**V02 + 28-35 D**	**V03 + 8-10D**	**V03 + 28-35 D**
**Indicative Age of Subject (WoA)**	**6-8**		**10-12**		**14-16**		**18-20**
Informed Consent	X						
Demography	X						
Subject Medical History & Ongoing Diseases	X						
Subject Vaccination History	X						
Maternal Medical History	X						
Maternal Vaccination History	X						
Physical Examination	X		X		X		X
Vital Signs	X		X		X		X
Inclusion & Exclusion Criteria	X						
Blood Sampling (approx. 5 ml)	BL1						BL2
Randomization	X						
Concomitant Medications or Vaccines information	X		X		X		X
Review of Temporary & Definitive Contraindications			X		X		
Vaccination(s)	X		X		X		
30-minute Observation	X		X		X		
Vital Signs 30 minute after Vaccination	X		X		X		
Diary Card (DC) Given	DC1		DC2		DC3		
Diary Card Collected			DC1		DC2		DC3
Collection of solicited injection site & systemic reactions	Day 0–7 after each vaccination						
Collection of unsolicited adverse events	Day 0–28 after each vaccination						
Completion at End of study in Stage 1							X
Collection of serious adverse events	To be reported at any time during the study						

*Staff will contact the subjects' parent / LAR by telephone to check the health status of the subjects, remind them to complete the DC and contact the site in case of any SAE.

Table of Study Procedures – Stage 2

Phase III Study, 2 Visits, 1 Phone call, 1 Vaccination, 2 Blood Samples, 28 Days' Duration Per Subject

**Visit Number (V)**	**V05**	**PC4***	**V06**
**Trial Timelines Days**	**D0**	**D8**	**D28**
**Visit Intervals**		**V05 + 8-10 D**	**V05 + 28-35 D**
Informed Consent	X		
Demography	X		
Subject Medical History & Ongoing Diseases	X		
Subject Vaccination History	X		
Physical Examination	X		X
Vital Signs	X		X
Inclusion & Exclusion Criteria	X		
Blood Sampling (approx. 5 ml)	BL3		BL4
Concomitant Medications or Vaccines information	X		X
Vaccination	X		
30-minute Observation	X		
Vital Signs 30 minute after Vaccination	X		
Diary Card (DC) Given	DC4		
Diary Card Collected			DC4
Collection of solicited injection site & systemic reactions	Day 0–7 after vaccination		
Collection of unsolicited adverse events	Day 0–28 after vaccination		
Completion at End of study			X
Collection of serious adverse events	To be reported at any time during the study		

*Staff will contact the subjects' parent / LAR by telephone to check the health status of the subjects, remind them to complete the DC and contact the site in case of any SAE.

List of Abbreviations

aGMC	adjusted geometric mean concentration
AE	adverse event
AESI	adverse event of special interest
BCG	Bacille Calmette Guerin
°C	degree celsius
CI	confidence interval
CDM	clinical data management
CNS	central nervous system
CRA	Clinical research associate
CRB	(electronic) case report book [all the case report forms for a subject]
CRF	case report form
CTA	clinical trial agreement
CTL	Clinical team leader
D	diphtheria
DC	diary card
DCGI	Drug Controller General of India
DT	diphtheria toxoid
DSMB	Data and Safety Monitoring Board
eCRF	electronic case report form
EPI	Expanded Programme on Immunization
°F	degree Fahrenheit
FAS	full analysis set
FHA	filamentous hemaglutinin
Fim	fimbriae
GCP	Good Clinical Practice
GMC	geometric mean concentration
GMCR	geometric mean concentration ratio
GSO	Global Product Safety Officer
GPV	Global Pharmacovigilance
HBs Ab	hepatitis B surface antibody
HBsAg	hepatitis B surface antigen
HBV	hepatitis B virus
HepB	hepatitis B

HHE	Hypotonic hyporesponsive episode
Hib	*Haemophilus influenzae* type b
HIV	human immunodeficiency virus
IAP	Indian Academy of Pediatrics
ICF	informed consent form
ICH	International Conference on Harmonization
IDMC	Independent data monitoring committee
IPV	inactivated polio vaccine
IRB / IEC	Institutional Review Board / Independent Ethics Committee
IU	international unit
kg	kilogram
LAR	legally acceptable representative
LLOQ	lower limit of quantitation
MedDRA	Medical Dictionary for Regulatory Activity
µg	microgram
mg	milligram
mL	milliliter
MMR	measles mumps rubella
MSD-ECL	Meso Scale Discovery Electrochemiluminescence
NID	national immunization day
NRA	National regulatory authority
OPV	oral polio vaccine
ORV	oral rotavirus vaccine
PPAS	per-protocol analysis set
PRN	pertactin
PRP	polyribosyl ribitol phosphate
PT	pertussis toxin
PV	Pharmacovigilance
RCDC	Reverse Cumulative Distribution Curve
rDNA	recombinant deoxyribonucleic acid
SAE	serious adverse event
SMT	safety management team
T	tetanus
TT	tetanus toxoid
VRR	vaccine response rate

WHO	World Health Organization
wP	whole cell pertussis

1 Introduction

1.1 Background

This is a study of pentavalent vaccine (DTwP-HepB-Hib) Shan 5® indicated for prevention of diphtheria, tetanus, pertussis (whooping cough), hepatitis B (Hep B) and invasive *Haemophilus influenzae* type b (Hib) diseases. Immunization saves millions of lives and is widely recognized as one of the world's most successful and cost-effective health interventions. (1) However, there are still global disparities with regard to vaccine use and coverage, associated mainly with inequalities in the access to health care services. To improve the delivery of vaccines to infant populations, combination vaccines are regarded as an important tool (2) (3) allowing the concomitant administration of several antigens in a single injection. Combination vaccines can also reduce the cost of the delivery of immunization programs and increase compliance with vaccination (4).

Diphtheria is an acute infectious disease affecting the upper respiratory tract and occasionally the skin. All age groups can be affected by the infection caused by *Corynebacterium diphtheriae*, which is usually transmitted through respiratory or hand-to-mouth contact. A toxin produced by the bacteria may cause cardiac and neuronal damage and may lead to death from heart failure or general collapse. Disease prevention lies in vaccination that has led to decreases in the incidence of diphtheria. After EPI implementation began in 1977 with diphtheria vaccine as one of the original six EPI antigens, the incidence of diphtheria worldwide dramatically decreased. Reported diphtheria cases declined from almost 10,000 cases per year during 2000- 2004 to 5288 per year during 2005-2009. However, since 2009 annual reported cases have levelled off. Progress in decreasing diphtheria incidence worldwide has stalled over the past 10 years. The South-East Asia Region, particularly India, is the major driver of global diphtheria incidence trends. (5) In the period 2011–2015, India had the largest total number of reported cases each year, with a 5-year total of 18,350 cases, followed by Indonesia and Madagascar with 3203 and 1633 reported cases respectively (6).

Under the EPI the goal was to achieve 90% or more immunization of 1-year old children by year 2000. By 2016, the global DTP3 coverage was 86%, and the countries of African region reported DTP3 coverage of 74% (7) (8). Reported DTP3 coverage in India in 2016 and 2017 was 88% (9).

Tetanus is an infection caused by *Clostridium tetanii*. The pathogen is widespread in the environment mostly as spores. Animals and humans are susceptible to the disease. Infection usually occurs through scratches or wounds in the skin. The clinical presentation results from the action of its toxin on the Central Nervous System (CNS). Clinical manifestations are muscle spasms and spasm-related complications. There are three major clinical syndromes associated with tetanus: localized, generalized and cephalic. Tetanus is a highly fatal disease. A decline in tetanus incidence in industrialized countries began in the early 1900s. After more than 50 years of use of tetanus toxoid-containing vaccine in industrialized countries, and more than 30 years in developing world, the number of tetanus cases and deaths has decreased dramatically. WHO estimates that in 2015, approximately 34,000 neonates dies from neonatal tetanus. This represents a 96% reduction since 1988 and demonstrates a significant progress towards the global maternal and neonatal tetanus elimination (MNTE) goal. However, many cases occur outside the reach of the health system and are not reported. In 2015, a total of 10,301 tetanus cases including 3551 neonatal cases were

reported through the WHO/ UNICEF Joint Reporting Form, reflecting the low reporting sensitivity for tetanus cases and uncertainty about the true disease incidence (10).

Pertussis, or whooping cough, is caused by *Bordetella pertussis* and transmission is via person to person contact. Parents and siblings are common sources of infection for infants. Pertussis begins with mild upper respiratory tract symptoms (catarrhal stage), progresses to cough and then to paroxysms of cough (paroxysmal stage) characterized by an inspiratory whoop commonly followed by vomiting. Fever is absent or minimal. Symptoms wane gradually over weeks to months (convalescent stage). The duration of classic pertussis is 6 to 10 weeks, in the pediatric population. Widespread vaccination has dramatically reduced the incidence of pertussis in developed countries. However despite the introduction of large-scale vaccination, whooping cough is still an endemic disease with outbreaks occurring every 2 to 5 years (11). It was estimated that without vaccination there would have been >1.3 million pertussis related deaths globally in 2001. In 2013, according to WHO estimates, pertussis was still causing around 63 000 deaths in children aged < 5 years, although there is considerable uncertainty over these estimates in view of the paucity of reliable surveillance data, particularly from developing countries. In 2014 global vaccination coverage with 3 doses of a pertussis-containing vaccine was estimated at 86% (12)..
The main aim of pertussis vaccination is to reduce the risk of severe pertussis in infants and young children due to the high morbidity and mortality caused by the disease in this age group. Protection can be obtained after a primary series of vaccination with either whole cell pertussis (wP) or acellular pertussis (aP) vaccine. Although local and systemic reactogenicity are more commonly associated with wP containing vaccines, both vaccines have excellent safety records (12).
A shift in the age distribution of pertussis towards older age groups (adolescents and young adults) has been reported in recent years in some high income countries, in particular where aP vaccines have replaced wP vaccines for primary vaccination series. Although the reasons for the resurgence of pertussis in a number of countries were found to be complex and varied by country, the shorter duration of protection and probable lower impact of aP vaccines on infection and transmission are likely to play critical roles (12).
Therefore, WHO currently recommends that National programs currently administering wP vaccination should continue to use wP vaccines for primary vaccination series (12).

***Haemophilus influenzae* type b (Hib):** Haemophilus influenzae is a gram-negative coccobacillus which exists in encapsulated and non-encapsulated strains, both of which can cause infection. Non-encapsulated strains commonly cause diseases such as otitis media and sinusitis, through contiguous spread from the nasopharynx, while infections secondary to invasion of the bloodstream are usually caused by encapsulated strains. Encapsulated strains are classified according to the chemical composition of the polysaccharide capsule. Six serotypes, types a, b, c, d, e and f, have been identified. Of these, serotype b is responsible for approximately 95% of all invasive disease due to Haemophilus influenzae. Other serotypes and non-encapsulated strains can cause otitis media in children and disease in the elderly and in immunocompromised populations. The Hib capsule is composed of repeating polymers of ribosyl and ribitol-phosphate (PRP), and this is the primary factor associated with virulence of the organism. The structure of the capsule enables the bacterium to evade phagocytosis and thereby facilitates spread via the bloodstream; hence the PRP capsule is an important determinant of the pathogenesis of invasive disease. A specific antibody to the capsule is associated with protection from disease. Like other bacteria-derived polysaccharides, PRP induces a T-independent antibody response. Children are unable to mount robust responses to pure

polysaccharide antigens before 18 months of age, thus making them highly susceptible to invasive Hib disease (13).

Hib conjugate vaccines have been in use since the early 1990s and vaccination with this vaccine is considered a highly effective public health interventions. In the year 2000, before wide spread introduction of Hib vaccine in resource poor countries, Hib was responsible for at least 8.13 million serious disease in children aged < 60 months and 371,000 deaths. By March 2013, 184 countries (95% of WHO Member states, accounting for 81% of children born in 2012) had included conjugate Hib vaccines in their immunization programs. Use of Hib conjugate vaccines has led to dramatic declines of > 90% in invasive Hib disease in the countries which have included these vaccines in the national immunization programmes (13). Approximately 410,000 (19%) of under < 5 deaths in India are due to pneumonia out of which an estimated 70,000 are caused by the Hib (14).

Routine immunization with Hib conjugate vaccines has resulted in a dramatic decrease in the incidence of invasive *H. influenzae* type b disease in the target populations (e.g. routine immunization of Finnish infants has virtually eliminated Hib disease among children younger than five years) (15). Based upon chemical composition the vaccine against Hib is also known by the name PRP-T (Polyribosyl Ribitol Phosphate [Hib polysaccharide] conjugated to Tetanus toxoid protein).

Hepatitis B (Hep B) virus (HBV) is a member of the hepadnavirus group. Hepatitis B causes irritation and swelling (inflammation) of the liver due to infection with the hepatitis B virus (HBV). It is transmitted inhuman body fluids, such as blood and serum, and even can spread by sexual contact, by perinatal transmission, or by use of improper injection techniques. Hepatitis B presents acutely as a nonspecific hepatitis, with approximately 1% resulting on fulminant hepatitis, or as chronic Hepatitis B infection which may be asymptomatic for many years. Most of the burden of HBV-related disease results from infections acquired in infancy through perinatal or early childhood exposure to HBV because infection acquired at an early age is more likely to become chronic than infection acquired later in life. The risk of chronic infection remains high until after 5 years of age when the rate stabilizes at around 5%. (16) (17).

HBV was among the first viruses to be implicated as a cause of a human cancer, and it is now believed to be second in importance only to tobacco as an environmental carcinogen to which man is exposed. Some two billion people worldwide (or one-third of the global population) are or have been infected with this virus. Approximately 360 million of these are chronically infected and at risk of developing the life-threatening complications of cirrhosis, and hepatocellular carcinoma (HCC). HBV infection is the tenth leading cause of deaths worldwide, accounting for between 520,000 and 1.2 million deaths each year. The prevalence of chronic HBV infection varies considerably in different geographical regions and in different populations and the highest prevalence are in the Asia-Pacific region, sub-Saharan Africa, and the Amazon Basin, where more than 8% of the population are chronic carriers of HBV, and between 70 and 98% show serological evidence of having been exposed to the virus. The Asia-Pacific region is home to approximately 75% of all global HBV carriers (18). Long term consequences of Hep B include hepatic insufficiency, cirrhosis and hepatocellular carcinoma which have both an important burden on public health and impact general costs of public/private health systems (19).

Major progress in the global response to viral hepatitis has been achieved through the expansion of routine hepatitis B vaccination, which was facilitated by the introduction of new combination

vaccines. In 2015, global coverage with 3 doses of hepatitis B vaccine during infancy reached 84%. Between the date of introduction of the vaccine, (ranging from the 1980s to the early 2000s in different countries) and 2015, the proportion of children <5 years of age who became chronically infected fell from 4.7% to 1.3%. (17)

1.2 Background of the Investigational Product

The prevention of diphtheria, tetanus, pertussis, hepatitis B, and invasive infections due to Hib are major goals that could be achieved only if a constant high level of immunization coverage is maintained in the population. The combination vaccines are one of the important tools in achieving this.

Shantha Biotechnics Private Limited (SBPL; Shantha) is developing the 4th generation formulation of the licensed fully liquid whole cell pertussis (wP) based pentavalent vaccine, DTwP-HepB-Hib, SHAN 5®. SHAN 5® is a combination of five components: diphtheria toxoid (DT), tetanus toxoid (TT), whole cell *Bordetella pertussis* antigens (wP), recombinant hepatitis B surface antigen (Hep B) and *Haemophilus influenzae* type b (Hib) [polyribosyl ribitol phosphate conjugated to tetanus toxoid (PRP~T)].

The investigational 4th generation formulation is similar to the currently licensed 3rd generation SHAN 5® except for the following: the source of the Hep B antigen for 3rd generation SHAN 5® is Shantha, India (using the *Pichia pastoris* yeast platform) while the source of the Hep B antigen for the 4th generation SHAN 5® will be the Sanofi Pasteur site at Pilar, Argentina (using the *Hansenula polymorpha* yeast platform). The 4th generation SHAN 5® will henceforth replace the 3rd generation SHAN 5® so as to keep a common source of Hep B antigen for all Sanofi Pasteur / Shantha products where this antigen is used. As per Indian regulatory requirements, a bridging study is required to be conducted for licensure of the 4th generation SHAN 5®.

Since its initial development, several generations of the SHAN 5® product have been developed, and a brief history of the clinical development of SHAN 5® is as follows:

DTPwHBHib-Liq/2005/0100 clinical study : In 2006-2007, **1st generation SHAN 5®** was evaluated in a randomized controlled trial done in India (19) which compared **1st generation SHAN 5®** (n=200) to the DTwP-HB-Hib pentavalent vaccine from Panacea, India (Easyfive™) (n=100) and the DTwP-HB (Tritanrix™ HB) tetravalent vaccine and Hib (Hiberix™) standalone vaccine from GSK (n=100). This trial was done in infants who received a 3-dose infant series at 6-10-14 weeks of age and neither co-administration data nor toddler vaccination data had been generated. All infants were enrolled irrespective of their Hep B vaccination at birth status.

- The 1st **generation SHAN 5®** vaccine was licensed in India in 2007 and the WHO accorded pre-qualification status in June 2008.
- In July 2010, the 1st **generation SHAN 5®** vaccine was delisted from WHO PQ list due to post-marketing vaccine quality complaints regarding the presence of white sediments sticking to the SHAN 5® vaccine vials which could not be re-suspended even after shaking the vial.

SH501 study: In 2012-2013, a phase III, multi-centre, randomized, single blinded study was conducted across India in two cohorts: 15 toddlers were evaluated for safety and immunogenicity following a single booster dose following a real-life priming with Indian licensed vaccines (Cohort

1) followed by 1085 infants (Cohort 2) evaluated for immunogenicity and safety following three-dose primary immunization of the **2nd generation SHAN 5®** (manufactured with the *B. pertussis* antigens imported from Sanofi Pasteur site at Marcy l'Etoile (MLE), France and D, T, Hep B and Hib antigens from Shantha) or a locally licensed comparator DTwP-HB-Hib pentavalent vaccine (**Pentavac SD, Serum Institute of India**). For Cohort 2, all infants were enrolled whatever their Hep B vaccination at birth status, and no toddler vaccinations step was planned it the trial. The objectives of this trial (Cohort 2) were to assess Lot-to-Lot consistency for immunogenicity among three lots of the investigational SHAN 5® vaccine, and immune non-inferiority analysis of the pooled (three lots) data of SHAN 5® vs. the comparator vaccine. The vaccines demonstrated comparable safety and immune responses in cohort 1. In cohort 2, immunogenicity consistency among the three SHAN 5® lots was observed for all antigens except for the *B. pertussis* antigens (for which responses were measured by a commercial EIA [Novalisa from Novatech] designed to assess PT and FHA antibodies), where a marginal failure of the non-inferiority (clinical margin pre-defined as 10 % difference in the vaccine response rate using the assay seropositivity threshold defined as 11 Novatec Units) demonstration was observed and concluded to not have any clinical significance. Immunogenicity non-inferiority against the comparator vaccine was demonstrated for DT, TT, PRP~T and HBs antigens. Safety results were comparable between vaccine groups (20).

- Based on the SH501 study results, the **2nd generation SHAN 5®** vaccine was re-licensed in India in March 2014 by DCG(I) and the WHO re-accorded pre-qualification status in April 2014.

SH505 study: In 2015, a phase III, multi-center, randomized, two arm, single blinded study was conducted on 1040 Indian infants followed up for safety and immunogenicity for 28 days following three doses of the vaccine administered at 6-8, 10-12 and 14-16 weeks of age. All infants were enrolled whatever their Hep B vaccination at birth status. One arm received the investigational **3rd generation SHAN 5®** (with Shantha *B. pertussis* antigens made from MLE seed strains and technology transfer from MLE; rest of the antigens D, T, Hep B and Hib from Shantha) and the other arm received the licensed **2nd generation SHAN 5®** (with MLE *B. pertussis* antigens and D, T, Hep B and Hib from Shantha). Non-inferior immunogenicity analysis of 3rd generation SHAN 5® vs. 2nd generation SHAN 5® was performed for all antibodies to all vaccine antigens. The study results showed a marginal miss of the non-inferiority demonstration for pertussis responses (still measured by the same commercial EIA [Novalisa from Novatech] designed to assess PT and FHA antibodies). However a post-hoc analysis conducted, demonstrated non-inferiority using GMT ratio (21).

- Based on the SH505 study results, the Indian NRA (DCGI) approved this new generation of SHAN 5®. In addition, WHO maintained the pre-qualification status for this **3rd generation SHAN 5®** in 2017 and has asked Shantha to conduct a post WHO PQ commitment study to further confirm the non-inferiority of SHAN 5® with Shantha *B. pertussis* antigens versus SHAN 5® with MLE *B. pertussis* antigens with regards to their pertussis responses.

Please refer to the current version of the Investigator's Brochure for more details of the investigational product.

1.3 Potential Benefits and Risks

1.3.1 Potential Benefits to Subjects

Subjects participating in this clinical trial will be gain benefited from vaccination with the investigational or comparator vaccine formulations against the five major infectious diseases, as described earlier. In all subjects, there will be a close follow-up from the investigators and from delegated study personnel.

Parents/ Legally Acceptable representative (LAR) may access the results from the antibody determinations after vaccination once available with investigator. As with any vaccine, vaccination with the investigational vaccine may not result in desirable protective response(s). In such scenario, vaccination with the registered vaccine will be offered to the non-responders in the investigational vaccine arm.

1.3.2 Potential Risks to Subjects

The potential risks of the investigational vaccine are injection site tenderness, injection site erythema, injection site swelling, fever, vomiting, crying abnormal, drowsiness, loss of appetite and irritability.

As with any other vaccines, on a rare occasion, DTP containing combination vaccines may be associated with anaphylaxis.

Occasionally, DTP containing combination vaccines may be associated with febrile seizures, and may occur at increased rates in children with personal or family histories of convulsions.

However, simple febrile convulsions, although distressing, are considered benign with no evidence that seizure after DTP induce epilepsy.

Another uncommon reaction termed as Hypotonic Hyporesponsive Episode (HHE) has been documented to very rarely occur after immunization with DTP containing combination vaccines. HHE that usually has its onset within 12 hours of injection may last for several hours but always resolves.

Encephalopathy as a syndrome can have many different causes, including infectious diseases, metabolic disturbances, trauma and others. Immune system mediated inflammatory reaction of the brain tissue is suspected with tetanus-toxoid containing vaccines. Other non-vaccine causes include infection and trauma. Molecular mimicry, a situation in which viral/vaccine polypeptides share common antigenic determinants with central or peripheral nerve myelin, could result in a distant autoimmune reaction. Alternatively, infection/immunization may result in perturbation of immunoregulatory mechanisms, interfering with self-tolerance of host myelin proteins. (22) It should be noted, however, that according to Council for International Organizations of Medical Sciences (CIOMS), the evidence is inadequate to either accept or reject a causal relationship between diphtheria toxoid–, tetanus toxoid–, or wP–containing vaccine and encephalopathy. (23)

Neither death nor adverse sequelae have been observed after these episodes.

Anaphylaxis, convulsions, encephalopathy and HHE are considered as AESI and will be closely monitored by Sponsor's PV department. All AESIs will be considered as serious adverse events (SAEs).

In addition, pain and bruising could occur following the blood sampling.

As in any clinical trial, the subject's health status will be checked carefully so that any unexpected reactions can be identified.

1.4 Rationale for the Study

This trial will evaluate immunogenicity of investigational formulation (4th generation) of SHAN 5® versus the comparator licensed formulation (2nd generation) of SHAN 5® with respect to their *B. pertussis* and Hep B components. This study will be conducted in order to obtain licensure in India for the 4th generation SHAN 5® to qualify the switch of Hep B Drug Substance source from Shantha to Pilar in 4th generation SHAN 5® and will also address the post PQ commitment to WHO to confirm the non-inferiority of Shantha pertussis component versus imported pertussis component of SHAN 5®. The study will also provide additional data on persistence of antibodies until toddler booster vaccination and safety and immunogenicity data of SHAN 5® when used as a booster in toddlers primed with SHAN 5®.

For the purpose of the study, 4th generation SHAN 5® (with Shantha pertussis and imported HBsAg) will henceforth be referred to as the investigational formulation; and the 2nd generation (licensed formulation with imported pertussis and Shantha HBsAg) will henceforth be referred to as the comparator or licensed formulation.

2 Study Objectives

2.1 Primary Objective(s)

1) To demonstrate the non-inferiority of the investigational SHAN 5® formulation *versus* the licensed SHAN 5® formulation in terms of Hep B seroprotection rate, 28 days after a three-dose primary series

2) To demonstrate the non-inferiority of the investigational SHAN 5® formulation *versus* the licensed SHAN 5® formulation in terms of pertussis immune responses, 28 days after a three-dose primary series.

The endpoint(s) for the primary objective(s) are presented in Section 9.1

2.2 Secondary Objective(s)

Safety:

1) Stage 1: To describe the safety profile of investigational SHAN 5® formulation and licensed SHAN 5® formulation up to 28 days after each dose of the primary series when administered concomitantly with other age-recommended vaccines.

2) Stage 2: To describe the safety profile of investigational SHAN 5® formulation up to 28 days after the booster dose when administered concomitantly with other age-recommended vaccines .

Immunogenicity:

3) Stage 1: To describe the immunogenicity profile, at baseline and 28 days after a three-dose primary series of investigational SHAN 5® formulation and licensed SHAN 5® formulation in terms of seroprotection rates, seroresponse rates and GMCs to all antigens (D, T, Hep B, Pertussis and Hib) when administered concomitantly with other age-recommended vaccines.

4) Stage 2: To describe the persistence of antibodies against investigational SHAN 5® formulation and licensed SHAN 5® formulation at 12-24 months of age following a 3-dose primary series at 6-8, 10-12, and 14-16 weeks of age, in terms of seroprotection / seroresponse rates and GMCs to all antigens D, T, Hep B, Pertussis and Hib.

5) Stage 2: To describe the immunogenicity profile, 28 days after the single booster dose of investigational SHAN 5® formulation in subjects 12-24 months of age who have been primed with either investigational SHAN 5® formulation or licensed SHAN 5® formulation, in terms of seroprotection rates, seroresponse rates and GMCs to all antigens (D, T, Hep B, Pertussis and Hib) when administered concomitantly with other age-recommended vaccines

The endpoint(s) for the secondary objective(s) are presented in Section 9.2

3 Investigators and Study Organization

This study will be conducted in approximately 8 centers in India. This is a multi-center study with multiple investigators. Details of the study centers, and the Investigators at each center are provided in the "List of Investigators and Centers Involved in the Trial" document.

An internal safety management team (SMT) will perform an analysis of safety data during the conduct of the study.

The Sponsor's Responsible medical officer (the person authorized to sign this protocol and any amendments on behalf of Sponsor) is [redacted] Clinical Team Leader.

Apart from this the Investigators involved in this trial across various sites will also sign in signature page of the protocol, mentioning that they have gone through as well as understand this protocol and ready to conduct this trial according to the protocol, Good Clinical Practice and applicable local regulatory requirements.

The Sponsor will provide clinical trial supplies (vaccines and other study material).The monitoring of the clinical trial will be done by the Shantha's Clinical Operations department.

Table 1: Trial Organization

Sponsor's Responsible Medical Officer	Name: [redacted]<br>Designation: AGM, Clinical R&D<br>Address: Shantha Biotechnics Limited, 4th Floor, Vasantha Chambers, Fateh

	Maidan Road, Basheer Bagh, Hyderabad. 500004.<br>Email:[redacted]
Sponsor's Product Safety Officer (PSO)	Name: [redacted]<br>Designation: AGM, Pharmacovigilance<br>Address: Shantha Biotechnics Limited, 4th Floor, Vasantha Chambers, Fateh Maidan Road, Basheer Bagh, Hyderabad. 500004.<br>Phone: [redacted]<br>Mobile:[redacted]<br>Email: [redacted]
Clinical Data Management	Name:[redacted]<br>Designation: Director – Clinical Data Management<br>Address: Zifo Technologies, 21A Annasalai, Littlemount, Saidapet, Chennai, Tamil Nadu, India-600015.<br>Email: [redacted]
Biostatistics	Name:[redacted]<br>Designation: Biostatistics Team Lead<br>Address: Sanofi Pasteur, Marcy-l'Etoile, France<br>Email: [redacted]
Analytical Laboratory	Global Clinical Immunology (GCI)<br>Name: [redacted]<br>Designation: Head, Department of Global Clinical Immunology<br>Address: Sanofi Pasteur Inc. 1 Discovery Drive, Swift water, PA 18370, USA<br>Email:[redacted]

4 Independent Ethics Committee / Institutional Review Board

Before the investigational product can be shipped to the investigational site and before the inclusion of the first subject, this protocol, the informed consent form (ICF), subject recruitment procedures, and any other written information to be provided to subjects must be approved by, and / or receive favorable opinion from, the appropriate Independent Ethics Committee (IEC) or Institutional Review Board (IRB).

In accordance with Good Clinical Practice (GCP) and local regulations, each Investigator and / or the Sponsor are responsible for obtaining this approval and / or favorable opinion before the start of the study. If the protocol is subsequently amended, approval must be re-obtained for each

substantial amendment. Copies of these approvals, along with information on the type, version number, and date of document, and the date of approval, must be forwarded by the Investigator to the Sponsor together with the composition of the IEC / IRB (the names and qualifications of the members attending and voting at the meetings).

The Investigator with Sponsor's support will submit written summaries of the status of the study to the IEC / IRB annually, or more frequently if requested. All serious adverse events (SAEs) occurring during the study will be reported by the Investigator to the IEC / IRB, according to the IEC / IRB policy.

5 Investigational Plan

5.1 Description of the Overall Study Design and Plan

5.1.1 Study Design

This is a phase III, multi-center, randomized, active controlled, two arm, observer blind (subject's parents/LAR, Investigator and study staff except the person in charge of the vaccination are blinded to the study vaccination) study in 460 infants followed up for safety and immunogenicity for 28 days after administration of three doses of either investigational or licensed vaccine formulation at 6-8, 10-12 and 14-16 weeks of age. Followed by single arm, open label study in the subjects upon attaining 12-24 months of age followed up for safety and immunogenicity for 28 days after administration of single booster dose of the investigational vaccine formulation.

The study will have 2 stages:

Stage 1: Infants aged 6-8 weeks of age will be randomly allocated at 1:1 ratio, to receive 3 doses of either the investigational or the licensed SHAN 5® vaccine formulation at 6-8, 10-12 and 14-16 weeks of age, irrespective of whether they received Hepatitis B vaccination at birth or not. Available licensed oral rotavirus vaccine (ORV) and oral poliovirus vaccine / inactivated poliovirus vaccine (OPV / IPV) will be co-administered at 6-8, 10-12 and 14-16 weeks of age as per standard of care. Neither safety nor immunogenicity evaluations of these co-administered vaccines will be performed.

Stage 2: Toddlers aged 12-24 months who had received either the investigational or the licensed SHAN 5® vaccine formulation at 6-8, 10-12 and 14-16 weeks of age in stage 1, will receive a single booster dose of the investigational SHAN 5® vaccine formulation. Subjects will concomitantly receive a booster dose of OPV / IPV and a 2nd dose* of Measles Mumps Rubella (MMR) vaccine at the recommended age as per the standard of care.

* Post completion of Stage 1 and prior to the study initiation of Stage 2, subjects will be provided 1st dose of MMR at the recommended age.

5.1.2 Justification of the Study Design

The design of this study is intended to meet the requirement of the Drug Controller General of India (DCGI) for use of SHAN 5® with imported HBsAg component from Pilar,, Argentina in place of SHAN 5® with Shantha HBsAg component. The study is also designed to address the post PQ commitment to WHO to confirm the non-inferiority of Shantha pertussis component versus imported pertussis component of SHAN 5®. The study will be conducted in infants aged 6-8 weeks (stage 1) which is the targeted population to receive the primary series of D, T, P, Hep B and Hib vaccination as per the EPI schedule in India. The stage 2 of the study in toddlers will provide additional data on persistence of antibodies until the age of 12-24 months. The stage 2 of the study in toddlers will also provide safety and immunogenicity data of SHAN 5® when used as a toddler booster in subjects primed with SHAN 5®.

The study in infants (Stage 1) will be observer blind, i.e. parent/LAR of the subjects, Investigator and study staff except the person in charge of the vaccination will not be aware of the group allocation. Lab personnel performing immunogenicity assessments also will be blinded to the study vaccine or group allocation. This will help in reducing the observer bias for study assessment. Randomization will help in reducing the selection bias.

The study in toddlers (Stage 2) will be open label single arm study. All the subjects in toddler stage will be administered the investigational SHAN 5® as the only available vaccine batch of the comparator licensed SHAN 5® vaccine formulation (2nd generation) will have been expired (batch expiry date - June 2019) by the time of the toddler stage of the study. The dosing of the infant series in the study is planned to be completed well before this expiry date.

Concomitant vaccines will be administered in the study as recommended in the Indian Academy of Pediatrics (IAP) immunization schedule. Safety of SHAN 5® vaccine when administered along with the recommended vaccines for the age will be described. Immunogenicity evaluations of these co-administered vaccines will not be performed. No clinically relevant interference has been observed previously when OPV / IPV is used in association with diphtheria-tetanus-whole cell pertussis (DTwP) / diphtheria–tetanus–acellular pertussis (DTaP), Hib, hepatitis B, pneumococcal polysaccharide conjugate or rotavirus vaccines. (24)

In order to collect information across India with various geographical locations, the study will be conducted at approaching multiple sites across India (multi-center study).

5.1.3 Study Plan

Eligible subjects will be identified, consented, screened and recruited. Before screening the subject, subject's parent/legally acceptable representative (LAR) will be explained about the study and its procedure in detail which will be recorded through Audio-Visual process following signing of the Audio-Visual consent form by them. After signing of ICF, subject will be screened as per the study criteria whether he/she fulfills all criteria to participate in the study. Then he/ she may be recruited in the study.

The study will have 2 stages:

Stage 1: Infants aged 6-8 weeks of age will be randomly allocated at 1:1 ratio, to receive 3 doses of either the investigational or the licensed SHAN 5® vaccine formulation at 6-8, 10-12 and 14-16 weeks of age. No stratification will be made at enrolment on the basis of Hep B vaccination at birth. Available licensed oral rotavirus vaccine (ORV) and oral poliovirus vaccine / inactivated poliovirus vaccine (OPV / IPV) will be co-administered at 6-8, 10-12 and 14-16 weeks of age.

Stage 2: Toddlers aged 12-24 months who had received either the investigational or the licensed SHAN 5® vaccine formulation at 6-8, 10-12 and 14-16 weeks of age in stage 1, will receive a single booster dose of the investigational SHAN 5® vaccine formulation.

Subjects in stage 2 will concomitantly receive a booster dose of OPV / IPV and a 2nd dose of Measles Mumps Rubella (MMR) vaccine at the recommended age.

> *Post completion of Stage 1 and prior to the study initiation of Stage 2, subjects will be provided 1st dose of MMR at the recommended age.

Visits/phone calls:

During the trial period there will be:
Stage 1: 4 planned visits, 3 Phone calls

Stage 2: 2 planned visits, 1 Phone call

Blood sampling:

Stage 1: All subjects will provide a pre-vaccination (baseline) blood sample at Day 0 and a post-vaccination sample at Day 84 (+7 days). Approximately 5 mL of blood will be collected.

Stage 2: All subjects will provide a pre-booster dose vaccination (baseline) blood sample at Day 0 and a post-vaccination sample at Day 28 (+7 days). Approximately 5 mL of blood will be collected.

Collection of safety data:

Stage 1: All subjects will be observed for 30 minutes after each vaccine dose, and clinical site personnel will record any unsolicited systemic AEs occurring during that time as immediate unsolicited systemic AEs.

The parents/LAR of subjects will record in the Diary cards information about solicited injection site and systemic reactions for 7 days after each vaccine dose and any unsolicited AEs that may occur for 28 days after each vaccine dose.

Information on SAEs (including AESIs) will be collected throughout the trial period (from Day 0 to Day 84 (+7 days)).

Stage 2: All subjects will be observed for 30 minutes after the vaccine dose, and clinical site personnel will record any unsolicited systemic AEs occurring during that time as immediate unsolicited systemic AEs.

The parents/LAR of subjects will record in the Diary cards information about solicited injection site and systemic reactions from D0 to D7 post-vaccination and unsolicited AEs from D0 to D28 post-vaccination.

Information on SAEs (including AESIs) will be collected throughout the trial period (from Day 0 to Day 28 (+7 days)).

Duration of Participation in the Study:

Stage 1: The duration of each subject's active participation in the study will be approximately 84 days

Stage 2: The duration of each subject's active participation in the study will be approximately 28 days

5.1.4 Visit Procedures

5.1.4.1 Stage 1 (Infants)

Visit 1 (Day 0): Inclusion, Randomization, and Vaccination

1) Take signature from subject's parent / legally acceptable representative (LAR) on Audio-Visual (A-V) consent form
2) Give the subject's parent / legally acceptable representative (LAR) information regarding the study (objectives, procedures etc.), answer any questions and make sure the subject's parent(s)/ LAR understand(s) the study. Obtain written informed consent. This entire process will be conducted under A-V recording. Give the subject's parent / LAR a signed copy of the informed consent form (ICF). Keep the original signed ICF in the Investigator site file (ISF).
3) Allocate screening number to the subject.
4) Collect demographic data of the subject.
5) Obtain verbal medical history about the subject.
6) Collect subject's Vaccination history details since birth.
7) Collect maternal medical history.
8) Collect maternal Vaccination history during pregnancy.
9) Conduct a physical examination of the subject.
10) Take the subject's vital signs: axillary temperature, respiratory rate and pulse rate.
11) Check inclusion and exclusion criteria for eligibility. Enroll or include the eligible subject in the study and exclude the ineligible subject from the study. Irrespective of subject's eligibility, capture the subject's details in a log entitled "Screening and enrollment log".
12) Fill the (eligible) subject's contact and identification details in "Subject Identification Log".
13) Obtain the first blood sample - Collect approx. 5 ml of blood sample (BL1) and fill details of the same in blood sample collection log (see Section 7.1 for detailed instructions regarding the handling of blood samples).

14) Allocate inclusion/enrollment vaccine group to subject which will correspond to subject / randomization number.(Refer to Section 6.5)
15) Collect subject's concomitant medications or vaccines information
16) Administer the first dose (Dose 1) of the study vaccine as per randomization and fill details of the same in the study product administration log (see Section 7.1 for detailed instructions regarding administration of study vaccines). Also, enter the subject specific details in the space provided on the inner box (carton) of the study vaccines.
17) Administer the concomitant vaccines – ORV and OPV / IPV
18) Keep the subject under observation for 30 minutes, and record any adverse reaction in the source document.
19) Measure vital signs information such as axillary temperature, respiratory rate and pulse rate, 30 minutes after the vaccination.
20) Give the parent / LAR a diary card (DC1), a thermometer, and a ruler, and go over the instructions for their use.
21) Remind the parent / LAR to expect a telephone call 8 days after Visit 1 and to bring back the Diary card when they return for Visit 2 on at the specified date.
22) Remind the parent / LAR to notify the site in case of an SAE.
23) Complete the relevant case report forms (CRFs) for this visit.

Telephone Call 1 (PC1; 8 (+2) days after Visit 1)

Note: If Day 8 falls on a weekend or a holiday, the telephone call may be made on the following business day.

1) Record relevant information concerning the subject's health status on the telephone contact form. If an SAE occurred, follow the instructions in Section 9 for reporting it.

2) Remind the parent / LAR to do the following:
   - Complete the Day 0–7 pages of the diary card.
   - Complete the remaining pages of the diary card, and bring them to next visit.
   - Notify the site in case of an SAE.

Visit 2 (28 [+7] days after Visit 1): Collection of Safety Information and Vaccination

1) Collect diary card (DC1) and review the information filled by subject's parent/LAR, including any adverse events (unsolicited non-serious AEs and SAEs) that might have occurred since Visit 1.
2) Collect concomitant medication details that have been given to subject since Visit 1.
3) Conduct a general physical examination of the subject.

4) Measure vital signs information such as axillary temperature, respiratory rate and pulse rate, before vaccination.
5) Check for Temporary/Definitive Contraindications
6) Administer the second dose (Dose 2) of appropriate study vaccine intramuscularly as indicated on the randomization sheet and fill details of the same in study product administration log. Also, enter the subject specific details in the space provided on the inner box (carton) of the study vaccines.
7) Administer the concomitant vaccines – ORV and OPV / IPV
8) Keep the subject under observation for 30 minutes and record any immediate unsolicited systemic adverse events.
9) Measure vital signs information such as axillary temperature, respiratory rate and pulse rate, 30 minutes after the vaccination.
10) Give second diary card (DC2) to the parent/LAR and go over the instructions for their use
11) Remind the parent / guardian / legally acceptable representative to expect a telephone call 8 days after Visit 2 and to bring back the Diary card when they return for Visit 3 on the specified date.
12) Remind the parent / LAR to notify the site in case of an SAE.
13) Complete the relevant case report forms (CRFs) for this visit.

Telephone Call 2 (PC2; 8 (+2) days after Visit 2)

Follow the same steps as mentioned for Telephone call 1 (PC1)

Visit 3 (28 [+7] days after Visit 2): Collection of Safety Information and Vaccination

1) Collect diary card (DC2) and review the information filled by subject's parent/LAR, including any adverse events (unsolicited non-serious AEs and SAEs) that might have occurred since Visit 2.
2) Collect concomitant medication details that have been given to subject since Visit 2.
3) Conduct a general physical examination of the subject.
4) Measure vital signs information such as axillary temperature, respiratory rate and pulse rate, before vaccination.
5) Check for Temporary/Definitive Contraindications
6) Administer the third dose (Dose 3) of appropriate study vaccine intramuscularly as indicated on the randomization sheet and fill details of the same in study product administration log. Also, enter the subject specific details in the space provided on the inner box (carton) of the study vaccines.
7) Administer the concomitant vaccines – ORV and OPV / IPV

8) Keep the subject under observation for 30 minutes and record any immediate unsolicited systemic adverse events.
9) Measure vital signs information such as axillary temperature, respiratory rate and pulse rate, 30 minutes after the vaccination.
10) Give second diary card (DC3) to the parent/LAR and go over the instructions for their use
11) Remind the parent / guardian / legally acceptable representative to expect a telephone call 8 days after Visit 3 and to bring back the Diary card when they return for Visit 4 on the specified date.
12) Remind the parent / LAR to notify the site in case of an SAE.
13) Complete the relevant case report forms (CRFs) for this visit.

Telephone Call 3 (PC3; 8 (+2) days after Visit 3)

Follow the same steps as mentioned for Telephone call 1 (PC1)

Visit 4 (28 [+7] days after Visit 3): Collection of Safety Information

1) Collect diary card (DC3) and review the information filled by subject's parent/LAR, including any adverse events (unsolicited non-serious AEs and SAEs) that might have occurred since Visit 3.
2) Collect concomitant medication details that have been given to subject since Visit 3.
3) Conduct a general physical examination of the subject.
4) Measure vital signs information such as axillary temperature, respiratory rate and pulse rate, before vaccination.
5) Obtain the second blood sample - Collect approx. 5 ml of blood sample (BL2) and fill details of the same in blood sample collection log (see Section 7.1 for detailed instructions regarding the handling of blood samples).
6) Complete the relevant case report forms (CRFs) for this visit.

5.1.4.2 Stage 2 (Toddlers)

Visit 5 (Day 0): Inclusion and Vaccination

1) Re-consent of the subject's parent / LAR will be taken to ensure the subject parent/LAR are still interested to take part in stage 2. Take signature from subject's parent / LAR on Audio-Visual (A-V) consent form.
2) Give the subject's parent / LAR information regarding the study (objectives, procedures etc.), answer any questions and make sure the subject's parent(s)/ LAR understand(s) the study. Obtain written informed consent. This entire process will be conducted under A-V recording. Give the subject's parent / LAR a signed copy of the informed consent form (ICF). Keep the original signed ICF in the ISF.

3) Obtain verbal medical history about the subject (from V04).
4) Collect subject's Vaccination history details since V04.
5) Conduct a physical examination of the subject.
6) Take the subject's vital signs: axillary temperature, respiratory rate and pulse rate.
7) Check inclusion and exclusion criteria for eligibility. Enroll or include the eligible subject in the study and exclude the ineligible subject from the study. Irrespective of subject's eligibility, capture the subject's details in a log entitled "Screening and enrollment log".
8) Obtain blood sample - Collect approx. 5 ml of blood sample (BL3) and fill details of the same in blood sample collection log (see Section 7.1 for detailed instructions regarding the handling of blood samples).
9) Allocate the same subject ID as in Stage 1. (Refer to Section 6.5).
10) Collect subject's concomitant medications or vaccines information
11) Administer the booster dose of the investigational vaccine and fill details of the same in the study product administration log (see Section 7.1 for detailed instructions regarding administration of study vaccines). Also, enter the subject specific details in the space provided on the inner box (carton) of the study vaccines.
12) Administer the concomitant vaccines – MMR (2nd dose) and/or OPV / IPV (booster dose) as appropriate as per the age of the subject at the time of the visit.
13) Keep the subject under observation for 30 minutes, and record any adverse reaction in the source document.
14) Measure vital signs information such as axillary temperature, respiratory rate and pulse rate, 30 minutes after the vaccination.
15) Give the parent / LAR a diary card (DC4), a thermometer, and a ruler, and go over the instructions for their use.
16) Remind the parent / LAR to expect a telephone call 8 days after Visit 5 and to bring back the Diary card when they return for Visit 6 on at the specified date.
17) Remind the parent / LAR to notify the site in case of an SAE.
18) Complete the relevant case report forms (CRFs) for this visit.

Telephone Call 4 (PC4; 8 (+2) days after Visit 5)

Note: If Day 8 falls on a weekend or a holiday, the telephone call may be made on the following business day.

1) Record relevant information concerning the subject's health status on the telephone contact form. If an SAE occurred, follow the instructions in Section 9 for reporting it.
2) Remind the parent / LAR to do the following:
   - Complete the Day 0–7 pages of the diary card.
   - Complete the remaining pages of the diary card, and bring them to the next visit.
   - Notify the site in case of an SAE.

Visit 6 (28 [+7] days after Visit 5): Collection of Safety Information

1) Collect diary card (DC4) and review the information filled by subject’s parent/LAR, including any adverse events (unsolicited non serious AEs and SAEs) that might have occurred since Visit 5.
2) Collect concomitant medication details that have been given to subject since Visit 5.
3) Conduct a general physical examination of the subject.
4) Measure vital signs information such as axillary temperature, respiratory rate and pulse rate, before vaccination.
5) Obtain blood sample - Collect approx. 5 ml of blood sample (BL4) and fill details of the same in blood sample collection log (see Section 7.1 for detailed instructions regarding the handling of blood samples).
6) Complete the relevant case report forms (CRFs) for this visit.

Unscheduled Visit (Stage 1 or Stage 2):

If visit happens in between the scheduled visits it will be considered as unscheduled visit and the following procedures will be completed for the visit

1) Identify the reason for unscheduled visit (AE/SAE, Early termination visit and other.)

2) Perform a general physical examination.

3) Measure vital signs information such as axillary temperature, respiratory rate and pulse rate.

4) Complete the relevant case report forms (CRFs) for this visit.

.

Follow-up of subjects with Related AEs or with AEs That Led to Study/Vaccination Discontinuation:

A subject who experiences an AE (whether serious or non-serious) during the study must be followed until the condition resolves, becomes stable, or becomes chronic (even after the end of the subject’s participation in the study) if *either* of the following is true:

The AE is considered by the Investigator to be related to the product administered.

The AE caused the discontinuation of the subject from the study or from vaccination.

5.1.5 Planned Study Calendar

The following dates are approximate. The actual dates may differ as, for example, the study will not start until all the appropriate regulatory and ethical approvals have been obtained.

Planned study period - FVFS (first visit, first subject) to LCLS (last contact, last subject) Stage 1 & 2): November 2018 to May 2020

Planned inclusion period - FVFS to FVLS (first visit, last subject) Stage 1: November 2018 to February 2019

Planned primary vaccination period Stage 1: November 2018 to April 2019

Planned date of interim clinical study report (Stage 1 data): November 2019

Planned inclusion period - FVFS to FVLS (first visit, last subject) Stage 2: December 2019 to April 2020

Planned booster vaccination period (Stage 2): December 2019 to April 2020

Planned end of study (LCLS): May 2020

Planned date of final clinical study report: October 2020

5.1.6 Periodic Safety Data Review

The Periodic Safety Data Review is based on preliminary data that has not been subjected to validation and database lock. This Periodic Safety Data Review would be carried out by the Sponsor's Internal Safety Monitoring Team (SMT) constituted for this trial.

The following events will serve as Alert Thresholds for this protocol and will trigger an adhoc Periodic Safety Data Review by the SMT. Enrollment may be paused by the SMT during this review and recommenced after the safety of the vaccine has been established.

- Fatal Events and Related SAEs
- Adverse Events of Special Interest (e.g. Anaphylaxis, Hypotensive Hypo responsive Episodes [HHE] to whole cell pertussis, Encephalopathy and Convulsions (including febrile seizures)

5.2 Enrollment and Retention of Study Population

5.2.1 Recruitment Procedures

Stage 1: Parent(s)/ LAR of an appropriate pool of potential subjects (independent of the Investigator and investigational team) will be approached during their routine visit to the investigational site. The Investigator/ investigational team at study site will take the responsibility of subject recruitment. The site will ensure that any advertisements used to recruit subjects (letters, pamphlets, posters, etc.) are submitted to Shantha prior to submission to the IEC / IRB for approval.

Stage 2: Parent(s)/LAR of the subjects who participated in Stage 1 of the study will be approached as and when their child attains 2nd year of life. The Investigator/ investigational team at trial site will take the responsibility of subject inclusion.

5.2.2 Informed Consent Procedures

If potential subject's parent/ LAR are willing for the subject to participate in the study, signature from them on Audio-Visual (A-V) consent form will be taken before explaining the study and its procedures. This explanation of the study and its procedures will be recorded through Audio-Visual process.

Informed consent is the process by which the subject's parents / LAR voluntarily confirms his or her willingness to participate in a particular study. Informed consent must be obtained before any study procedures are performed. The process is documented by means of a written, signed, and dated ICF.

In accordance with GCP, prior to signing and dating the consent form, the subject's parents / LAR must be informed by appropriate study personnel about all aspects of the study that are relevant to making the decision to participate, and must have sufficient time and opportunity to ask any questions.

If the subject's parents / LAR is not able to read and sign the ICF, then it must be signed and dated by an impartial witness who is independent of the Investigator. A witness who signs and dates the consent form is certifying that the information in this form and any other written information had been accurately explained to and understood by the subject's parents / LAR.

The actual ICF used at each center may differ, depending on local regulations and IEC / IRB requirements. However, all versions must contain the standard information found in the sample ICF provided by the Sponsor. Any change to the content of the ICF must be approved by the Sponsor and the IEC / IRB prior to the form being used.

If new information becomes available that may be relevant to the subject's parent's / LAR's willingness to continue participation in the study, this will be communicated to him / her in a timely manner. Such information will be provided via a revised ICF or an addendum to the original ICF.

Informed consent forms will be provided in duplicate, or a photocopy of the signed consent will be made. The original will be kept by the Investigator, and the copy will be kept by the subject's parents / LAR.

Documentation of the consent process should be recorded in the source documents.

5.2.3 Screening Criteria

There are no screening criteria other than the inclusion and exclusion criteria.

5.2.4 Inclusion Criteria

An individual must fulfill *all* of the following criteria to be eligible for study enrollment:

<u>For Stage 1:</u>

1. Infants between 6-8 weeks of age (42 to 56 days, both days inclusive) on the day of enrollment.

2. Healthy infants, born at full term of pregnancy (≥ 37 weeks) with a birth weight ≥ 2.5 kg or medically stable* prematurely born infants (born after a gestation period of 27-36 weeks).

* Medically stable refers to premature infants who do not require significant medical support or ongoing management for debilitating disease and who have demonstrated sustained growth curve by the time they receive the first dose of study vaccine.

3. Informed consent form signed by one or both parents or by the LAR as per local requirements.

4. Subjects and Parents/LAR are able to attend all scheduled visits and to comply with all study procedures.

<u>For Stage 2:</u>

1. Toddlers aged between 12-24 months of age on the day of enrollment and had received either the investigational or the licensed SHAN 5® vaccine formulation at 6-8, 10-12 and 14-16 weeks of age during stage 1 of the trial.

2. Informed consent form signed by one or both parents or by the LAR as per local requirements.

3. Subjects and Parents/LAR are able to attend all scheduled visits and to comply with all study procedures.

5.2.5 Exclusion Criteria

An individual fulfilling *any* of the following criteria is to be excluded from study enrollment:

1. Participation in another clinical trial in the 4 weeks preceding the trial inclusion or planned participation during the present trial period in another clinical trial investigating a vaccine, drug, medical device, or medical procedure.
2. Receipt of any vaccine in the 4 weeks preceding the first trial vaccination (except BCG, birth dose OPV and birth dose of Hep B vaccine).
3. Planned receipt of any other vaccine within the period from 8 days before to 8 days after each trial vaccination except OPV if not given at birth and during National Immunization Day (NID).
4. For Stage 1 only: Previous vaccination against the diphtheria, tetanus, pertussis, hepatitis B (except the birth dose of Hep B vaccine) or Haemophilus influenza type b infection with the trial vaccine or another vaccine.

5. For Stage 2 only: Receipt of booster vaccination against diphtheria, tetanus, pertussis, hepatitis B, or *Haemophilus influenza* type b infection with the trial vaccine or another vaccine.
6. Past or current receipt of immunoglobulins, blood or blood-derived products or planned administration during the trial.
7. Known or suspected congenital or acquired immunodeficiency; or receipt of immunosuppressive therapy, such as anti-cancer chemotherapy or radiation therapy since birth; or long-term systemic corticosteroid therapy (prednisone or equivalent for more than 2 consecutive weeks since birth).
8. History of diphtheria, tetanus, pertussis, hepatitis B, or Haemophilus influenza type b infections (confirmed either clinically, serologically or microbiologically).
9. Known personal or maternal history of HIV or hepatitis B seropositivity.
10. Known systemic hypersensitivity to any of the vaccine components, or history of a life threatening reaction to the trial vaccine or a vaccine containing the same substances [a]
11. Known thrombocytopenia, as reported by the parent/ legally acceptable representative.
12. Bleeding disorder or receipt of anticoagulants in the 3 weeks preceding inclusion, contradicting intramuscular vaccination.
13. Chronic illness that, in the opinion of the investigator, is at a stage where it might interfere with trial conduct or completion. (Chronic illness may include, but is not limited to, cardiac, renal, autoimmune, hepatic, hematological, genetic disorders, atopic conditions, congenital defects, diabetes, convulsions or encephalopathy etc.).
14. Moderate or severe acute illness/infection (according to investigator judgment) on the day of vaccination or febrile illness (axillary temperature $\geq$ 100.4 °F or $\geq$38 °C) on the day of inclusion (a prospective subject should not be included in the study until the condition has resolved or the febrile event has subsided).
15. Identified as a natural or adopted child of the Investigator, relatives or employee with direct involvement in the proposed study.
16. Subject with definite seizure disorder and getting anticonvulsant therapy.

5.2.6 Medical History

Prior to enrollment, subjects will be assessed for pre-existing conditions and illnesses, both past and ongoing. Any such conditions will be documented in the source document. Significant (clinically relevant) medical history (reported as diagnosis) including conditions/illnesses for which the subject is or has been followed by a physician or conditions/illnesses that could resume during the course of the study or lead to an SAE or to a repetitive outpatient care will be collected in the case report book (CRB). The significant medical history section of the CRB contains a core list of body systems and disorders that could be used to prompt comprehensive reporting, as well as space for the reporting of specific conditions and illnesses.

For each condition, the data collected will be limited to:

Diagnosis (this is preferable to reporting signs and symptoms)

Presence or absence of the condition at enrollment

---

[a] The components of Vaccine X are listed in Section 6.1 and in the Investigator's Brochure.

The reporting of signs and symptoms in lieu of a diagnosis is strongly discouraged.

Dates, medications, and body systems are not to be recorded, and the information collected will not be coded. Its purpose is to assist in the later interpretation of safety data collected during the study.

5.2.7 Contraindications for Subsequent Vaccinations (Stage 1 only)

5.2.7.1 Temporary Contraindications

Should a subject experience one of the conditions listed below, the Investigator will postpone further vaccination until the condition is resolved. Postponement must still be within the timeframe for vaccination indicated in the Table of Study Procedures.

Febrile illness (temperature ≥ 38.0°C [≥ 100.4°F]) or moderate or severe acute illness / infection on the day of vaccination, according to Investigator judgment

Receipt of any vaccine (except oral polio vaccine (OPV)) in the 8 days preceding 2nd and 3rd dose of study vaccination or planned receipt of any vaccine (except OPV) in the 8 days following any dose of the study vaccination.

5.2.7.2 Definitive Contraindications

Should a subject experience one of the conditions listed below, the Investigator will discontinue vaccination:

1. An anaphylactic or other significant allergic reaction to the previous dose of vaccine
2. Participation in another clinical trial investigating a vaccine, drug, medical device, or medical procedure since the previous dose of vaccination.
3. Known thrombocytopenia or a bleeding disorder or receipt of anticoagulants in the 3 weeks preceding vaccination contraindicating intramuscular (IM) vaccination.
4. Known or suspected congenital or acquired immunodeficiency; or receipt of immunosuppressive therapy, such as anti-cancer chemotherapy or radiation therapy or long-term systemic corticosteroid therapy (prednisone or equivalent for more than 2 consecutive weeks) since the previous dose of vaccination.
5. Receipt of immunoglobulins, blood or blood-derived products since the previous dose of vaccination
6. Acute disease or severe chronic illness (cardiac or renal disorders, congenital defects, convulsions, encephalopathy etc. considered to be related to the vaccination by the investigator) that could interfere with the conduct or completion of the trial.
7. Clinical or serological or microbiological confirmed infection due to diphtheria, tetanus, pertussis, Hep B or Hib diseases since the previous dose of vaccination.
8. Administration of any vaccine against the diphtheria, tetanus, pertussis, Hep B or Hib infection since the previous dose of vaccination.
9. Known precautions to further vaccination with a pertussis vaccine i.e.:
    - Encephalopathy within 7days following vaccine administration.

- Temperature >104.9°F or >40.5°C (axillary route) within 48 hours following vaccine injection, not due to another identifiable cause.
- Inconsolable crying for >3 hours within 48 hours following vaccine administration or that is rated as severe (see Table 9.2).
- Hypotonic Hyporesponsive Episode (HHE) within 48 hours following vaccine administration.
- Seizures with or without fever within 3days following vaccine administration.

Subjects with a definitive contraindication will continue to be followed up for the study-defined safety and immunogenicity assessments, as applicable.

In the event of a local or national immunization program with OPV, subjects who receive OPV at any time during the study will not be withdrawn from the study.

5.2.8 Conditions for Withdrawal

Parent(s)/LAR will be informed that they have the right to withdraw their child from the study at any time.

A subject may be withdrawn from the study:

At the discretion of the Investigator or Sponsor due to safety concerns or significant non-compliance with the protocol (based on the Investigator's judgment), without the subject's permission (withdrawal)

At the request of the subject / parent / guardian / legally acceptable representative (dropout)

The reason for a withdrawal or dropout should be clearly documented in the source documents and on the CRB.

The Investigator must determine whether voluntary withdrawal is due to safety concerns (in which case, the reason for discontinuation will be noted as "Adverse Event") or for another reason.

Withdrawn subjects will not be replaced.

5.2.9 Lost to Follow-up Procedures

In the case of subjects who fail to return for a follow-up examination, documented reasonable effort (i.e., documented telephone calls and mail) should be undertaken to locate or recall them, or at least to determine their health status while fully respecting their rights. These efforts should be documented in the CRB and in the source documents.

5.2.10 Classification of Subjects Who Discontinue the Study

For any subject who discontinues the study prior to completion, the most significant reason for early termination will be checked in the CRB. Reasons are listed below from the most significant to the least significant (refer to the CRB completion instructions for additional details and examples):

**Adverse Event**	To be used when the subject is permanently terminated from the study because of an AE (including an SAE), as defined in Section 9.2.1.1.<br>This category also applies if the subject experiences a definitive contraindication that is an SAE or AE.
**Lost to Follow-up**	To be used when the subject cannot be found or contacted in spite of efforts to locate him/her before the date of his/her planned last visit, as outlined in Section 5.2.9. The certified letter was sent by the investigator and returned unsigned, and the subject or parent/guardian did not give any other news and did not come to any following visit.
**Protocol Deviation**	To be used:<br>In case of significant noncompliance with the protocol (e.g., deviation of the Inclusion / Exclusion criteria, non-compliance with time windows, blood sampling or vaccination refusal, missed injection/treatment, or error in the vaccine/treatment administration).<br>If the subject experiences a definitive contraindication that is a protocol deviation.<br>The subject or the parent/guardian signed the certified letter sent by the investigator but did not give any other news and did not come to any following visit.
**Withdrawal by Subject or Parent / Guardian / Legally Acceptable Representative**	To be used:<br>When the subject or parent/guardian indicated unwillingness to continue in the study<br>When the subject or parent/guardian made the decision to discontinue participation in the study for any personal reason other than an SAE/AE (e.g., subject is relocating, inform consent withdrawal, etc.)

5.2.11 Follow-up of Discontinuations

The site should complete all scheduled safety follow-ups and contact any subject who has prematurely terminated the study because of an AE, a protocol deviation, or loss of eligibility, including definitive contraindications.

For subjects where the reason for early termination was lost to follow-up or if the subject withdrew informed consent and specified that they do not want to be contacted again and it is documented in the source document, the site will not attempt to obtain further safety information.

5.3 Modification of the Study and Protocol

Any amendments to this study plan and protocol must be discussed with and approved by the Sponsor. If agreement is reached concerning the need for an amendment, it will be produced in writing by the Sponsor, and the amended version of the protocol will replace the earlier version. All substantial amendments (e.g., those that affect the conduct of the study or the safety of subjects) require IEC / IRB approval, and must also be forwarded to regulatory authorities.

An administrative / non-substantial amendment to a protocol is one that modifies some administrative, logistical, or other aspect of the study but does not affect its scientific quality or have an impact on the subjects' safety. The IECs / IRBs need only to be notified about administrative changes.

The Investigator is responsible for ensuring that changes to an approved study, during the period for which IEC / IRB approval has already been given, are not initiated without IEC / IRB review and approval, except to eliminate apparent immediate hazards to subjects.

5.4 Interruption of the Study

The study may be discontinued if new data about the investigational product resulting from this or any other studies become available; or for administrative reasons; or on advice of the Sponsor, the Investigators, the IECs/IRBs, or the governing regulatory authorities in India where the study is taking place.

If the study is prematurely terminated or suspended, the Sponsor shall promptly inform the Investigators, the IECs/IRBs, the regulatory authorities, and any contract research organization(s) used in the study of the reason for termination or suspension, as specified by the applicable regulatory requirements. The Investigator shall promptly inform the subjects' Parent(s)/LAR and should assure appropriate subject therapy and/or follow-up.

6 Vaccines Administered

6.1 Identity of the Investigational Product(s)

6.1.1 Identity of Study Product(s)

**Investigational Product:**	**SHAN 5®** (with Shantha pertussis and imported HBsAg)
Form:	Liquid, presented in 10-dose vial
Route:	Intramuscular injection into the anterolateral aspect of the thigh
Batch Number:	TBD

6.1.1.1 Composition

Each 0.5 mL dose of vaccine contains the following components:

Active ingredients:

Diphtheria Toxoid	≥ 30 IU
Tetanus Toxoid	≥ 60 IU
B. pertussis (Whole cell)	≥ 4 IU
HBsAg (rDNA)	10 µg
Purified capsular polysaccharide of Hib conjugated to 20-40 µg of Tetanus Toxoid (carrier protein)	10 µg

Other ingredients:

Preservative Thiomersal	0.050 mg
Sodium Chloride I.P	4.5 mg
Water for injection I.P.	q.s. to 0.5 mL

Adjuvant:

Aluminum Phosphate Gel equivalent to Al+++	0.625

6.1.1.2 Preparation and Administration

The vaccine should be gently shaken to obtain a homogenous suspension prior to the IM injection into the anterolateral area of the thigh. Only one dose (0.5 mL) of the vaccine in the vial will be administered to the corresponding subject. The remaining doses in the vial will remain unused. In other words, one separate vial will be used per subject per dose. Prior to administration, all study products must be inspected visually for cracks, broken seals, correct label content (see Section 6.3.1), and extraneous particulate matter and / or discoloration, whenever solution and container permit. If any of these conditions exists, the vaccine must not be administered. Another dose is to be used, and the event is to be reported to the Sponsor. Subjects must be kept under observation for 30 minutes after each vaccination to ensure their safety, and any reactions during this period will be documented in the CRB. Appropriate medical equipment and emergency medications, including epinephrine (1:1000), must be available on site in the event of an anaphylactic, vasovagal, or other immediate allergic reaction.

6.1.1.3 Dose Selection and Timing

0.5 mL of SHAN 5® (with Shantha pertussis and imported HBsAg) will be administered to all subjects randomized in Group-1 of Stage 1 at 6-8, 10-12 and 14-16 weeks of age and to all subjects in Stage 2 at 12-24 months of age.

6.1.2 Identity of Control Product(s)

**Control Product:**	**SHAN 5®** (with imported pertussis and Shantha HBsAg)
Form:	Liquid, presented in 10-dose vial
Route:	Intramuscular injection into the anterolateral aspect of the thigh
Batch Number:	PLU015A17 expiring in June 2019

6.1.2.1 Composition

Each 0.5 mL dose of vaccine contains:

Active ingredients:

Diphtheria Toxoid	≥ 30 IU
Tetanus Toxoid	≥ 60 IU
B. Pertussis(Whole cell)	≥ 4 IU
HBsAg(rDNA)	10 µg
Purified capsular polysaccharide of Hib conjugated to 20-40 µg of Tetanus Toxoid (carrier protein)	10 µg

Other ingredients:

Preservative Thiomersal	0.050 mg
Sodium Chloride I.P	4.5 mg
Water for injection I.P.	q.s. to 0.5 mL

Adjuvant:

Aluminum Phosphate Gel equivalent to Al+++	0.625

6.1.2.2 Preparation and Administration

The procedures for preparing and administering the control product are the same as those described for the study product in Section 6.1.1.2.

6.1.2.3 Dose Selection and Timing

0.5 mL of SHAN 5® (with imported pertussis and Shantha HBsAg) will be administered to all subjects randomized in Group-2 of Stage 1 at 6-8, 10-12 and 14-16 weeks of age.

6.2 Identity of Other Product(s)

6.2.1 Oral poliovirus vaccine (OPV):

The Bharat biotech vaccine (Biopolio® B1/3) will be used.

6.2.1.1 Composition

Composition of Biopolio® B1/3: contains suspension of live attenuated poliomyelitis type 1 and type 3 viruses (Sabin strain) prepared in Primary Monkey Kidney Cells. Each dose contains not less than $10^{6.0}$ $CCID_{50}$ virus concentration of type 1 strain and $10^{5.8}$ $CCID_{50}$ virus concentration of type 3 strain. Biopolio® B1/3 contains Magnesium chloride ($MgCl_2$) 1Molar as stabilizer; both Kanamycin acid and Neomycin sulphate as antibiotics.

6.2.1.2 Preparation and Administration

Refer to the manufacturer's package insert (25)

6.2.1.3 Dose Selection and Timing

OPV / IPV will be administered to all subjects randomized in Stage 1 at 6-8, 10-12 and 14-16 weeks of age and a booster dose will be administered to subjects in Stage 2 at 12-18 months of age.

6.2.2 Inactivated poliovirus vaccine (IPV):

The Shantha Biotechnics vaccine (SHANIPV™) will be used.

6.2.2.1 Composition

Composition of ShanIPV™:

Each 0.5 mL dose of vaccine contains the following components:

<u>Active ingredients:</u>

Poliomyelitis virus (Inactivated):

Type 1 (Mahoney strain)#	40 D antigen units
Type 2 (MEF-1 strain)#	8 D antigen units
Type 3 (Saukett strain)#	32 D antigen units

produced on VERO cells

Excipients: 2-phenoxyethanol, formaldehyde, ethanol, medium 199 Hanks (containing in particular amino acids, mineral salts, vitamins, glucose, polysorbate 80 and water for injections), hydrochloric acid or sodium hydroxide for pH adjustment.

6.2.2.2 Preparation and Administration

The SHANIPV™ will be administered by intramuscular injection into the anterolateral aspect of the thigh.

6.2.2.3 Dose Selection and Timing

OPV / IPV will be administered to all subjects randomized in Stage 1 at 6-8, 10-12 and 14-16 weeks of age and a booster dose will be administered to subjects in Stage 2 at 12-18 months of age.

6.2.3 Oral rotavirus vaccine (ORV):

The Bharat biotech vaccine (Rotavac®) or any locally available licensed ORV will be used.

6.2.3.1 Composition

Composition of Rotavac®:

Ingredients	Quantity / 0.5 mL
Rotavirus 116E Bulk, Live Attenuated	NLT $10^{5.0}$ FFU
Potassium Phosphate Monobasic IP	0.258 mg
Potassium Phosphate Dibasic IP	0.625 mg
Sucrose IP	37.31 mg
Potassium L-glutamate Monohydrate	1.0 mg
Neomycin Sulphate IP	15 µg
Kanamycin Sulphate IP	15 µg
Dulbecco's Modified Eagle's Medium	4.4 mg
Water for Injections IP	q.s

6.2.3.2 Preparation and Administration

Refer to the manufacturer's package insert (26)

6.2.3.3 Dose Selection and Timing

ORV will be administered to all subjects randomized in Stage 1 at 6-8, 10-12 and 14-16 weeks of age.

6.2.4 Measles Mumps Rubella (MMR) vaccine:

TRESIVAC™ (Serum Institute of India) or any locally available licensed MMR vaccine will be used.

6.2.4.1 Composition

The reconstituted vaccine contains in each single human dose of 0.5 ml:

Not less than 1000 CCID50 of Measles virus

Not less than 5000 CCID50 of Mumps virus

Not less than 1000 CCID50 of Rubella virus

Diluent : Sterile water for injection.

*CCID50 = cell culture infectious dose 50%

6.2.4.2 Preparation and Administration

Refer to the manufacturer's package insert (27)

6.2.4.3 Dose Selection and Timing

MMR (1st dose) will be administered to all subjects prior to Stage 2 at the recommended age of 9 months and MMR (2nd dose) will be administered to all subjects enrolled in Stage 2 at 15-16 months of age.

6.3 Product Logistics

6.3.1 Labeling and Packaging

Primary packaging

Each Multi dose vial of the SHAN 5® vaccine will be packed in an inner box (carton). Labelling will be done as per the applicable national regulatory guidelines. Information which has to be captured in the label includes but not limited to

- Sponsor's name and address
- Study code
- Name of the product
- Route of administration
- Subject enrollment number with the dose Number
- Batch number
- Manufacturing & Expiry date
- Storage condition (+2°C to +8°C)
- Directions for use
- The mention "For clinical trial use only"

- Name of the Investigator
- Name of the Institution

Secondary packaging:

Ten boxes of the investigational vaccines will be placed in bigger carton (outer carton), which will also be labelled. The labels for outer carton will contain the information (mentioned above) except for the space to capture subject specific details.

The concomitant vaccines (ShanIPV™, OPV, ORV and MMR) will be supplied with manufacturer's labeling and primary packaging. They will be provided to the site by the Sponsor.

6.3.2 Product Shipment, Storage, and Accountability

6.3.2.1 Product Shipment

The Clinical Program Manager (CPM) or designee will contact the Investigator or a designee to determine the dates and times of delivery of products.

Each vaccine shipment will include a temperature-monitoring device to verify maintenance of the cold chain during transit. On delivery of the product to the site, the person in charge of product receipt will follow the instructions given in the Operating Guidelines, including checking that the cold chain was maintained during shipment (i.e., verification of the temperature recorders). After verifying, the maintenance of cold chain during shipment, CPM or Sponsor designee will provide approval for the use of the study vaccines (Green light). If there is an indication that the cold chain was broken, the delegated site personnel should immediately quarantine the product, alert the Shantha representative, and request authorization from Shantha to use the product.

6.3.2.2 Product Storage

The Investigator will be personally responsible for product management or will designate a staff member to assume this responsibility.

At the site, products must be kept in a secure place with restricted access. Vaccines will be stored in a refrigerator at a temperature ranging from +2°C to +8°C and should be protected from light. The vaccines must not be frozen. The temperature must be monitored and documented (see the Operating Guidelines) for the entire time that the vaccine is at the study site. In case of accidental freezing or disruption of the cold chain, vaccines must not be administered and must be quarantined, and the Investigator or authorized designee should contact the Shantha representative for further instructions.

6.3.2.3 Product Accountability

The person in charge of product management at the site will maintain records of product delivery to the study site, product inventory at the site, the dose(s) given to each subject, and the disposal of or return to the Sponsor of unused doses.

The necessary information on the product labels is to be entered into the source document and the CRB. Information of vaccine administration should also be entered into the subject's vaccination card.

The Sponsor's monitoring staff will verify the study site's product accountability records against the record of administered doses in the CRBs.

In case of any expected or potential shortage of product during the study, the Investigator or an authorized designee should alert the Shantha representative as soon as possible, so that a shipment of extra doses can be arranged.

6.3.3 Replacement Doses

Each site will be provided with few extra doses of the vaccine, in order to tackle the situation where some doses cannot be used due to various reasons (e.g. spillage during administration or breakage of vial etc.). They may also follow the instructions given in the Operating Guidelines.

6.3.4 Disposal of Unused Products

Unused products will be either returned to the Sponsor in accordance with the instructions mentioned in the Operating Guidelines or handled by Investigator as per site's standard operating procedures for destruction of used Investigational product

Unused or wasted products will be either returned to the Sponsor in accordance with the instructions in the Operating Guidelines or handled by Investigator as per site's standard operating procedures for destruction of used Investigational product. Product accountability will be verified throughout the study period. Documentation of the same (destruction note) will be maintained in ISF as well as Trial master file (TMF).

6.3.5 Recall of Products

If the Sponsor makes a decision to launch a retrieval procedure, the Investigator(s) will be informed of what needs to be done.

6.4 Blinding and Code-breaking Procedures

This study will be observer-blind for Stage 1. Neither the observer Investigator, nor the Sponsor study staff interacting with the Investigator, nor the subjects will know which product will be administered. The "vaccinator" will be in charge of preparing and administering the products and will not be authorized to collect any safety data. In addition, the "vaccinator" or authorized designee will have to ensure that the documents on randomization are stored in a secure place where only he/she has access.

The code may be broken in the event of an AE only when the identification of the vaccine received could influence the treatment of the subject. Code-breaking should be limited to the subject(s) experiencing the AE.

The blind can be broken by the Investigator or a delegate, as explained in the code-breaking procedures described in the Operating Guidelines. Once the emergency has been addressed by the

site, the Investigator or a delegate must notify the Shantha Clinical Team Leader if a subject's code was broken. All contact attempts with the Sponsor prior to unblinding are to be documented in the source documents, and the code breaking CRF is to be completed.

A request for the code to be broken may also be made:

By the Sponsor's Global Pharmacovigilance (GPV) department. The information resulting from code-breaking (i.e. the subject's vaccine or group assignment) will not be communicated to either the Investigator or the immediate team working on the study, except for the GPV department.
The IEC / IRB must be notified of the code-breaking. All documentation pertaining to the event must be retained in the site's study records and in the Shantha files. Any intentional or unintentional code-breaking must be reported, documented, and explained, and the name of the person who requested it must be provided to the Sponsor.

6.5 Randomization and Allocation Procedures

After taking consent from the parent/LAR, the investigator will allot screening number[a] to each subject. The screening number will be an alpha-numerical consisting of 3-digit center identifier, 3-digit subject identifier and "SC" at the end. The 3-digit center identifier will be assigned to specific sites, before start of the trial. The 3-digit subject identifier corresponds to the chronological order of screening in the center (e.g., the 21st subject screened in center 1 will be allotted with a screening number: 001-021-SC.

After collection of first blood sample (BL1) at V01, the investigator will allocate an inclusion / enrollment number to each subject. The enrollment number will be a 6 digits string with a 3-digit center identifier, 3-digit subject identifier. As mentioned earlier, the 3-digit center identifier will be assigned to specific sites, before start of the trial. The 3-digit subject identifier corresponds to the chronological order of enrollment in the center and the Randomization number (e.g., the 21st subject included in center 1 will be subject: 001-021). Once the subject is enrolled, the enrollment number will remain the same in Stage 1 and Stage 2 i.e. subject with number 001-021 in Stage 1 will continue with the same number 001-021 in Stage 2 also.

Scratchable randomization list required for Stage 1 will be prepared by Sanofi Pasteur Biostatistics platform. The list will be created with a 1:1 ratio (iSHAN5 : cSHAN 5) using permuted blocks and will be stratified by center. Each subject enrollment number will correspond to one entry on the randomization sheet. At the end, the randomization sheet will contain information stating the name of the vaccine(s) (covered by a silver colored patch) allocated to the particular subject number, space for signature and date. Each entry in the randomization sheet will be signed and dated by the Investigator or the designee.. Each subject will be vaccinated with the product(s) corresponding to the group mentioned on the randomization list. If the dose initially taken for the vaccination is broken or cannot be used, the Investigator will take another dose of the same vaccine.

---

[a] ***The 3-digit center identifier of screening number and enrollment number will be same for a specific site, followed by 1-digit Stage identifier, whereas the 3-digit subject identifier of screening number and enrollment number may differ for a particular subject.***

Subject numbers should not be reassigned for any reason. If subject's parent(s)/LAR withdraw consent before collection of first blood sample (BL1), the subject will not be assigned with enrollment number and vaccination will not be performed.

Subjects in Stage 2 will not be randomized.

6.6 Treatment Compliance

The following measures will ensure that the vaccine doses administered comply with those planned, and that any non-compliance is documented so that it can be accounted for in the data analyses:

All vaccinations will be administered by qualified study personnel.

The person in charge of product management at the site will maintain accountability records of product delivery to the study site, product inventory at the site, dose(s) given to each subject, and the disposal of unused or wasted doses.

6.7 Concomitant Medications and Other Therapies

At the time of enrollment, ongoing medications and other therapies (e.g., blood products) should be recorded in the source document as well as new medications prescribed for new medical conditions / AEs during study participation.

Documentation in the CRB of ongoing concomitant medication(s) will be limited to specific categories of medication(s) of interest beginning on the day of first vaccination. This may include medications of interest that were started prior to the day of vaccination. Vaccines administered since birth in Stage 1 subjects will also be recorded.

Reportable medications will be collected in the CRB from the day of each vaccination to the end of the solicited and unsolicited follow-up period.

Reportable medications include medications that impact or may impact the consistency of the safety information collected after any vaccination and/or the immune response to vaccination. Three standard categories of reportable medications are defined:

Category 1: medications impacting or that may have an impact on the evaluation of the safety (e.g., antipyretics, analgesics, and non-steroidal anti-inflammatory drugs [NSAIDs], steroids/corticosteroids).

Category 2: medications impacting or that may have an impact on the immune response (e.g., other vaccines, blood products, antibiotic classes that may interfere with bioassays used by the Global Clinical Immunology [GCI] department, steroids/corticosteroids, immune-suppressors, immune-modulators with immunosuppressive properties, anti-proliferative drugs such as DNA synthesis inhibitors).

Category 3: medications impacting or that may have an impact on both the safety and the immune response (e.g., steroids/corticosteroids)

The information reported in the CRB for each reported medication will be limited to:

Trade name

Origin of prescription: prophylaxis Yes/No. Medication(s) prescribed for AE prophylaxis will be recorded in the Action Taken of the AE collection tables.

Medication category (1, 2, or 3)

Start and stop dates

Dosage and administration route, homeopathic medication, topical and inhaled steroids, as well as topical, ophthalmic, and ear treatments will not be recorded.

Medications given in response to an AE will be captured in the "Action Taken" section of the AE CRF only. No details will be recorded in the concomitant medication CRF unless the medication(s) received belongs to one of the prelisted categories. Medications will not be coded.

7 Management of Samples

Blood samples for the assessment of antibody responses will be collected at Visit(s) 1 and 4 in Stage 1 and at Visits 5 and 6 in Stage 2. See the Table of Study Procedures and Section 5.1.3 for details of the sampling schedule.

7.1 Sample Collection

At Visit(s) mentioned above, approx. 5 mL of blood will be collected in tubes provided by or recommended by the Sponsor. Immediately prior to the blood draw, the staff member performing the procedure will verify the subject's identity; will write the assigned subject's number on the pre-printed label that contains that subject's enrollment number and the sampling stage; and will attach the label to the tube. Blood is to be taken from the limb opposite to the one that will be used for vaccination.

7.2 Sample Preparation

Detailed instructions on how to prepare blood samples for assessment of immune response are contained in the Operating Guidelines provided to the site. An overview of the procedures is provided here.
Following the blood draw, the tubes are to be left undisturbed, positioned vertically, not shaken for a minimum of 60 minutes and not longer than the 2 hours, in order to allow the blood to clot. Samples can be stored at room temperature for up to 2 hour. Beyond that, they must be refrigerated at a temperature of +2°C to +8°C after the period of clotting at room temperature and must be centrifuged within a maximum of 24 hours to separate serum.
The samples are then centrifuged and the serum is transferred to the serum tubes. Centrifugation and aliquot preparation process is mentioned in detail in the Operating Guideline.
Sponsor's representative will coordinate to provide sample collection kits, serum tubes and the necessary labels to each site. The serum tubes should be properly labelled and along with other information, the sample code should also be entered on label.

The sample code will be the 8 digits string with a 3-digit center identifier, 3-digit subject identifier and 2-digit sampling visit identifier. The 3-digit center identifier will be assigned to specific sites,

before start of the trial. The 3-digit subject identifier corresponds to the chronological order of enrollment in the center. The 2 digit sampling visit identifier will denote whether the sample is pre-dose sample (01) or post dose sample (02) in stage 1 or pre-dose sample (03) or post dose sample (04) in stage 2. (e. g. the 11th subject of site 01's pre-dose blood sample in stage 1 will be coded as: 001-011-01 and post dose blood sample in stage 1 will be coded as: 001-011-02)

7.3 Sample Storage and Shipment

During storage, serum tubes are to be kept in a freezer whose temperature is set and maintained at -20°C or below. The temperature will be monitored and documented on the appropriate form during the entire study. If it rises above -10°C for any period of time, the the Clinical Program Manager (CPM)/ site monitor must be notified. See the Operating Guidelines for further details.

Shipments to the laboratories will be made only after appropriate monitoring, and following notification of the Clinical Program Manager (CPM)/ site monitor. Sera will be shipped frozen, using dry ice to maintain them in a frozen state, in the packaging container provided by the carrier. Again, temperatures will be monitored. Shipments must be compliant with the United Nations (UN) Class 6.2 specifications and the International Air Transport Association (IATA) 602 packaging instructions.

Samples will be shipped to Global Clinical Immunology (GCI) at Sanofi Pasteur. The address is provided in the Operating Guidelines.

7.4 Future Use of Stored Serum Samples for Research

Any unused part of the serum samples will be securely stored at the Sanofi Pasteur serology laboratory (GCI) for at least 5 years after the last license approval in the relevant market areas has been obtained for the vaccine being tested.

Subjects' parents / LAR will be asked to indicate in the ICF whether they will permit the future use of any unused stored serum samples for other tests. If they refuse permission, the samples will not be used for any testing other than that directly related to this study. If they agree to this use, they will not be paid for giving permission. Anonymity of samples will be ensured. The aim of any possible future research is unknown today, and may not be related to this particular study. It may be to improve the knowledge of vaccines or infectious diseases, or to improve existing tests or develop new tests to assess vaccines. Human genetic tests will never be performed on these samples without specific individual informed consent.

8 Clinical Supplies

Shantha will supply the study sites with protocols, ICFs, CRBs, diary cards, SAE reporting forms, and other study documents, as well as with the following study materials: all study vaccines, blood collection tubes, cryotubes, cryotube storage boxes, cryotube labels, temperature recorders, shipping containers, rulers, and digital thermometers.

The means for performing Electronic Data Capture (EDC) will be defined by Shantha. If a computer is provided by Shantha, it will be retrieved at the end of the study.

The Investigator will supply all vaccination supplies, phlebotomy, and centrifugation equipment, including biohazard and / or safety supplies. The biohazard and safety supplies include needles and syringes, examination gloves, etc. The site will ensure that all biohazard wastes are autoclaved and disposed of in accordance with local practices. The Investigator will also supply appropriate space in a temperature-monitored refrigerator for the storage of the products and for the blood samples, and appropriate space in a temperature-monitored freezer for serum aliquots.

In the event that additional supplies are required, study staff must contact Shantha, indicating the quantity required. Contact information is provided in the Operating Guidelines.

9 Endpoints and Assessment Methods

9.1 Primary Endpoints and Assessment Methods

9.1.1 Safety

There are no primary objectives for safety.

9.1.2 Immunogenicity

9.1.2.1 Immunogenicity Endpoints

The following serological primary endpoints will be assessed 28 days after the third dose of the primary series in Stage 1 for the non-inferiority analyses:

- Hep B seroprotection status defined as anti-HBs Ab concentration ≥10 mIU/mL
- Pertussis immune responses defined measured by geometric mean concentration adjusted on baseline concentration (aGMCs) for anti-PT and anti-FIM antibodies

9.1.2.2 Immunogenicity Assessment Methods

See section 9.2.2.2

9.1.3 Efficacy

No clinical efficacy data will be obtained in the study.

9.2 Secondary Endpoints and Assessment Methods

9.2.1 Safety

9.2.1.1 Safety Definitions

The following definitions are taken from the International Conference on Harmonization (ICH) E2A Guideline for Clinical Safety Data Management: Definitions and Standards for Expedited Reporting.

Adverse Event (AE):

An AE is any untoward medical occurrence in a patient or in a clinical investigation subject administered a medicinal product and which does not necessarily have a causal relationship with this treatment. An AE can therefore be any unfavorable and unintended sign (including an abnormal laboratory finding, for example), symptom or disease temporally associated with the use of a medicinal product, whether or not considered related to the medicinal product.

Therefore an AE may be:

A new illness

The worsening of a pre-existing condition

An effect of the vaccination, including the comparator

A combination of the above

All AEs include serious and non-serious AEs.

Surgical procedures are not AEs; they are the actions taken to treat a medical condition. It is the condition leading to the action taken that is the AE (if it occurs during the study period).

Pre-existing medical conditions are not to be reported as AEs. However, if a pre-existing medical condition worsens following study interventions in frequency or intensity, or if according to the Investigator there is a change in its clinical significance, this change should be reported as an AE (exacerbation). This applies equally to recurring episodes of pre-existing conditions (e.g., asthma) if the frequency or intensity increases post-vaccination.

Serious Adverse Event (SAE):

Serious and *severe* are not synonymous. The term *severe* is often used to describe the intensity of a specific event as corresponding to Grade 3. This is not the same as *serious* which is based on subject / event outcome or action criteria usually associated with events that pose a threat to a subject's life or functioning. Seriousness, not severity, serves as a guide for defining regulatory reporting obligations.

An SAE is any untoward medical occurrence that at any dose

Results in death

Is life-threatening[a]

Requires inpatient hospitalization or prolongation of existing hospitalization[b]

Results in persistent or significant disability / incapacity[c]

Is a congenital anomaly / birth defect

Is an important medical event (IME)

Medical and scientific judgment should be exercised in deciding whether expedited reporting is appropriate in other situations, such as IMEs that may not be immediately life-threatening or result in death or hospitalization but may jeopardize the health of the subject or may require intervention to prevent one of the other outcomes listed in the definition above. These IMEs should also usually be considered serious. Examples of such events include allergic bronchospasm requiring intensive treatment in an emergency room or at home, blood dyscrasias or convulsions that do not result in inpatient hospitalization, or the development of drug dependency or drug abuse, new-onset diabetes, or autoimmune disease.

Adverse Reaction:

All noxious and unintended responses to a medicinal product related to any dose should be considered adverse reactions (AR).

(The phrase "responses to a medicinal product" means that a causal relationship between a medicinal product and an AE is at least a reasonable possibility)

The following additional definitions are used by Shantha:

Immediate Event/Reaction:

Immediate events are recorded to capture medically relevant unsolicited systemic AEs (including those related to the product administered) that occur within the first 30 minutes after vaccination.

Solicited Reaction:

A solicited reaction is an "expected" adverse reaction (sign or symptom) observed and reported under the conditions (nature and onset) prelisted in the protocol and CRB (e.g., injection site pain occurring between D0 and D7 post-vaccination). By definition, solicited reactions are to be considered as being related to the product administered.

For injectable vaccines, solicited reactions can either be solicited injection site reactions or solicited systemic reactions.
For this trial:
Solicited injection site reactions include *(i) tenderness*; *(ii) erythema* and *(iii) swelling*

---

[a] The term "life-threatening" refers to an event in which the subject was at risk of death at the time of the event; it does not refer to an event which hypothetically might have caused death if it were more severe.

[b] All medical events leading to hospitalizations will be recorded and reported as SAEs, with the exception of: hospitalization planned before inclusion into the study or outpatient treatment with no hospitalization.

[c] "Persistent or significant disability or incapacity" means that there is a substantial disruption of a person's ability to carry out normal life functions.

Solicited systemic reactions include *(i) fever (ii) vomiting (iii) crying abnormal (iv) drowsiness (v) appetite lost and (vi) irritability*

The assessment of these reactions by the investigator is mandatory.

Unsolicited AE / AR:

An unsolicited AE is an observed AE that does not fulfill the conditions prelisted in the CRB in terms of diagnosis and/or onset window post-vaccination. For example, if fever between D0 and D7 is a solicited reaction (i.e., prelisted in the protocol and CRB), then a fever starting on D7 is a solicited reaction, whereas fever starting on D8 post-vaccination is an unsolicited AE. Unsolicited AEs includes both serious (SAEs) and non-serious unsolicited AEs.

Injection Site Reaction:

An injection site reaction is an AR at and around the injection site. Injection site reactions are commonly inflammatory reactions. They are considered to be related to the product administered.

Systemic AE:

Systemic AEs are all AEs that are not injection or administration site reactions. They therefore include systemic manifestations such as headache, fever, as well as localized or topical manifestations that are not associated with the vaccination or administration site (e.g., erythema that is localized but that is not occurring at the injection site).

Adverse Event of Special Interest (AESI):

An adverse event of special interest is one of scientific and medical concern specific to the Sponsor’s product or program, for which ongoing monitoring and rapid communication by the investigator to the sponsor can be appropriate. Such an event e.g., Anaphylaxis, HHE, Convulsions and Encephalopathy might warrant further investigation in order to characterize and understand it. Depending on the nature of the event, rapid communication by the study Sponsor to other parties (e.g., regulators) might also be warranted.

9.2.1.2 Safety Endpoints

The primary endpoint(s) for the evaluation of safety in Stages 1 and 2 are:

1) Occurrence of any unsolicited systemic AEs reported in the 30 minutes after each and after any study vaccine dose, as applicable.

2) Occurrence of solicited (i.e., pre-listed in the subject’s diary card [DC] and in the CRB), injection site reactions and systemic reactions occurring up to 7 days after each and any study vaccine dose, as applicable.

3) Occurrence of unsolicited (spontaneously reported) AEs up to 28 days after each and any study vaccine dose, as applicable.

4) Occurrence of SAEs (including AESIs), throughout the trial period.

Other endpoints recorded or derived as described in the statistical analysis plan. Depending on the item, these could include: nature (Medical Dictionary for Regulatory Activity [MedDRA] preferred term), time of onset, duration, number of days of occurrence, Grade of intensity, relationship to

vaccine, action taken, whether the AE led to early termination from the study, seriousness, or outcome.

9.2.1.3 Safety Assessment Methods

At each visit, the Investigator or a delegate will perform a clinical or medically-driven physical examination and will ask the subject's parent/LAR about any solicited reactions and unsolicited AEs recorded in the diary card, as well as about any other AEs that may have occurred since the previous visit. All relevant data will be transcribed into the CRB according to the instructions provided by the Sponsor.

9.2.1.3.1 Immediate Post-vaccination Observation Period

Subjects will be kept under observation for 30 minutes after each vaccination to ensure their safety. The post-vaccination observation should be documented in the source document. Any AE that occurs during this period will be noted on the source document and recorded in the CRB, as follows:

Unsolicited systemic AEs will be recorded as immediate AEs in the CRB (presence marked as "yes" and details collected).

Solicited and unsolicited injection site reactions and solicited systemic reactions will be recorded in the CRB in the same way as any reactions starting on the day of vaccination.

SAEs will be recorded in the CRB and reported to the Sponsor in the same way as any other SAEs, according to the procedures described in Section 10.

9.2.1.3.2 Reactogenicity (Solicited Reactions From Day 0 to Day 7 After Each Vaccination)

After each vaccination, subjects' parents / LAR will be provided with a diary card, a digital thermometer, and a flexible ruler, and will be instructed how to use them. The following items will be recorded by the subjects in the diary card on the day of vaccination and for the next 7 days (i.e., D0 to D7) until resolution:

Daily temperature, with the route by which it was taken

Daily measurement or intensity grade of all other solicited injection site and systemic reactions

Action taken for each event (e.g., medication)

The action(s) taken by the subject's parent / LARto treat and/or manage any **solicited reactions** will be classified in the CRB using the following list (all applicable items should be checked):

- None
- Medication
- Health care provider contact
- Hospitalized
- Discontinuation of study vaccination

Subjects' parents / LAR will be contacted by telephone8days after each vaccination to remind them to record all safety information in the diary card.

If the timing of the telephone call should fall on a weekend or a holiday, the call should be made on the next business day. If contact is not made on the designated day, study staff will continue calling until contact is made. Every telephone attempt and its outcome will be documented in the source document.

Table 9.1 and Table 9.2 present, respectively, the injection site reactions and systemic reactions that are prelisted in the diary cards and CRB, together with the intensity scales.

Table 9.1: Solicited injection site reactions: terminology, definitions, and intensity scales

**CRB term (MedDRA lowest level term [LLT])**	**Injection site tenderness**	**Injection site erythema**	**Injection site swelling**
**Data analysis term (MedDRA preferred term [PT])**	Injection site pain	Injection site erythema	Injection site swelling
**Diary card term**	Tenderness	Redness	Swelling
**Definition**	Pain when the injection site is touched or injected limb mobilized	Presence of a redness including the approximate point of needle entry	Swelling at or near the injection site<br>Swelling or edema is caused by a fluid infiltration in tissue or cavity and, depending on the space available for the fluid to disperse, swelling may be either soft (typically) or firm (less typical) to touch and thus can be best described by looking at the size of the swelling
**Intensity scale***	Grade 1: Minor reaction when injection site is touched<br>Grade 2: Cries or protests when injection site is touched<br>Grade 3: Cries when injected limb is mobilized, or the movement of the injected limb is reduced	Grade 1: > 0 to < 25 mm<br>Grade 2: ≥ 25 to < 50 mm<br>Grade 3: ≥ 50 mm	Grade 1: > 0 to < 25 mm<br>Grade 2: ≥ 25 to < 50 mm<br>Grade 3: ≥ 50 mm

* For the subjective reaction of tenderness, subjects' parents / LARwill record the intensity level (Grade 1, 2, or 3) in the diary card. For the measurable reactions of redness and swelling, they will record just the size of the reaction, and the classification as Grade 1, 2, or 3 will be assigned at the time of the statistical analysis.

Table 9.2: Solicited systemic reactions: terminology, definitions, and intensity scales

**CRB term (MedDRA lowest level term [LLT])**	**Fever**	**Vomiting**	**Crying abnormal**	**Drowsiness**	**Appetite lost**	**Irritability**
**Data analysis term (MedDRA preferred term [PT])**	Pyrexia	Vomiting	Crying	Somnolence	Decreased appetite	Irritability
**Diary card term**	Temperature	Vomiting	Abnormal crying	Drowsiness	Loss of appetite	Irritability
**Definition**	Elevation of temperature to ≥°38.0°C (≥ 100.4°F)	Vomiting does not include spitting up	Inconsolable crying without a determined reason	Reduced interest in surroundings, or increased sleeping	See intensity scale	An excessive response to stimuli: increased fussiness, whining, and fretfulness despite attempts to comfort the infant and despite caregiver responses that would normally be soothing
**Intensity scale***	Grade 1: ≥ 38.0°C to ≤ 38.5°C **or** ≥ 100.4°F to ≤ 101.3°F	Grade 1: 1 episode per 24 hours	Grade 1: < 1 hour	Grade 1: Sleepier than usual or less interested in surroundings	Grade 1: Eating less than normal	Grade 1: Easily consolable
	Grade 2: > 38.5°C to ≤ 39.5°C **or** > 101.3°F to ≤ 103.1°F	Grade 2: 2–5 episodes per 24 hours	Grade 2: 1–3 hours	Grade 2: Not interested in surroundings or did not wake up for a feed / meal	Grade 2: Missed 1 or 2 feeds / meals completely	Grade 2: Requiring increased attention
	Grade 3: > 39.5°C **or** > 103.1°F	Grade 3: ≥ 6 episodes per 24 hours or requiring parenteral hydration	Grade 3: > 3 hours	Grade 3: Sleeping most of the time or difficult to wake up	Grade 3: Refuses ≥ 3 feeds / meals or refuses most feeds / meals	Grade 3: Inconsolable

* For all reactions but fever, subjects' parents / LAR will record the intensity level (Grade 1, 2, or 3) in the diary card. For fever, they will record the body temperature, and the classification as Grade 1, 2, or 3 will be assigned at the time of the statistical analysis based on the unit used to measure the temperature and the intensity scale.

Important notes for the accurate assessment of temperature:

Subjects' parents / LARare to measure body temperature once per day, preferably always at the same time. The optimal time for measurement is the evening, when body temperature is the highest. Temperature is also to be measured at the time of any apparent fever. The observed daily temperature and the route of measurement are to be recorded in the DC and the highest temperature will be recorded by the site in the CRB. The preferred route for this study is axillary. Pre-vaccination temperature is also systematically collected by the investigator on the source document. Tympanic thermometers must not be used.

9.2.1.3.3 Unsolicited Adverse Events

In addition to recording solicited reactions, subjects' parents / LAR will be instructed to record any other medical events that may occur during the 28 day period after each vaccination. Space will be provided in the diary card for this purpose.

For each unsolicited AE (whether serious or non-serious), the following information is to be recorded:

Start and stop dates[a]

Intensity of the event:

For measurable unsolicited AEs that are part of the list of solicited reactions, the size of the AE as well as the temperature for fever will be collected and analyzed based on the corresponding scale used for solicited reactions (see Table 9.1 and Table 9.2).

All other unsolicited AEs will be classified according to the following intensity scale:

- Grade 1: A type of adverse event that is usually transient and may require only minimal treatment or therapeutic intervention. The event does not generally interfere with usual activities of daily living.
- Grade 2: A type of adverse event that is usually alleviated with additional therapeutic intervention. The event interferes with usual activities of daily living, causing discomfort but poses no significant or permanent risk of harm to the research participant.
- Grade 3: A type of adverse event that interrupts usual activities of daily living, or significantly affects clinical status, or may require intensive therapeutic intervention.

- Whether the AE was related to the investigational product (for unsolicited systemic AEs)

  The Investigator will assess the causal relationship between the AE and the investigational product as either "Not related" or "Related", as described in Section 9.2.1.3.6.

- Action taken for each AE (e.g., medication)

[a] The stop date of all related AEs will be actively solicited. For other events, the investigator will provide the stop date when it becomes available. AEs for which no stop date was obtained during the course of the study will be considered as ongoing at the end of the study.

The action(s) taken by the subjects' parents / LAR to treat and/or manage any unsolicited AEs will be classified in the CRB using the following list (all applicable items should be checked):

- None
- Medication
- Health care provider contact
- Hospitalized
- Discontinuation of study vaccination

Whether the AE was serious

For each SAE, the investigator will complete all seriousness criteria that apply (outcome, elapsed time, and relationship to study procedures)

Whether the AE caused study discontinuation

9.2.1.3.4 Serious Adverse Events

For Stage 1 and Stage 2, information on SAEs will be collected and assessed throughout the study, from screening on D0 until 28 days after the last vaccination. Any SAE occurring at any time during the study will be reported by the Investigator using both Serious Adverse Event Reporting Form and through the EDC system according to electronic case report form (eCRF) completion guideline provided by the Sponsor. All information concerning the SAE is to be reported either as part of the initial reporting or during follow-up reporting if relevant information became available later (e.g., outcome, medical history, results of investigations, copy of hospitalization reports). In case a subject experiences febrile convulsion (neurological event associating fever and seizure), the assessment will be performed according to the "Guideline for definition and collection of cases of febrile convulsion", and this event will be considered an SAE. See Section 10 for further details on SAE reporting.

9.2.1.3.5 Adverse Events of Special Interest

The following AESIs have been defined based upon the prior experience with the use of the marketed pentavalent combination vaccine

- Anaphylaxis/hypersensitivity
- Convulsions, including febrile convulsion
- Hypotonic Hyporesponsive Episode (HHE)
- Encephalopathy

The standard case definitions from Brighton Collaboration will be used for anaphylaxis (28), convulsions (29), and HHE (30) (31), and Encephalopathy. (32)

These AESIs are to be considered as SAEs and reported to the Sponsor between D0 and D84 for Stage 1 and D0 and D28 for Stage 2.

9.2.1.3.6 Assessment of Causality

The Investigator will assess the ***causal relationship*** between each unsolicited systemic AE and the product administered as either ***not related*** or ***related***, based on the following definitions:

Not related – The AE is clearly / most probably caused by other etiologies such as an underlying condition, therapeutic intervention, or concomitant therapy; or the delay between vaccination and the onset of the AE is incompatible with a causal relationship; or the AE started before the first vaccination (screening phase, if applicable)

Related – There is a “reasonable possibility” that the AE was caused by the product administered, meaning that there is evidence or arguments to suggest a causal relationship

Note: By convention, all AEs reported at the injection site (whether solicited or unsolicited) and all solicited systemic AEs are considered to be related to the administered product and therefore are referred to as reactions and do not require the Investigator’s opinion on relatedness.

Adverse events likely to be related to the product, whether serious or not, that persist at the end of the study will be followed up by the Investigator until their complete disappearance or the stabilization of the subject’s condition. The Investigator will inform the Sponsor of the date of final disappearance of the event or the date of “chronicity” establishment.

9.2.2 Immunogenicity

9.2.2.1 Immunogenicity Endpoints

For Stage 1 and 2: The following serological endpoints will be assessed at baseline D0 and 28 days after the third dose of the primary series in stage 1 and at baseline D0 and 28 days after the booster dose in stage 2:

1. Antibody concentration above the following cut-off for each valence
   - Anti-D antibody concentration ≥ 0.01 IU/mL, ≥ 0.1 IU/mL and ≥ 1.0 IU/mL
   - Anti-T antibody concentration ≥ 0.01 IU/≥ 0.1 IU/mL and ≥ 1.0 IU/mL
   - Anti-HBs antibody concentration ≥ 10 mIU/mL and ≥ 100 mIU/mL
   - Anti-PRP antibody concentration ≥ 0.15 mcg/mL and ≥ 1.0 mcg/mL
2. Antibody concentration for each valence at each time point
3. Pertussis antigens vaccine response status for anti-PT, anti-FHA, anti-PRN and anti-FIM antibodies defined as follows: subject with post-dose 3 vaccination concentration ≥ 4xLLOQ[¥] if the pre-vaccination concentration is <4xLLOQ or with post-Dose 3 vaccination concentration ≥ the pre-vaccination concentration if the pre-vaccination concentration is ≥4xLLOQ

[¥]according to MesoScale Discovery Immunoassay (MSD) Multiplexed Electro Chemiluminescent Method (MSD-ECL)

4. Pertussis antigens vaccine seroconversion status for anti-PT, anti-FHA, anti-PRN and anti-FIM antibodies defined as follows: subject with a ≥ 4-fold rise in their respective PT, FHA, PRN, FIM antibody concentration between pre-Dose 1 and post-Dose 3
5. Ratio (post dose 3 /pre-primary; post booster /pre-booster) of individual Ab concentration for all Abs

9.2.2.2 Immunogenicity Assessment Methods

All immunological assays will be carried out at the Central Lab (GCI Swiftwater, Pennsylvania, USA).
The assays will be performed on BL1 and BL2 samples obtained from all the subjects in Stage 1 and on BL3 and BL4 obtained from all the subjects in Stage 2. The assay methods to be used are summarized below:
MSD-ECL: The MSD-ECL method is a multiplexed serological assay, which allows for the simultaneous quantification of human IgG against six specific antigens including *Corynebacterium diphtheriae* Toxoid, *Clostridium tetani* Toxoid, and four *Bordetella pertussis* antigens: Filamentous Hemagglutinin (FHA), Pertussis Toxin (PT), Fimbriae Types 2 & 3 (FIM) and Pertactin 69kD (PRN). In this assay, each well of a 96 well SECTOR plate is coated in precise positions with the six different antigens listed above by the manufacturer. Following incubation with serum samples, antigen-specific antibodies bind to the respective antigens. The captured antibodies are then detected using a SULFO-TAG conjugated anti-human IgG conjugate. Electrical stimulation of the conjugate in the presence of a chemiluminescent substrate results in the generation of a light signal from each specific spot that is captured by a camera in relative light units (RLU). The signal thus generated is directly proportional to the amount of antibodies present in the sample, which are quantified by direct comparison to a four parameter logistic (4PL) reference standard using SoftMax® Pro data analysis software package.
Amongst all *B. pertussis* antigens, PT and FIM play important roles. PT is the main driver of the pathogenicity of an active *B. pertussis* infection and anti-PT antibodies are the main driver of the capacity of vaccinees to limit clinical symptoms, if colonized. (33) (34) Anti-PT responses are recommended to be documented as per WHO Technical Report Series for evaluation of wP vaccines serological responses. (35) Most of the *B. pertussis* agglutinating activities measured in sera of wP-vaccinated subjects are mediated by anti-FIM antibodies. (36) (37) (38) *B. pertussis* agglutination assay has been the gold standard assay to assess wP vaccines responses in the past. (39) WHO recommends that wP vaccines must contain FIM2 and FIM3 antigens. (35)

Anti-hepatitis B antibodies will be measured by the commercially available VITROS ECi/ECiQ Immunodiagnostic System using chemiluminescence detection technology. The VITROS ECi Immunodiagnostic system uses an antibody mediated antigen sandwich formation to detect the presence of anti-HBsAg total immunoglobulin in human serum. This involves the reaction of anti-HBsAg in the sample with plasma-derived HBsAg (ad and ay subtypes) coated onto the wells. A horseradish peroxidase (HRP)-labeled HBsAg conjugate (ad and ay subtypes) then complexes with the bound anti-HBs, forming an antigen sandwich. Substrate is then added which catalyzes HRP, producing light. The light signals are read by the VITROS ECi/ECiQ Immunodiagnostic System and the amount of HRP conjugate bound is directly proportional to the concentration of anti-HBs antibodies present in the sample. Results are reported in mIU/mL by comparison to a calibrator provided by the manufacturer that was previously calibrated to the WHO First International Reference Preparation for Antibody to HBsAg (1977).

Serum levels of anti-*Haemophilus influenzae* type b (Hib) PRP antibodies are determined by RIA, in which serum samples are incubated with radiolabeled PRP. Specific antibodies bind to antigen labeled with titrated capsular polysaccharide (^{3}H-PRP) to form antigen-antibody complexes. These complexes are precipitated with ammonium sulfate and collected by centrifugation. The radioactivity measured in the precipitated pellet, in counts per minute (CPM), is proportional to the

amount of anti-PRP antibody present in the serum sample. The concentration of anti-PRP antibody in the serum sample is determined from the concentration response curve generated by the titration results of dilutions of the reference standard analyzed in the assay. Results will be reported in micrograms (μg)/mL by comparison to Center for Biologics Evaluation and Research (CBER), Lot No. 1983 reference standard. The LLOQ for the anti-PRP assay was 0.06 μg/mL. Samples calculated to a value less than 0.06 were reported as <0.06 μg/mL.

All the assays will be set up and validated at the laboratory prior to running the clinical samples.

In case of insufficient sample volume, the priority of assays for testing is as follows:

1. MSD-ECL for anti-PT, FHA, PRN, FIM, Dip and Tet
2. Vitros-ECi for anti-Hepatitis B
3. RIA for anti-PRP

No antibody determinations will be made for poliovirus neutralizing antibodies, for rotavirus antibodies, measles, mumps and rubella antibodies.

9.2.3 Efficacy

No clinical efficacy data will be obtained in the study.

9.3 Observational Endpoints and Assessment Methods

There are no observational objectives in this study.

10 Reporting of Serious Adverse Events

To comply with current regulations on SAE reporting to health authorities, the Investigator must document all SAEs regardless of causal relationship, and notify the Sponsor and the Clinical Research Associate (CRA) within the notification timelines stated in the following sections. The Investigator will give access and provide the Sponsor and the CRA with all necessary information to allow the Sponsor to conduct a detailed analysis of the safety of the investigational product(s). It is the responsibility of the Investigator to request all necessary documentation (e.g., medical records, discharge summary) in order to provide comprehensive safety information. All relevant information must then be transcribed onto the AE CRF and the appropriate Death/Safety Complementary Information CRFs.

10.1 Initial Reporting by the Investigator

Serious adverse events occurring during a subject's participation in the study or experiment must be reported within 24 hours to the Sponsor's GPV Department, Chairman of the respective Ethics Committee and licensing authority (DCGI) and to the CRA. Every SAE must be reported, even if the Investigator considers that it is not related to the vaccine. The investigator (licensed physician

[M.D. or D.O.]) must validate the information entered on the AE eCRF by completing the investigator validation form.

The Investigator must indicate on the AE eCRF that the event was serious and must complete the relevant SAE section of this form as well as the appropriate Death/Safety Complementary Information CRFs. An e-mail alert will automatically be sent by the EDC system to the Shantha GPV mailbox, Sanofi India PV mailbox, the CRA and the Clinical Team Leader (CTL) with relevant SAE information details.
Once the accuracy of the information is validated the investigator should print a copy of eSAE form and it must be signed by Investigator or Co-Investigator. This form should be submitted to the licensing authority (DCGI).

If the EDC system is unavailable, the site must notify the Sponsor, using the paper version of the CRB, as described in the operating guidelines:
The Investigator must complete the SAE Reporting Form, check off the "Initial Reporting Form" box, and send it to the Sponsor by one of the following means:

- By fax, to the following number:[redacted], +[redacted] (Attention [redacted]
- By email, to the following e-mail address: Pharmacovigilance.Shantha@sanofi.com & CC to [redacted] PV.outsourcing@sanofi.com

When the system becomes available, the Investigator must transcribe the same into the EDC system.

If there is need for urgent consultation, the Investigator is to contact the Sponsor's Global Product Safety Officer (GSO; [redacted]) at [redacted]

When the EDC system becomes available, the Investigator must transcribe the information from the paper forms into the EDC system.

10.2 Follow-up Reporting by the Investigator

After the initial report, a SAE follow up report must be submitted by the Investigator, within 14 calendar days to the Sponsor's PV department, Chairman of the respective Ethics Committee, Head of the institution and licensing authority (DCGI) through paper version of the SAE Reporting Form checked with "Follow-up Reporting Form". The SAE form must be signed by Investigator or Co-Investigator. The follow-up form is to be sent by fax or through email as described above for the initial form. In any case, the AE eCRF completed initially must be updated within 24 hours after the Investigator has become aware of any new relevant information concerning the SAE (e.g., outcome, precise description of medical history, results of the investigation). All relevant information must be included directly in the AE eCRF. An e-mail alert will be sent automatically to the Shantha GPV mailbox, Sanofi India PV mailbox, the Clinical Team Leader (CTL) and to the CRA. Copies of documents (e.g., medical records, discharge summary, autopsy) may be requested by the GPV Department.

The anonymity of the subject must always be respected when forwarding this information.

After communication of SAE to the site ethics committee, Investigator of the site should follow-up with their respective IEC/IRB to prepare their opinion report (including causality) on the SAE along

with a recommendation on any financial/medical compensation deemed necessary, to licensing authority (DCGI) within 30 calendar days. The investigator should share the communication to Sponsor.

10.3 Reporting of SAEs Occurring After a Subject Has Completed the Study

Any SAE that occurs after a subject has completed the study but that is likely to be related to the investigational product(s), other products (e.g., a concomitant vaccine), or to the experiment must also be reported as soon as possible. In such a case, the reporting procedure to be followed is identical to that described in Section 10.1.

10.4 Assessment of Causality

The causal relationship between the SAE and the product administered will be evaluated by the Investigator as described in Section 9.2.1.3.6.

Following this, the Sponsor's Pharmacovigilance (PV) Global Safety Expert will also assess the causal relationship to the product, based on the available information and current medical knowledge.

The causal relationship to study procedures will be also assessed in the CRB.

The decision to modify or discontinue the study may be made after mutual agreement between the Sponsor and the Investigator(s).

10.5 Reporting SAEs to Health Authorities and IECs / IRBs

The Sponsor will inform the relevant health authorities of any reportable SAEs according to the local regulatory requirements. Reporting to the health authorities will be according to the Sponsor's standard operating procedures.

The Sponsor's Clinical Team Leader will notify the Investigators in writing of the occurrence of any reportable SAEs. The Investigators will be responsible for informing the IECs or IRBs that reviewed the study protocol.

11 Data Collection and Management

11.1 Data Collection and CRB Completion

Individual diary cards, specifically designed for this study by the Sponsor and provided to the study sites, will be given to study participants for the recording of daily safety information as described in Section 9.2.1.3. These diary cards will include prelisted terms and intensity scales (see Table 9.1 and Table 9.2) as well as areas for free text to capture additional safety information or other relevant details. Subjects' parents / LAR will also be provided with rulers for measuring the size of injection site reactions, and with standard digital thermometers for measuring daily temperatures. To ensure

consistency of reporting, the study sites will instruct subjects' parents / LAR on how to correctly use these tools.

At specified intervals, the Investigator or an authorized designee will interview the subjects' parents / LAR to collect the information recorded in the diary card, and will attempt to clarify anything that is incomplete or unclear. All clinical study information gathered by the study site will be reported electronically by the Investigator or authorized designee using a web-based CRB. (Any information that was not documented in the diary card will first be captured in the source document and then reported electronically.) The CRB has been designed specifically for this study under the responsibility of the Sponsor, using a validated Electronic Records / Electronic Signature-compliant platform (21 CFR Part 11).

To ensure the correct and consistent completion of the CRBs, the Sponsor or authorized representative will provide all necessary tools, instructions, and training to all site staff involved in data entry prior to study start. Additional instructional documents such as training manuals and completion instructions will be provided to assist with data entry during the course of the study.

Upon completion of training, each user requiring access to the EDC system will be issued a unique username and password. In the event of a change in study personnel, each newly assigned individual will receive a unique username and password; the username and password of a previous user may not be reissued. If any study personnel leave the study, the Investigator is responsible for informing the Sponsor immediately so that their access is deactivated. An audit trail will be initiated in the EDC system at the time of the first data entry to track all modifications and ensure database integrity.

The Investigator is responsible for the timeliness, completeness, and accuracy of the information in the CRBs; must provide explanations for all missing information; and must sign the CRB using an e-signature.

11.2 Data Management

Management of SAE Data

During the study, SAE data (reported on the AE, Death, and Safety Complementary Information CRFs) will be integrated into the Sponsor's centralized GPV database upon receipt of these forms and after a duplicate check. Each case will be assigned a case identification number. Each case will be assessed by the case management platform or its delegate before being reported to the relevant authorities as necessary. The assessment of related cases will be done in collaboration with the PV Global Safety Expert and the CTL. Follow-up information concerning a completed case will be entered into the GPV database, and a new version of the case will be created.

The information from the GPV database cases will be reconciled with that in the clinical database.

Management of Clinical and Laboratory Data

Clinical data, defined as all data reported in the CRB, and laboratory data will be handled by the Sponsor's Clinical Data Management (CDM) platform or authorized representative.

During the study, clinical data reported in the CRBs will be integrated into the clinical database under the responsibility of the Sanofi Pasteur CDM platform. Data monitoring at the sites and quality control in the form of computerized logic and / or consistency checks will be systematically applied to detect errors or omissions. In addition, data reviews may be performed several times by the Sponsor's staff in the course of the study. Any questions pertaining to the reported clinical data will be submitted to the investigator for resolution using the EDC system. Each step of this process will be monitored through the implementation of individual passwords to maintain appropriate database access and to ensure database integrity.

The validation of the immunogenicity data will be performed at the laboratory level following the laboratory's procedures. Information from the laboratory will be checked for consistency before integration into the clinical Datawarehouse.

After integration of all corrections in the complete set of data, and after the SAE information available from CDM and the GPV Department has been reconciled, the database will be released for statistical analysis.

11.3 Data Review

A review of the data is anticipated through the data review process led by Data Management before database lock.

12 Statistical Methods and Determination of Sample Size

12.1 Statistical Methods

The statistical analyses will be carried out under the responsibility of the SP's Biostatistics platform, with the SAS® software, version 9.4 or above (SAS Institute, Cary, NC, USA). SAEs will be managed by the Sponsor's PV department. A detailed statistical analysis plan (SAP) will be written and validated before the database lock. In accordance with the protocol, the SAP will describe all the analyses to be performed, statistical tables and listings including descriptions of the analysis conventions to be used.

12.1.1 Hypotheses and Statistical Methods for Primary Objective(s)

12.1.1.1 Hypotheses

The primary objective is to demonstrate non-inferiority of investigational SHAN 5® compared to licensed SHAN 5® at D84, ie, 28 days after the third vaccine injection on Hep B and Pertussis immunogenicity responses, based on the following hypotheses:

For Hepatitis B:

- H0: $P_{iSHAN\ 5} - P_{cSHAN\ 5} \leq -10\%$
- H1: $P_{iSHAN\ 5} - P_{cSHAN\ 5} > -10\%$

With P = proportion of subjects with an anti-HBs Ab concentration ≥10 mIU/mL at D84 (%).

For Pertussis (on PT and FIM):

- H0: $aGMC_{iSHAN\ 5} / aGMC_{cSHAN\ 5} \leq 1/2$
- H1: $aGMC_{iSHAN\ 5} / aGMC_{cSHAN\ 5} > 1/2$

12.1.1.2 Statistical Methods

For HepB, non-inferiority testing will be based on the use of the two-sided 95% confidence interval (CI) of the difference of proportions of subjects with an anti-HBs Ab concentration ≥10 mIU/mL at D84. The 95% CI for differences will be calculated using Wilson score method without continuity correction.

Hepatitis B non-inferiority will be demonstrated if the lower limit of the 95% CI of the difference of the two proportions $P_{iSHAN\ 5} - P_{cSHAN\ 5}$ is > -10%, meaning that the null hypothesis H0 is rejected.

For Pertussis, non-inferiority testing will be based on the use of aGMCs and their 95%CI, on both PT and FIM results.

Adjusted GMCs will be computed using analysis of covariance to adjust for baseline disparities and consider the correlation between pre- and post-vaccination concentration (log-transformed), through an ANCOVA model using the pre-vaccination (D0) concentration as a covariate for adjustment in order to account for the associated variability. The 95% CI of ratio between aGMCs will be calculated using normal approximation of the log10 of the concentration.

Pertussis on-inferiority will be demonstrated if the lower limits of the 95% CI of the ratio of adjusted GMCs for PT and for FIM antigens are > 0.5.

Overall non-inferiority will be demonstrated if the Pertussis (PT and FIM) and Hepatitis B non-inferiority are demonstrated.

12.1.2 Hypotheses and Statistical Methods for Secondary Objective(s)

12.1.2.1 Hypotheses

All analyses will be descriptive; no hypotheses will be tested.

12.1.2.2 Statistical Methods

Descriptive statistics will be produced for each secondary endpoint.

The main safety and immunogenicity parameters will be described with 95% CI.

Immunogenicity endpoints will be summarized by vaccine group and by time-point (pre and post primary series vaccination and pre and post booster vaccination).

The following parameters will be used for anti-D, anti-T, anti-Hep B, anti- Pertussis and anti-Hib antibody concentration:

- Percentage of subjects with concentration above predefined cut-off (as per secondary endpoints)
- Percentage of subjects with ≥4 fold rise in anti-pertussis antibody concentration and / or vaccine response
- Geometric mean ratio (GMCR) of individual Ab concentration
- GMC for each valence, adjusted GMCs for PT, FIM, PRN and FHA

For descriptive immunogenicity analyses, geometric mean concentrations (GMCs), adjusted GMCs (for Pertussis endpoints) and geometric mean concentration ratio (GMCR) will be calculated with their 95% CI (normal approximation method).

Reverse Cumulative Distribution Curves (RCDCs) of individual concentration will be presented for the two groups.

For descriptive safety analyses, percentages will be presented with their 95% CI (Clopper-Pearson method).

12.2 Analysis Sets

12.2.1 Full Analysis Set

The full analysis set (FAS) is defined for each stage of the trial as the subset of enrolled subjects who received at least 1 dose of the study vaccine as defined in the corresponding stage.

12.2.2 Safety Analysis Set

The safety analysis set (SafAS) is defined for each stage of the trial as those subjects who have received at least 1 dose of the study vaccines[a] as defined in the corresponding stage. All subjects will have their safety analyzed after each dose according to the vaccine they actually received and after any dose according to the vaccine received at the first dose (for Stage 1) and after the booster dose overall.
Safety data recorded for a vaccine received out of the protocol design will be excluded from the analysis (and listed separately).
The analysis will be based upon the number of subjects for whom each safety assessment was performed. The number of subjects used to analyze safety measurements may therefore differ between the various safety assessments.

[a] for which safety data are scheduled to be collected

12.2.3 Per-Protocol Analysis Set

The per-protocol analysis set (PPAS) is a subset of the FAS and is defined for Stage 1 only. The subjects presenting with at least one of the following relevant protocol deviations will be excluded from the PPAS:

- Subject did not meet all protocol-specified inclusion criteria or met at least one of the protocol-specified exclusion criteria
- Subject did not complete the vaccination schedule
- Subject received a vaccine other than the one that he / she was randomized to receive
- Preparation and / or administration of vaccine was not done as per-protocol
- Subject did not receive vaccine in the proper time window:
    - Vaccination 2 outside the 28-35 days range since vaccination 1
    - Vaccination 3 outside the 28-35 days range since vaccination 2
- Subject did not provide a post-dose serology sample at V04 in the proper time window (outside 28-35 days range since vaccination 3) or a post-dose serology sample was not drawn
- Subject's serology sample at V04 did not produce a valid test result for Hep B.
- Subject's serology samples at V01 or V04 did not produce a valid test result for PT or FIM.
- Subject received a protocol-prohibited therapy / medication / vaccine (from Categories 2 and 3 as defined in Section 6.7)

In addition to the reasons listed above, subjects will also be excluded from the PPAS if their serology sample did not produce a valid test result (i.e., results for all antigens are missing).

In the event of a local or national immunization program e.g. with national immunization with polio vaccine, subjects who receive one or more doses of such vaccine at any time during the trial will not be withdrawn from the trial. If the Home Visit/OPD Visit (HV/OPDV) happens later than 9th day, Protocol deviation will not be filed and the same will be documented in the medical chart.

This list may not be exhaustive. The above protocol deviations leading to exclusion from the PPAS may be detailed and completed in the Statistical Analysis Plan (SAP) following a data review (if necessary). The PPAS definition will be finalized before the database lock.

12.2.4 Populations Used in Analyses

The primary immunogenicity analyses (non-inferiority testing on Stage 1) will be performed on the PPAS analysis set, and will be confirmed on the FAS. The secondary immunogenicity descriptive analyses for Stage 1 will also be performed on PPAS and FAS. In the FAS, for stage1, subjects will be analyzed by the vaccine group to which they were randomized.

For stage 2, the immunogenicity will be performed on the FAS and described overall and according to the randomized vaccine group at Stage 1.

The safety analysis will be performed on the SafAS. Subjects will be analyzed according to the vaccine they actually received at the corresponding Stage.

12.3 Handling of Missing Data and Outliers

12.3.1 Safety

Missing data will not be imputed. No replacement will be done.

12.3.2 Immunogenicity

For computational purposes, any pre-vaccination or post-vaccination concentration reported as < lower limit of quantification (LLOQ) will be converted to a value of 0.5 LLOQ. All values identified with a mathematical symbol "above" or "above or equal" to the upper limit of quantification ("> ULOQ" or "≥ ULOQ") will be truncated as ULOQ.

Missing data will not be imputed. No test or search for outliers will be performed (immunogenicity data will be considered as validated).

12.3.3 Efficacy

Not applicable.

12.4 Interim / Preliminary Analysis

No interim analyses are planned.

The statistical analysis will be performed in 2 steps: one on the data collected during Stage 1 (the V01-V04 period), and the final one at the end of Stage 2.

12.5 Determination of Sample Size and Power Calculation

A total of 460 subjects will be enrolled in the study. Subjects will be randomly allocated to one of the two groups (iSHAN 5 or cSHAN 5) using the 1:1 ratio.

The sample size is calculated based on primary study objectives, with an alpha level of 2.5% (one-sided hypotheses), a 10% non-inferiority clinical margin for the hepatitis B responses, a 2-fold ratio for aGMC ratio for pertussis responses (PT and FIM) and an assumption of 85% of enrolled subjects fulfilling the Per Protocol definitions in each group.

The sample size calculations are based on the following assumptions:

**Endpoints**	**Ref std(log10) or seroprotection rate***	**ratio or δ for NI**	**With unilateral alpha 0.025**
			**Power for NI**

			**N=390**
**Pertussis : anti-PT**	log sd aGMC=0,75	2	97,7%
**Pertussis : anti-FIM**	log sd aGMC=0,85	2	93,7%
**Anti-HBs ≥ 10 mIU/ml**	95%	10%	98,4%
**Overall power**			90,1%

* reference level were observed on previous study samples results.

Based on simulations, testing the null hypothesis on Pertussis responses with a power of 91.5% (on aGMCs at Day 84), and assuming observed standard deviations of log10(aGMCs) of 0.75 and 0.85, respectively on PT and FIM results, will require a total of 195 evaluable subjects in each group. With such a sample size and an assumption of 95% of subjects with an anti-HBs Ab concentration ≥10 mIU/mL at D84, the power to demonstrate non-inferiority on HepB will be 98.4% (using the Farrington and Manning method) meaning that the overall power of the trial is at least 90%.

Considering a 15% of subjects non-evaluable at D84, a total of 460 subjects should be included in the trial to reach the primary objective with an overall power of at least 90%.

Based on subject retention rates observed in similar booster studies conducted previously by Shantha, a minimum of 322 subjects (70% of subjects enrolled in Stage 1) will be ensured to enroll in Stage 2. For the descriptive assessment of the booster immune response, a subset of 322 subjects will ensure from +/-5.0% to 3.3% of variability around expected seroprotection / seroconversion rates from 70% to 90%.

13 Ethical and Legal Issues and Investigator / Sponsor Responsibilities

13.1 Ethical Conduct of the Study / Good Clinical Practice

The conduct of this study will be consistent with the standards established by the Declaration of Helsinki and compliant with the ICH guidelines for GCP as well as with all local and / or national regulations and directives.

13.2 Source Data and Source Documents

"Source data" are the data contained in source documents. Source documents are original documents or certified copies, and include, but are not limited to, diary cards, medical and hospital records, screening logs, informed consent / assent forms, telephone contact logs, and worksheets. The purpose of study source documents is to document the existence of subjects and to substantiate the integrity of the study data collected. Investigators must maintain source documents so that they are accurate, complete, legible, and up to date.

For missing or discrepant data on a diary card, the study coordinator will obtain verbal clarification from the subject, enter the response into the "investigator's comment" page of the diary card, and transfer the information to the CRB.

The subject pre-screening log should list all individuals contacted by the Investigators to participate in the study, regardless of the outcome.

Good Documentation Practice should be followed by the Investigator and the site staff managing source documents.

13.3 Confidentiality of Data and Access to Subject Records

Prior to initiation of the study, the Investigator will sign a fully executed confidentiality agreement with Shantha.

Shantha personnel (or designates), the IECs / IRBs, and regulatory agencies, including the DCGI, require direct access to all study records, and will treat these documents in a confidential manner.

In the event a subject's medical records are not at the investigational site, it is the responsibility of the investigator to obtain those records if needed.

13.4 Monitoring, Auditing, and Archiving

13.4.1 Monitoring

Before the start of the study (i.e., before the inclusion of the first subject), the Investigators and the Sponsor's staff will meet at the site-initiation visit to discuss the study protocol and the detailed study procedures. Emphasis will be placed on inclusion and exclusion criteria, visit timing, safety procedures, informed consent procedures, SAE reporting procedures, CRB completion, and the handling of samples and products. The Sponsor's staff will ensure and document that all material to be used during the study has been received at the site; and that the study investigator team have been properly informed about the study, GCP and regulatory requirements, and the Sponsor's procedures. Specific training sessions for the study investigator team on these topics may be performed as necessary, and should be documented.

The following instruction manuals will be provided: the CRB Completion Instructions for entering data into the CRB, and the Operating Guidelines for detailed study procedures such as the product management and sample-handling procedures.

After the start of the study, the Sponsor's staff will be in regular contact with the investigational team through telephone calls and regular follow-up visits. The Investigator or delegate must be available for these visits, and must allow the Sponsor staff direct access to subject medical files and CRBs. During these visits, the Sponsor staff will:

Evaluate the quality of the study progress (adherence to protocol and any study-specific guidelines, quality of data collection and document completion, signature of consent forms, occurrence of SAEs, sample and product management, cold-chain monitoring, archiving)

Source-verify completed CRBs and any corresponding answered queries

Determine the number of complete or ongoing issues identified at monitoring visits (e.g., protocol deviations, SAEs). Any identified problems will be discussed with the Investigator, and corrective or preventive actions will be determined, as appropriate.

After all protocol procedures have been completed and the data have been entered into the CRB, the Investigator must still be available to answer any queries forwarded by the Sponsor. All data-related queries must be completed prior to database lock.

At the end of the study, a close-out visit will be performed to ensure that:

The center has all the documents necessary for archiving

All samples have been shipped to the appropriate laboratories

All unused materials and products have been either destroyed or returned to the Sponsor

13.4.2 Audits and Inspections

A quality assurance audit may be performed at any time by the Sponsor’s Clinical Quality Assessment department (CQA) or by independent auditors to verify that the study has been conducted according to the protocol, GCP and ICH requirements, and other applicable regulations. An inspection may be conducted by regulatory authorities. The Investigator must allow direct access to study documents during these inspections and audits.

13.4.3 Archiving

The Investigator must keep all study documents after the completion or discontinuation of the study, whatever the nature of the investigational center (private practice, hospital, or institution), for as long as required by applicable laws and regulations. In the absence of any applicable laws or regulations, study documents will be kept at a minimum for the duration indicated on the Clinical Trial Agreement (CTA). In no event, should study personnel destroy or permit the destruction of any study documents upon less than 90 days advance written notification to the Sponsor. In addition, study documents should continue to be stored, at Sponsor's sole expense, in the event that the Sponsor requests in writing that such storage continues for a period of time that exceeds that required by any applicable law or regulation or the CTA. The Investigator will inform Shantha of any address change or if they will no longer be able to house the study documents.

Archived data may be held on electronic records, provided that a back-up exists and that a hard copy can be obtained if required. The protocol, documentation, approvals, and all other documents related to the study will be kept by the Sponsor in the Trial Master File (TMF). Data on AEs are included in the TMF. All data and documents will be made available if requested by relevant authorities.

13.5 Financial Contract and Insurance Coverage

A Clinical Trial Agreement will be signed by all the parties involved in the study’s performance, if relevant. The Sponsor has an insurance policy to cover any liabilities that may arise from use of the product and / or the study protocol.

13.6 Stipends for Participation

Subject's parent/LAR will not be provided any financial benefit or incentive for the participation of their child in this research. However, they will be reimbursed for their travel expenses (e.g. bus tickets, parking etc.) for each visit. The expenses towards vaccination and investigations fee will be taken care of by the study sponsor till the end of study period.

13.7 Publication Policy

Data derived from this study are the exclusive property of SBPL. Any publication or presentation related to the study must be submitted to SBPL for review before submission of the manuscript. After publication of the results of the study, any participating center may publish or otherwise use its own data provided that any publication of data from the study gives recognition to the study group. In addition, SBPL shall be offered an association with all such publications, it being understood that SBPL is entitled to refuse the association.

SBPL must have the opportunity to review all proposed abstracts, manuscripts, or presentations regarding this study at least 90 days prior to submission for publication / presentation. Any information identified by SBPL as confidential must be deleted prior to submission, it being understood that the results of this study are not to be considered confidential.

SBPL's review can be expedited to meet publication guidelines.

14 Reference List

1. http://www.who.int/. [Online].; 2018 [cited 2018 May 23. Available from: http://www.who.int/news-room/fact-sheets/detail/immunization-coverage.
2. Obaro S, Palmer A. Vaccines for children: policies, politics and poverty. Vaccine. 2003; 21: p. 1423-31.
3. Pichichero M. New combination vaccines. Pediatr Clin North Am. 2000; 47: p. 407-26.
4. Edwards K, Decker M. Combination vaccines: hope and challenges. Pediatr InfectDis J. 1994; 13: p. 345-7.
5. Clarke KEN. http://www.who.int/. [Online]. [cited 2018 May 23. Available from: http://www.who.int/immunization/sage/meetings/2017/april/1_Final_report_Clarke_april3.pdf.
6. WHO. Diphtheria vaccine: WHO position paper – August 2017. 2017; 92: p. 417–436.
7. [Online]. [cited 2018 June 4. Available from: http://www.who.int/immunization/monitoring_surveillance/data/gs_gloprofile.pdf?ua=1.
8. [Online]. [cited 2018 June 4. Available from: http://www.who.int/immunization/monitoring_surveillance/data/gs_afrprofile.pdf?ua=1.
9. [Online]. [cited 2018 June 4. Available from: http://apps.who.int/immunization_monitoring/globalsummary/countries?countrycriteria%5Bcountry%5D%5B%5D=IND&commit=OK.
10. WHO. Tetanus vaccines: WHO position paper – February. Weekly epidemiological record. 2017: p. 53–76.
11. Forsyth KD, Wirsing von Konig CH. Prevention of pertussis: Recommendations derived from the second Global Pertussis Initiative roundtable meeting. Vaccine. 2007; 25(14): p. 2634-2642.
12. WHO. Pertussis vaccines: WHO position paper – August 2015. Weekly epidemiological record. 2015: p. 433-460.
13. WHO. Haemophilus influenzae type b (Hib) Vaccination WHO Position Paper – September 2013. Weekly epidemiological record. 2013: p. 413-428.
14. Vashishtha VM. Introduction of Hib Containing Pentavalent Vaccine in National Immunization Program of India: The Concerns and the Reality! Indian Pediatrics. 2009; 46: p. 781-782.
15. Eskola J, Peltola H, Kayhty H, Takala AK, Makela PH. Finnish efficacy trials with Haemophilus influenzae type b vaccines. J Infect Dis. 1992;: p. S137-S138.

16. N G. Hepatitis B: diagnosis, prevention, and treatment. Clinical Chemistry. 1997; 43: p. 1500-1506.
17. WHO. Hepatitis B vaccines: WHO position paper – July 2017. Weekly epidemiological record. 2017 July 7: p. 369–392.
18. Kew MC. Epidemiology of chronic hepatitis B virus infection, hepatocellular carcinoma, and hepatitis B virus-induced hepatocellular carcinoma. Pathologie Biologie. 2010; 58(4): p. 273-277.
19. Rao R, Dhingra MS, Bavdekar S, Behera N, Daga SR, Dutta AK, et al. A comparison of immunogenicity and safety of indigenously developed liquid (DTwPHB-Hib)pentavalent combination vaccine (Shan 5) with Easyfive (Liq) and Tritanrix + Hiberix (Lyo) inIndian infants administered according to the EPI schedule. Human Vaccines. 2009; 5(6): p. 425-429.
20. Gandhi DJ, Dhaded SM, Ravi MD, Dubey AP, Kundu R, Lalwani SK, et al. Safety, immune lot-to-lot consistency and non-inferiority of a fully liquid pentavalent DTwp-HepB-Hib vaccine in healthy Indian toddlers and infants. Hum Vaccin Immunother. 2016 Apr 2; 12(4): p. 946-54.
21. Shantha Biotechnics Data on File: SH505 CSR..
22. Sejvar JJ, Kohl KS, Bilynsky R, Blumberg D, Cvetkovich T, Galama J, et al. Encephalitis, myelitis, and acute disseminated encephalomyelitis (ADEM): Case definitions and guidelines for collection, analysis, and presentation of immunization safety data. Vaccine. 2007; 25(31): p. 5771-5792.
23. IOM (Institute of Medicine).. Adverse effects of vaccines: Evidence and causality Washington, DC: The National Academies Press.; 2012.
24. Dolan S, Wallace A, Burnett E, Ehlman D, Sui W, Garon J. http://www.who.int/. [Online].; 2015 [cited 2018 May 22. Available from: http://www.who.int/immunization/sage/meetings/2015/april/5_Summary_of_Evidence_3-25-2015.pdf.
25. Bharat Biotech. [Online]. [cited 2018 Oct 8. Available from: https://www.bharatbiotech.com/images/biopolio/BiopolioB13PackageInsert.pdf.
26. [Online]. [cited 2018 May 22. Available from: https://www.bharatbiotech.com/images/rotavac/rotavac_presc_inform.pdf.
27. [Online]. [cited 2018 May. Available from: http://www.vaxinpoint.in/wp-content/uploads/2014/02/Tresivac-Manufactured-Product-Serum-Institute-of-India.pdf.
28. Gold MS, Bayas JM. Anaphylaxis: case definition and guidelines for data collection, analysis and presentation of immunization data. Vaccine. 2007; 25: p. 5675-5684.

29. Bonhoeffer J, Menkes J, Gold MS. Generalized convulsive seizure as an AE following immunization: case definition and guidelines for data collection, analysis and presentation. Vaccine. 2004; 22: p. 557-566.

30. Vermeer-de Bondt PE. Performance of the Brighton Collaboration case definition for hypotonic-hyporesponsive episode (HHE) on reported collapse reactions following infant vaccinations in the Netherland. Vaccine. 2006; 24: p. 7066-7070.

31. Buettcher M, Heininger U, Braun M. The Brighton collaboration HHE working group Hypotonic Hyporesponsive Episode (HHE) as an adverse event following immunization: case definition and guidelines for data collection, analysis and presentation. Vaccine. 2007; 25: p. 5875-5881.

32. Sejvar JJ. Encephalitis, myelitis, and acute disseminated encephalomyelitis (ADEM): Case definitions and guidelines for collection, analysis, and presentation of immunization safety data. Vaccine. 2007; 25: p. 5771-5792.

33. Kapil P, Papin JF, Wolf RF, Zimmerman LI, Wagner LD, Merkel TJ. Maternal Vaccination With a Monocomponent Pertussis Toxoid Vaccine Is Sufficient to Protect Infants in a Baboon Model of Whooping Cough. The Journal of Infectious Diseases. 2018 March; 217(8): p. 1231–1236.

34. Carbonetti NH. Contribution of pertussis toxin to the pathogenesis of pertussis disease. FEMS Pathogens and Disease. 2015 Nov; 73(8).

35. WHO. WHO Expert Committee on Biological Standardization: Fifty-sixth Report. WHO Technical Report Series. Geneva: WHO; 2007. Report No.: 941.

36. Fredriksen H, Namork , Frøholm LO. Immuno-electronmicroscopy of fimbriae-like structures on Bordetella pertussis serotype 1.3. J Med Microbiol.. 1988 Apr; 25(4): p. 285-8.

37. Miller J, Silverberg J, Saito TM, Humber B. An agglutinative reaction for Hemophilus pertussis, II: its relation to clinical immunity. J Pediatr. 1943; 22: p. 644-651.

38. Sako. Studies on pertussis immunization. J Pediatr. 1947; 30: p. 29-40.

39. Langue J, Ethevenaux C, Chamosaur A, Fritzell B, Begue P, Saliou P. Safety and Immunogenicity of Haemophilus influenzae type b-tetanus toxoid conjugate, presented in a dual chamber syringe with diphtheria-tetanus-pertussis and inactivated poliomyelitis combination vaccine. Eur J Pediatr. 1999; 158: p. 7717-722.

15 Signature Page
